# *Klebsiella pneumoniae* inhibits vasodilation through capsule and T6SS-dependent pathways

**DOI:** 10.1038/s41564-026-02425-0

**Published:** 2026-07-21

**Authors:** Safi Rehman, Joana Sa-Pessoa, Charlotte Buckley, Rachel L. Lee, Karolina Wojtania, Alix Lee, Rebecca Lancaster, Josy Augustine, Peter Barabas, Ciara Ross, Xun Zhang, John G. McCarron, Tim M. Curtis, Jose A. Bengoechea

**Affiliations:** 1 Wellcome–Wolfson Institute for Experimental Medicine, School of Medicine, Dentistry and Biomedical Sciences, Queen’s University Belfast, Belfast, UK; 2 Department of Infection Biology, London School of Hygiene and Tropical Medicine, London, UK; 3 Strathclyde Institute of Pharmacy and Biomedical Sciences, University of Strathclyde, Glasgow, UK

**Keywords:** Pathogens, Bacterial pathogenesis

## Abstract

Vasodilation is a defence mechanism during inflammation and infection that is regulated by nitric oxide produced by endothelial nitric oxide synthase (eNOS) and the generation of endothelium-dependent hyperpolarization. Several viral infections have been shown to alter vascular biology, but effects of bacterial pathogens are unclear. Here using an ex vivo blood vessel model and human primary endothelial cells, we show that *Klebsiella pneumoniae*, a prevalent bloodstream pathogen, inhibits vasodilation pathways. The type VI secretion system (T6SS) effector VgrG4 activates the mitochondrial receptor NLRX1, which leads to increased mitochondrial reactive oxygen species and phosphorylation of the eNOS inhibitory site by the kinase PKCβ, which in turn reduces eNOS activity. *K.* *pneumoniae* capsule polysaccharide also activates the phosphatase PP2Ac, which reduces phosphorylation of the eNOS activation site. VgrG4-induced mitochondrial reactive oxygen species attenuate endothelium-dependent hyperpolarization by impairing signalling of the Ca^2+^-activated K^+^ channel axis. This work reveals that T6SS activity can modulate host vascular biology by targeting eNOS post-translational modifications.

## Abstract (web summary)

Vasodilation is inhibited by *Klebsiella pneumonia* through a multipronged approach involving type VI secretion system activity and the VgrG4 effector, plus capsule, to limit eNOS-dependent and endothelial hyperpolarization routes to vasodilation.

## Main

Vascular endothelial cells are central to vascular homeostasis and they govern blood flow, haemostatic balance, coagulation, vessel-wall permeability and leukocyte recruitment. Endothelial dysfunction, characterized by impaired vasodilation and a prothrombotic phenotype, underpins the pathophysiology of atherosclerosis, angiogenesis in cancer, vascular leakage, stroke and infectious diseases^1^. Multiple viral pathogens, most dramatically haemorrhagic viruses such as Ebola, Lassa and Marburg, exploit this vulnerability^2^. However, the mechanisms by which bacterial pathogens perturb vascular physiology beyond the broad context of sepsis-associated endothelial dysfunction remain poorly understood^3^.

Endothelium-dependent vasodilation depends on the bioavailability of nitric oxide (NO) synthesized from L-arginine by the constitutively expressed eNOS^4,5^. eNOS activity is strictly dependent on Ca^2+^/calmodulin^6,7^; therefore, an increase in intracellular, free Ca^2+^ concentration is necessary for endothelial NO production to trigger vasodilatation^7,8^. After synthesis, NO diffuses into vascular smooth muscle cells where it activates soluble guanylyl cyclase to form cyclic guanosine monophosphate^5^. Loss of eNOS function increases susceptibility to atherosclerosis, hypertension, thrombosis, sepsis and stroke, which reflects the crucial role of NO and vasodilation in vascular biology. Endothelium-dependent hyperpolarization (EDH)^9^ provides a complementary vasodilatory mechanism, particularly in small arteries, wherein Ca^2+^-activated small (SKCa) and intermediate conductance (IKCa) Ca^2+^-activated K^+^ channels hyperpolarize the endothelium with current propagating to the vascular smooth muscle through myoendothelial gap junctions^9^.

Bloodstream infections (BSIs) carry a crude mortality rate of up to 30%, and antibiotic-resistant pathogens disproportionately drive admission to intensive care units and cause death^10–14^. *K.* *pneumoniae* is the second most prevalent Gram-negative pathogen in BSIs, with mortality reaching 79% in some cohorts^10,14,15^. Despite the clinical relevance of *Klebsiella*-induced BSI, there is a gap in our knowledge regarding the interface between *K.* *pneumoniae* and the vasculature. Notably, *K.* *pneumoniae*-mediated pneumosepsis recapitulates hallmarks of acute respiratory distress syndrome, including capillary endothelial injury, alveolar oedema and vascular leakage. Clinical observations have linked *K.* *pneumoniae* infection with hypertension^16^, and preclinical data demonstrate limited vasodilation in arteries of mice infected with *K.* *pneumoniae*^16^. Altogether, this evidence implicates endothelial dysfunction as a pathophysiologically significant and mechanistically unexplained feature of *K.* *pneumoniae* pathophysiology.

Here we leverage a research platform that integrates an ex vivo blood vessel model and human primary endothelial cells to dissect the molecular mechanisms by which *K.* *pneumoniae* disrupts vascular function. We demonstrate that *K.* *pneumoniae* inhibits agonist-induced vasodilation by simultaneously suppressing eNOS activity through two independent pathways. The type VI secretion effector VgrG4 induces phosphorylation of the eNOS(Thr495) inhibitory site via PKCβ, downstream of NLRX1-dependent mitochondria reactive oxygen species (mtROS) signalling. By contrast, the capsule polysaccharide (CPS) suppresses activating phosphorylation at eNOS(Ser1177) through an axis that involves EGF receptor (EGFR) and the phosphatase PP2Ac. These findings identify *K.* *pneumoniae* as a direct antagonist of vascular NO biology and reveal previously unrecognized pathogen-driven signalling cascades with broad implications for infection-associated vascular pathology.

## Results

### *K.pneumoniae* inhibits agonist-induced vasodilation

**Fig. 1:**
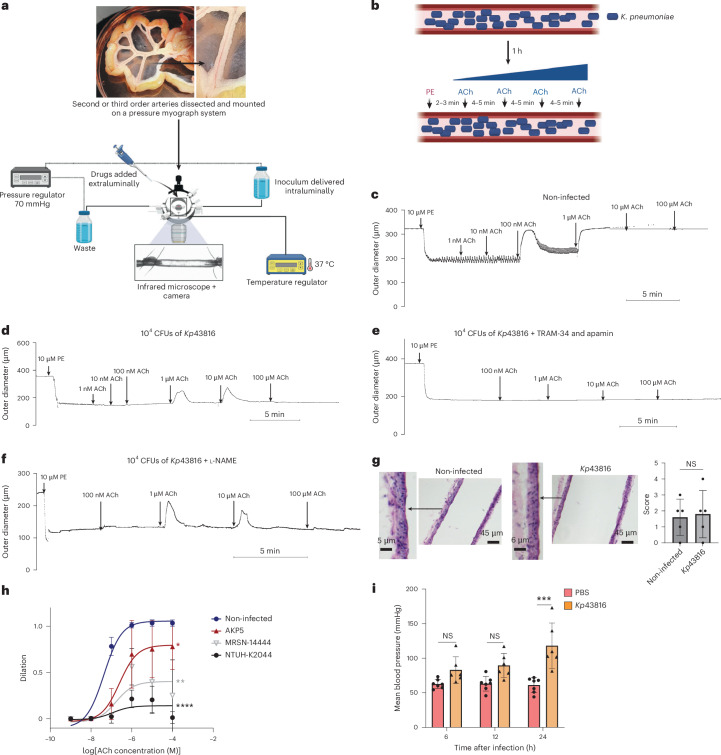
*K.* *pneumoniae* inhibits ACh-induced vasodilation. **a**, Schematic of the ex vivo blood vessel model. **b**, Schematic of the experimental workflow. **c**, Representative trace of ACh-induced vasodilation in PE-constricted vessels. **d**, Representative trace of *Kp*43816-triggered inhibition of ACh-induced vasodilation in PE-constricted vessels. **e**, Representative trace of the effect of the EDH pathway inhibitors TRAM-34 and apamin on *Kp*43816-triggered inhibition of ACh-induced vasodilation in PE-constricted vessels. **f**, Representative trace of the lack of effect of the NO pathway inhibitor L-NAME on *Kp*43816-triggered inhibition of ACh-induced vasodilation in PE-constricted vessels. **g**, Left, representative images of non-infected and infected vessels stained using haematoxylin and eosin. Right, scoring of blood vessel pathology. **h**, Dose–response curves of ACh-induced peak dilation in non-infected and infected vessels with the strains AKP5, MRSN-14444 and NTUH-K2044. **i**, Blood pressure, expressed as mmHg, in PBS mock-infected and *Kp*43816-infected mice at the indicated times. In **c** –**f**, the traces are representative of six vessels from three rats (two per rat). In **g**, images are representative of five vessels from five different rats (one per rat), and histopathology quantification is shown as the mean ± s.d. and analysed using two-sided Mann–Whitney *t*-tests. NS, not significant (*P* > 0.05). In **h**, peak dilation is shown as the mean ± s.d. from six vessels from three rats (two per rat) per group and analysed using two-way analysis of variance (ANOVA) and Dunnet’s multiple group comparison correction. In **i**, six mice per group were followed over time in two independent infections and analysed using two-way ANOVA and Dunnet’s multiple group comparison correction. Results of individual mice are displayed with bars representing the median ± s.d. In all panels, *P < 0.05, **P < 0.01, ***P < 0.001, ****P < 0.0001. Schematics created in BioRender: **a**, Alphonse, N. https://biorender.com/buanb89 (2026); **b**, Pessoa, J. https://biorender.com/23s4hyg (2026). Source data

**Extended Data Fig. 1:**
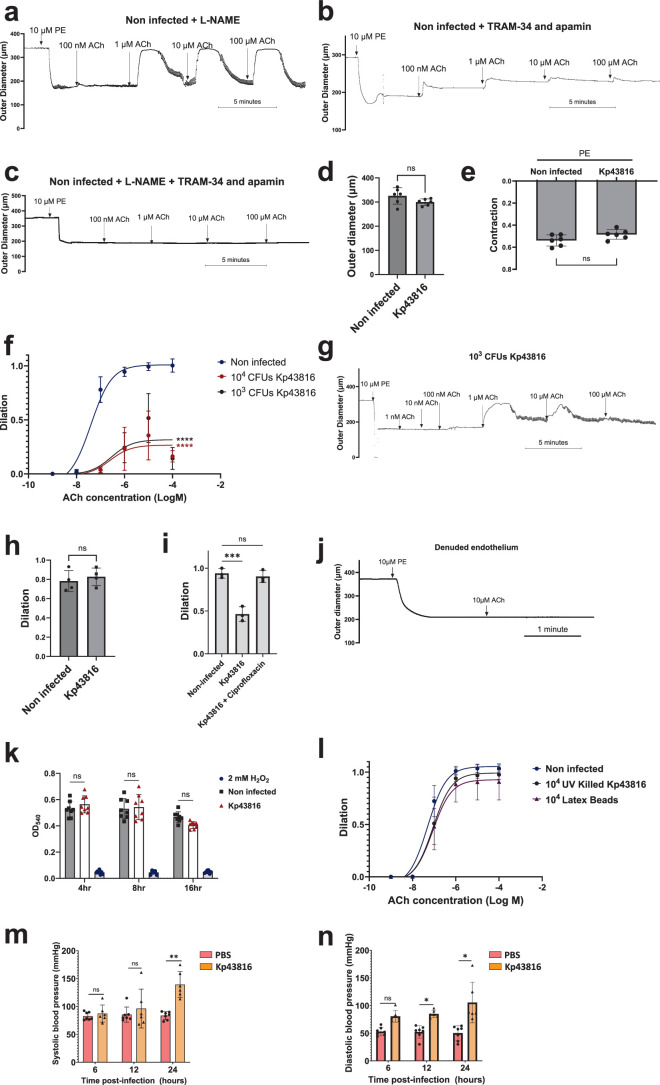
*K. pneumoniae* inhibits ACh-induced vasodilation. **a**. Representative trace of the effect of NO pathway inhibitor L-NAME on ACh-induced vasodilation in non-infected PE-constricted vessels. **b**. Representative trace of the effect of EDH pathway inhibitors TRAM-34 and apamin on ACh-induced vasodilation in non-infected PE-constricted vessels. **c**. Representative trace of the effect of NO and EDH pathway inhibitors L-NAME, TRAM-34 and apamin on ACh-induced vasodilation in non-infected PE-constricted vessels. **d**. Outer diameter of non-infected and infected vessels for 1 h with Kp43816. **e**. Outer diameter of the non-infected and infected vessels for 1 h with Kp43816 after PE-induced vasoconstriction. **f**. Concentration response of ACh-induced peak dilation in non-infected and infected vessels with 10^4^ and 10^3^ CFUs of strain *Kp*43816. **g**. Representative trace of Kp43816 (10^3^ CFUs)-triggered inhibition of ACh-induced vasodilation in PE-constricted vessels. **h**. Peak dilation of non-infected and infected vessels treated with the NO donor SNP in PE-constricted vessels. **i**. Peak dilation of non-infected and infected vessels treated or not with ciprofloxacin in PE-constricted vessels. **j**. Representative trace of the lack of ACh- induced vasodilation in vessels in which the endothelium was damaged by perfusion of a stream of air. **k**. Endothelial cell viability assessed by quantifying the binding of the supravital dye neutral red to the lysosomes at the indicates tome points. **l**. Dose response of ACh-induced peak dilation in non-infected and infected vessels with UV-killed *Kp*43186 and 10^4^ latex beads. **m**. Systolic pressure, expressed as mmHg, in PBS mock-infected and Kp43816 infected mice at the indicated times. **n**. Diastolic pressure, expressed as mmHg, in PBS mock-infected and *Kp*43816 infected mice at the indicated times. In **a**, **b**, **c**, **g** and **j** the traces are representative of six vessels from three rats (two per rat). In **f** and **i** peak dilation is shown as mean ± SD from six vessels from three rats (two per rat) per group. In d and h, dilation is shown as mean ± SD from six and four vessels, respectively, from three rats per group. In **e**, constriction is shown as mean ± SD from six vessels from three rats (two per rat) per group. In f and i, data were analysed with two-way ANOVA and Dunnet’s multiple group comparison correction. In **m** and **n**, 6 mice per group were followed over time and analysed with two-way ANOVA and Dunnet’s multiple group comparison correction. In **d**, **e** **h**, and **k** data was analysed using an unpaired, two-tailed student’s t-test. The asterisks indicate p < 0.05 (*), < 0.001 (***), or p < 0.0001 (****).; ns, not significant.

To investigate infection-induced vascular effects, we developed an ex vivo model using rat mesenteric arteries mounted in a pressure myograph system, which enabled real-time measurement of changes in vasoconstriction and vasodilation via the outer vessel diameter (Fig. 1a,b and Methods). In control vessels, the addition of phenylephrine (PE) induced constriction, and subsequent addition of acetylcholine (ACh) triggered endothelial vasodilation mediated by muscarinic M_3_ receptors^17–19^ (Fig. 1c and Supplementary Video 1). Notably, 100 nM ACh induced transient vasodilation (Fig. 1c and Supplementary Video 1), whereas 1 μM restored the basal diameter of the vessel (Fig. 1c and Supplementary Video 2). As previously reported^9,20^, treatment of vessels with L-NAME, a NO pathway inhibitor, resulted in transient ACh-induced vasodilation that reached the basal diameter of the vessel (Extended Data Fig. 1a). In turn, inhibition of the EDH pathway with the IKCa channel inhibitor TRAM-34 and the SKCa channel inhibitor apamin resulted in small, sustained increases in vasodilation with increasing concentrations of ACh (Extended Data Fig. 1b). However, these increases never reached the basal diameter of the vessel (Extended Data Fig. 1b). Combined inhibition of NO and EDH pathways eliminated ACh-induced vasodilation (Extended Data Fig. 1c), a result that confirms that both pathways are required to induce this effect.

We next tested whether the *K.* *pneumoniae* strain ATCC43816 (hereafter termed *Kp*43816), which encodes all loci found in strains associated with invasive community-acquired infections^21^, alters vascular function. Intraluminal infection with 10^4^ colony-forming units (CFUs) did not affect the outer diameter of the vessel (Extended Data Fig. 1d) or PE-induced vasoconstriction (Extended Data Fig. 1e). However, it markedly inhibited ACh-induced vasodilation (Fig. 1d, Extended Data Fig. 1f and Supplementary Videos 3 and 4). A total of 1-log fewer bacteria also inhibited ACh-triggered vasodilation (Extended Data Fig. 1f,g). TRAM-34 and apamin (Fig. 1e), but not L-NAME (Fig. 1f), abrogated the residual ACh-induced vasodilation in *Kp*43816-infected vessels, which suggests that *K.* *pneumoniae* abolishes NO-dependent vasodilation. In addition, EDH-mediated vasodilation was significantly diminished when compared with control vessels. The NO donor sodium nitroprusside (SNP) restored vasodilation in infected vessels to control levels (Extended Data Fig. 1h), which demonstrated the intact muscle responsiveness to NO. Histopathology analyses showed preserved endothelial and smooth muscle layers (Fig. 1g). As a further demonstration that infection does not result in overt vascular damage, ACh-induced vasodilation was not significantly different between non-infected vessels and those infected and subsequently treated with ciprofloxacin to eliminate bacteria (Extended Data Fig. 1i). Control experiments showed that ACh did not induce vasodilation in vessels in which the endothelium was denuded by intraluminal bubbling of air (Extended Data Fig. 1j). Infection did not affect the viability of immortalized human lung microvascular endothelial cells (HULEC-5a; hereafter termed HULECs) (Extended Data Fig. 1k). Meanwhile, 10^4^ CFUs of UV-killed bacteria or 10^4^ latex beads similar in size to *Klebsiella* did not affect ACh-dependent vasodilation (Extended Data Fig. 1l). This result ruled out the possibility that the mere presence of bacteria has a detrimental effect on ACh-induced vasodilation. NTUH-K2044 (ref. ^22^), a clinical isolate used to analyse *K.* *pneumoniae* metastatic strains found in Asia, and the clinical isolates AKP5 and MRSN-14444 also inhibited ACh-induced vasodilation (Fig. 1h). This finding reveals that *Klebsiella* inhibition of ACh-mediated vasodilation does not depend on the strain, although it is worth noting that some strains inhibited dilation to a higher degree than others.

The ex vivo results suggest that *K.* *pneumoniae* should have a profound effect on vascular function in vivo, which may lead to hypertension. Clinical studies have found an association between hypertension and *K.* *pneumoniae* infection^16^. To ascertain the in vivo effect of *K.* *pneumoniae* on vascular physiology, we measured blood pressure in mice after intraperitoneal infection. *K.* *pneumoniae* infection triggered an increase in blood pressure at 24 h after infection compared with PBS-treated controls. At 6 and 12 h after infection, infected mice also showed increased blood pressure, although these differences did not reach significance (Fig. 1i). By 24 h after infection, mean systolic pressure values reached 140 mmHg and mean diastolic pressure values reached 100 mmHg, which indicated a clear hypertensive shift relative to controls (Extended Data Fig. 1m,n).

Combined, these data demonstrate that live *K.* *pneumoniae* abrogates ACh-induced vasodilation in mesenteric arteries by targeting both NO and EDH pathways, without damaging vascular structure or smooth muscle function. Moreover, this vascular dysfunction translates into *K.* *pneumoniae*-associated hypertension in vivo.

### VgrG4 is the T6SS effector inhibiting vasodilation

**Fig. 2:**
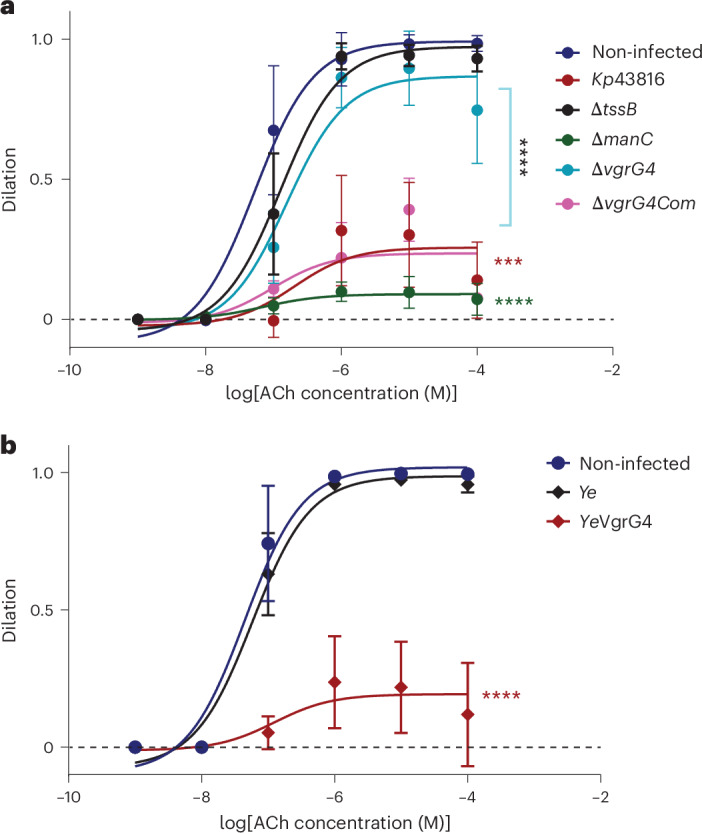
The T6SS effector VgrG4 inhibits ACh-induced vasodilation. **a**, Concentration–response curves of ACh-induced peak dilation in non-infected and infected vessels with the strains *Kp*43186, *cps* mutant (Δ*manC*; 43816-Δ*manC*) and T6SS mutants *tssB* (Δ*tssB*; 43816-Δ*tssB*) and *vgrG4* (Δ*vgrG4*; 43816-Δ*vgrG4*). Δ*vgrG4Com* is a *vgrG4* complemented strain. **b**, Concentration–response curves of ACh-induced peak dilation in non-infected vessels and in vessels infected with the control strain of *Y. enterocolitica* (*Ye*) and a strain expressing VgrG4 (*Ye*VgrG4). In **a** and **b**, peak dilation is shown as the mean ± s.d. from six vessels from three rats per group and analysed using two-way ANOVA and Dunnet’s multiple group comparison correction. In **a**, the following significant comparisons are shown: between *Kp*43816-infected vessels and non-infected vessels (red asterisks); between Δ*manC*-infected vessels and non-infected vessels (green asterisks); and between Δ*vgrG4*-infected vessels and Δ*vgrG4Com*-infected vessels (black asterisks). In **b**, the following significant comparisons are shown: between *Ye*-infected vessels and *Ye*VgrG4-infected vessels (red asterisks). In all panels, ***P < 0.001, ****P < 0.0001.

**Extended Data Fig. 2:**
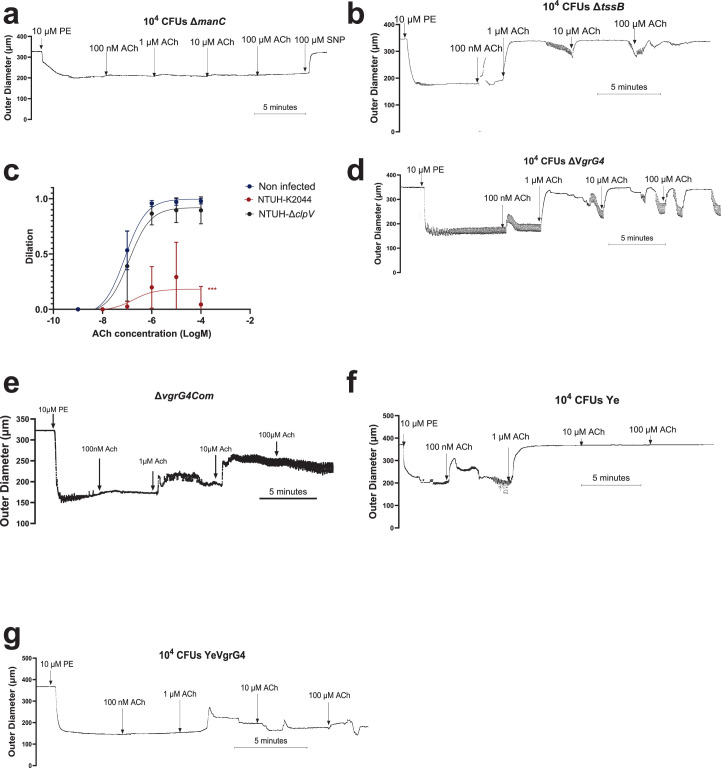
T6SS effector VgrG4 inhibits ACh-induced vasodilation. **a**. Representative trace of the lack of effect of infection with the cps mutant (Δ*manC*; 43816-Δ*manC*) on ACh-induced vasodilation in PE-constricted vessels. **b**. Representative trace of the lack of effect of infection with the *tssB* mutant (Δ*tssB*; 43816-Δ*tssB*) on ACh-induced vasodilation in PE-constricted vessels. **c**. Concentration response of ACh-induced peak dilation in non-infected and infected vessels with strains NTUH-K2044 and the T6SS mutant NTUH-Δ*clpV*. **d**. Representative trace of the lack of effect of infection with the vgrG4 mutant (Δ*vgrG4*; 43816-Δ*vgrG4*) on ACh-induced vasodilation in PE-constricted vessels. **e**. Representative trace of the complemented vgrG4 mutant-triggered inhibition of ACh-induced vasodilation in PE-constricted vessels. **f**. Representative trace of the lack of effect of infection with *Ye* on ACh-induced vasodilation in PE-constricted vessels. **g**. Representative trace of the effect of infection with *Ye*VgrG4 on ACh-induced vasodilation in PE-constricted vessels. In **a**, **b**, **d**, **e**, **f** and **g** the traces are representative of six vessels from three rats (two per rat). In c, peak dilation is shown as mean ± SD from six vessels from three rats (two per rat) per group and data were analysed with two-way ANOVA and Dunnet’s multiple group comparison correction. The asterisks indicate p < 0.001 (***).

We next sought to identify the *Klebsiella* factors responsible for the inhibition of vasodilation. Considering the crucial role of the CPS on *Klebsiella*–host interactions^23^, we investigated a *cps* mutant to assess whether the CPS is involved in the inhibition of ACh-induced vasodilation. However, the *cps* mutant still abrogated ACh-induced vasodilation (Fig. 2a and Extended Data Fig. 2a). Recently, we uncovered the role of the T6SS as an immune evasin^24,25^. We also demonstrated the activity of *Kp*43816 T6SS in antagonizing bacteria and in inducing fragmentation of mitochondria^24,26^. Consequently, we asked whether the T6SS was responsible for the *Klebsiella*-induced inhibition of vasodilation. In contrast to *Kp*43816-infected vessels, ACh-induced relaxation was not abrogated in *tssB* mutant infected vessels (Fig. 2a and Extended Data Fig. 2b). TssB is a core component of the sheath section of the T6SS apparatus, and the T6SS is not assembled in a *tssB* mutant^27–30^. The T6SS also mediated NTUH-K2044-induced inhibition of ACh-dependent vasodilation because a *clpV* mutant did not abrogate ACh-triggered vasodilation (Extended Data Fig. 2c). A *clpV* mutant cannot assemble a functional T6SS^27–30^.

Next, we aimed to identify the T6SS effector that mediates the inhibition of ACh-triggered vasodilation. We initially focused on the trans-kingdom effector VgrG4 (refs. ^24,26^) because we recently demonstrated that *Klebsiella* injects this effector into mammalian cells in a T6SS-dependent manner^24^. A *vgrG4* mutant did not impair ACh-triggered vasodilation (Fig. 2a and Extended Data Fig. 2d). Complementation of the *vgrG4* mutant restored the inhibition of ACh-induced vasodilation (Fig. 2a and Extended Data Fig. 2e). Because VgrG proteins are also known to act as cargo for other T6SS effectors^31^, it is conceivable that the inhibition of vasodilation could be mediated by other T6SS effectors delivered by VgrG4. To exclude this possibility, we took advantage of a *Yersinia* toolbox that enables the study of the cellular effects of single bacterial effectors through *Yersinia enterocolitica* type 3 secretion system (T3SS)-mediated injection into eukaryotic cells^32^. We previously confirmed that VgrG4 is secreted in conditions in which the T3SS is active^24^. Intraluminal infection of vessels with the *Y.* *enterocolitica* control strain did not affect ACh-induced vasodilation (Fig. 2b and Extended Data Fig. 2f). By contrast, *Y.* *enterocolitica* encoding VgrG4 (hereafter termed *Ye*VgrG4) abrogated ACh-induced vasodilation (Fig. 2b and Extended Data Fig. 2g). This result demonstrates that the *K.* *pneumoniae* T6SS effector VgrG4 is necessary and sufficient to inhibit ACh-triggered vasodilation.

### *K.pneumoniae* does not impair endothelial Ca^2+^ signalling

**Fig. 3:**
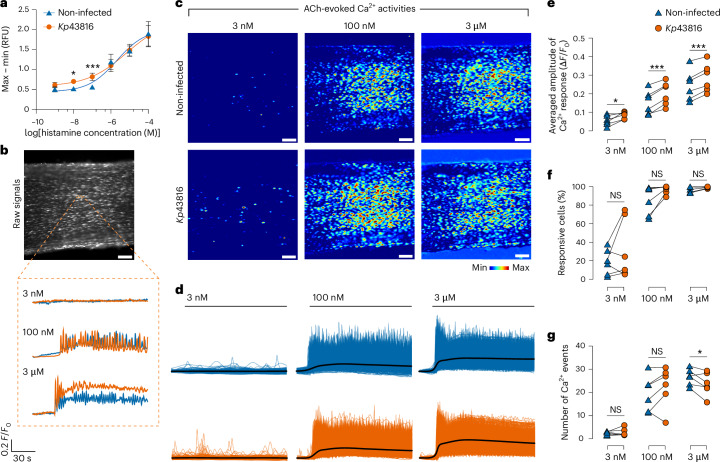
*K.* *pneumoniae* does not impair endothelial Ca^2+^ signalling. **a**, Concentration–response curves of histamine-induced intracellular Ca^2+^ in non-infected and *Kp*43816-infected HULECs (mean ± s.d. from three independent experiments in sextuplicate). **b**, Top, raw image of an en face endothelial preparation loaded with Cal-520. Scale bar, 100 µM. Bottom, examples of single Ca^2+^ signal traces (based on fluorescence (∆*F/F*_0_)) from the cell annotated in the top panel in response to ACh at concentrations of 3 nM, 100 nM and 3 µM. The image is representative of six vessels from six different rats. **c**, Heat map images of the preparation in **b**, showing maximum-intensity projections of changes in fluorescence of Ca^2+^ signals evoked by ACh in non-infected and *Kp*43816-infected endothelium. Scale bars, 100 µm. Images are representative of six vessels from six different rats. **d**, Coloured overlaid Ca^2+^ signalling traces extracted from each cell shown in **c**. The black lines show the averaged Ca^2+^ signals in response to ACh. **e**, Averaged peak amplitudes (∆*F/F*_0_). **f**, Percentage of active cells. **g**, Number of Ca^2+^ events evoked by ACh. In **e**–**g**, data are matched, with each linked dot pair representing averaged ACh responses in one vessel before (blue) and after (orange) infection with *Kp*43816. Each dot represents a different vessel from different rats. Data were analysed using paired parametric Student’s *t*-tests. In all panels, *P < 0.05, **P < 0.01, ***P < 0.001.

**Extended Data Fig. 3:**
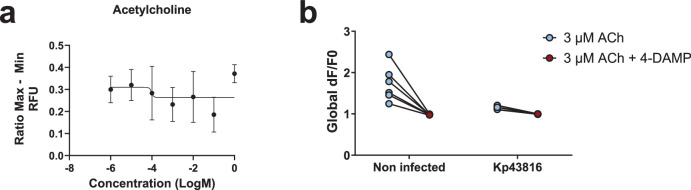
*K. pneumoniae* infection and Ca^2+^ signalling in endothelium. **a**. Concentration response of ACh-induced intracellular Ca^2+^ in non-infected HULEC cells. **b**. 4-DAMP, M3 muscarinic receptor antagonist, blunts average ACh-induced Ca^2+^ signalling in non-infected and *Kp*43816-infected vessels. In **a**, data is shown as mean ± SD from three independent experiments in sextuplicate. In **b**, each dot represents the data from one vessel before and after infection.

We next investigated how *Klebsiella* inhibits vasodilation in a T6SS VgrG4-dependent manner, focusing on endothelial Ca^2+^ signalling, which is essential for NO and EDH pathway activation^6,7,9^ and correlates linearly with vasodilation^33,34^. We first tested whether infection impairs Ca^2+^ responses in HULECs using Fura-2 dye. Histamine was used instead of ACh because HULECs do not respond to ACh (Extended Data Fig. 3a) owing to the lack of muscarinic M_3_ receptors^35^. *Kp*43816 did not reduce histamine-induced Ca^2+^ responses (Fig. 3a), and at low concentrations, responses were even enhanced (Fig. 3a). This result suggests that infection does not suppress endothelial Ca^2+^ responses.

We then assessed Ca^2+^ signalling dynamics in intact vessels using Cal-520 coupled with high spatiotemporal resolution, wide-field single-photon imaging in fields of around 1,000 cells (Fig. 3). Cells were exposed to three concentrations of ACh (3 nM, 100 nM and 3 µM) over a 200-s recording period (Fig. 3b). Multiple parameters were quantified, including the averaged response amplitude for individual cells (Fig. 3c,d), the averaged peak amplitude (Fig. 3e), the percentage of cells responding (Fig. 3f) and the frequency (number) of single-cell Ca^2+^ events (Fig. 3g). Infection did not evoke any Ca^2+^ responses (Supplementary Video 5). In control vessels, ACh elicited the expected concentration-dependent, distributed and heterogeneous responses across the endothelial population^33^ (Fig. 3b and Supplementary Video 5). Infection of the endothelium did not itself evoke a Ca^2+^ response in endothelial cells (Supplementary Video 5). Infection did not diminish ACh-evoked Ca^2+^ responses. Instead, both single-cell and population-level amplitudes of ACh-induced Ca^2+^ signals were increased in infected vessels (Fig. 3c–e and Supplementary Video 5). Furthermore, quantification of the number of cells responding to ACh (Fig. 3f) and the number of Ca^2+^ events in each cell (Fig. 3g) illustrated that *Kp*43816 infection does not reduce the ACh-induced Ca^2+^ response. Pharmacological blockade confirmed that responses were mediated by muscarinic M_3_ receptors, as 4-DAMP (1 μM) inhibited ACh-induced Ca^2+^ responses in both infected and control vessels (Extended Data Fig. 3b). These findings are consistent with previous studies demonstrating the role of these receptors in endothelial cell responses to ACh^17–19^.

Altogether, this evidence shows that ACh-evoked Ca^2+^ responses are increased in *K.* *pneumoniae*-infected vessels. Therefore, *K. pneumoniae*-dependent blockade of vasodilation cannot be explained by impaired endothelial Ca^2+^ responses.

### *K.pneumoniae* targets eNOS post-translational modifications

**Fig. 4:**
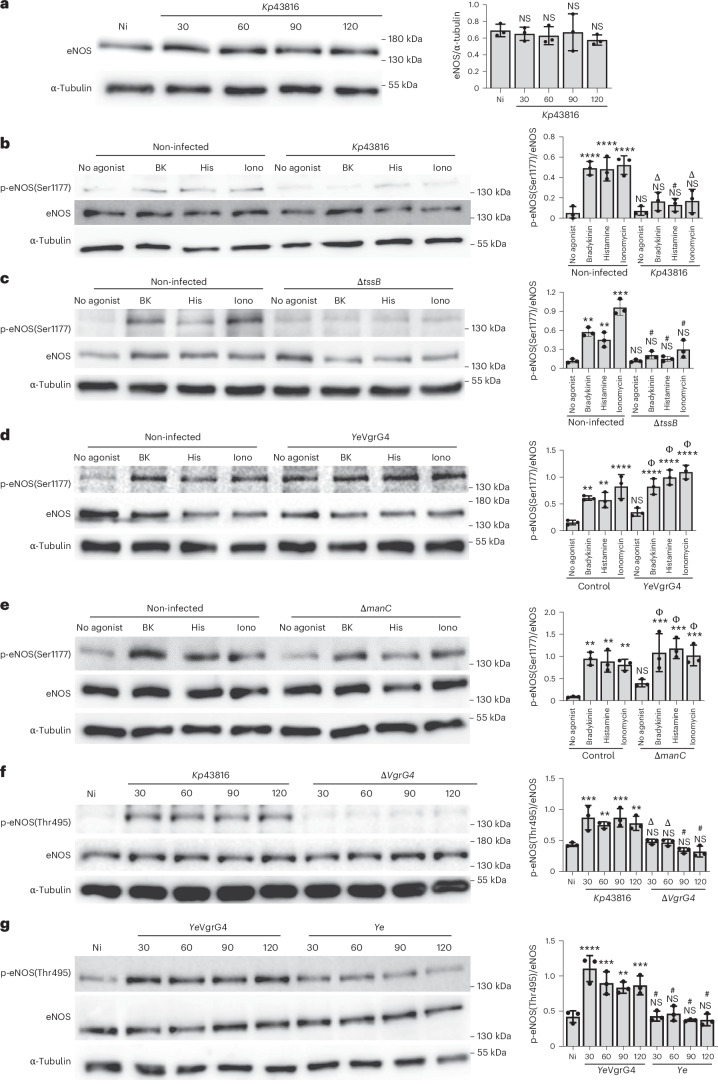
*K.* *pneumoniae* targets eNOS post-translational modifications in a T6SS effector VgrG4-dependent and capsule-dependent manner. **a**, Left, immunoblot analysis of eNOS and α-tubulin levels in infected HULECs for the indicated time points. Right, densitometry analysis of the blots (*n* = 3) representing the ratio of eNOS versus α-tubulin. **b**, Left, immunoblot analysis of phosphorylated eNOS(Ser1177) (p-eNOS(Ser1177)), eNOS and α-tubulin levels in HULECs treated for 5 min with 1 μM bradykinin (BK), histamine (His) or ionomycin (Iono). Where indicated, cells were infected with *Kp*431816 for 120 min before adding the agonists. Right, densitometry analysis of the blots (*n* = 3) representing the ratio of p-eNOS(Ser1177) versus eNOS. **c**, Left, immunoblot analysis of p-eNOS(Ser1177), eNOS and α-tubulin levels in HULECs treated for 2 min with 1 μM bradykinin, histamine or ionomycin. Where indicated, cells were infected with Δ*tssB* for 120 min before adding the agonists. Right, densitometry analysis of the blots (*n* = 3) representing the ratio of p-eNOS(Ser1177) versus eNOS. **d**, Left, immunoblot analysis of p-eNOS(Ser1177), eNOS and α-tubulin levels in HULECs treated for 2 min with 1 μM bradykinin, histamine or ionomycin. Where indicated, cells were infected with *Ye*VgrG4 for 120 min before adding the agonists. Right, densitometry analysis of the blots (*n* = 3) representing the ratio of p-eNOS(Ser1177) versus eNOS. **e**, Left, immunoblot analysis of p-eNOS(Ser1177), eNOS and α-tubulin levels in HULECs treated for 2 min with 1 μM bradykinin, histamine or ionomycin. Where indicated, cells were infected with Δ*manC* for 120 min before adding the agonists. Right, densitometry analysis of blots (*n* = 3) representing the ratio of p-eNOS(Ser1177) versus eNOS. **f**, Left, immunoblot analysis of phosphorylated eNOS(Thr495) (p-eNOS^(^Thr495)), eNOS and α-tubulin levels in HULECs infected with *Kp*43816 and Δ*vgrG4* for the indicated time points. Right, densitometry analysis of the blots (*n* = 3) representing the ratio of p-eNOS(Thr495) versus eNOS. **g**, Left, immunoblot analysis of p-eNOS(Thr495), eNOS and α-tubulin levels in HULECs infected with *Ye* or *Ye*VgrG4 for the indicated time points. Right, densitometry analysis of the blots (*n* = 3) representing the ratio of p-eNOS(Thr495) versus eNOS. Blots are representative of three independent experiments. In all panels, data (mean ± s.d.) were compared against the non-infected (Ni) control using one-way ANOVA and Tukey’s multiple group comparison correction. In all panels, **P < 0.01, ***P < 0.001, ****P < 0.0001. In **b**–**e**, Δ and # indicate **P < 0.01 and ***P < 0.001, respectively, and Φ not significant for comparisons between agonist-treated samples. In **f** and **g**, Δ and # indicate **P < 0.01 and ***P <0.001, respectively, for comparisons between time points of infected samples.

**Extended Data Fig. 4:**
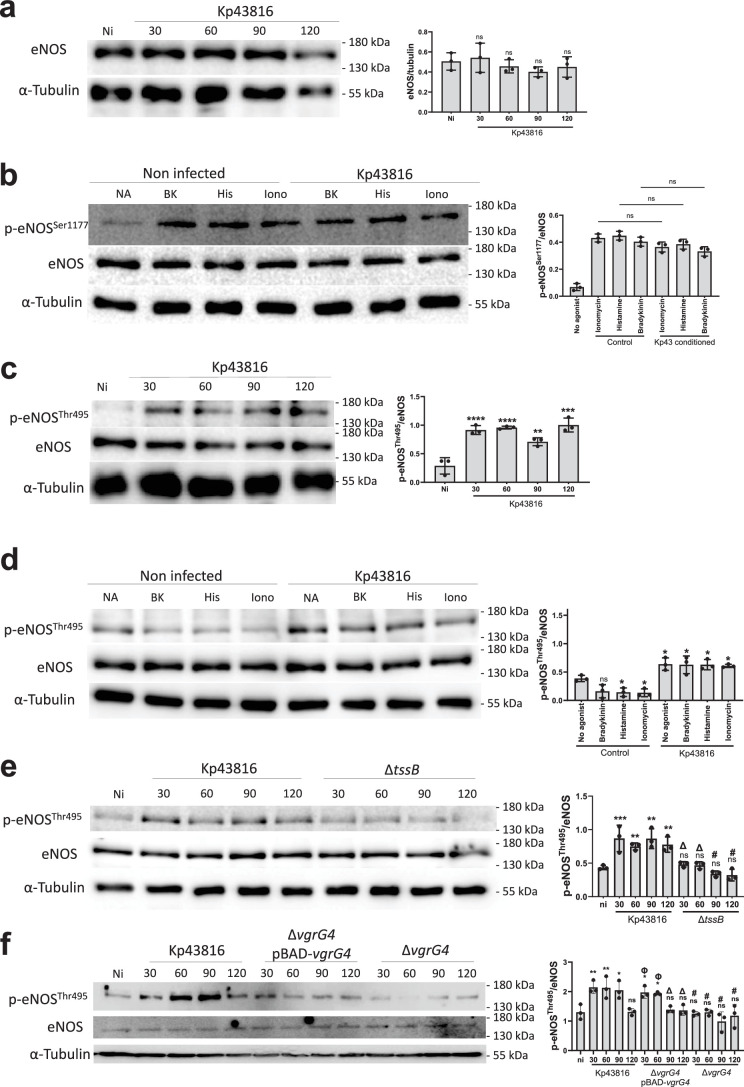
*K. pneumoniae* targets the phosphorylation of eNOS Ser^1177^ and Thr^495^. **a**. Immunoblot analysis of eNOS and tubulin levels in infected HPMEC cells for the indicated time points. Densitometry analysis of blots (n = 3) representing the ratio of eNOS versus tubulin. **b**. Immunoblot analysis of phosphorylated eNOS Ser1177, eNOS and tubulin levels in HULEC cells treated with condition media from control medium containing 1 μM bradykinin (Bk), histamine (His), and ionomycin (Iono), and media in which the agonists were incubated with *Kp*43816 for 5 min, and then bacteria were spun down. Densitometry analysis of blots (n = 3) representing the ratio of phosphorylated eNOS Ser1177 versus eNOS. **c**. Immunoblot analysis of phosphorylated eNOS Thr495 and tubulin levels in infected HPMEC cells for the indicated time points. Densitometry analysis of blots (n = 3) representing the ratio of phosphorylated eNOS Thr495 versus eNOS. **d**. Immunoblot analysis of phosphorylated eNOS Thr495, eNOS and tubulin levels in non-infected and infected HULEC cells treated for 2 min with 1 μM bradykinin (Bk), histamine (His), and ionomycin (Iono). Densitometry analysis of blots (n = 3) representing the ratio of phosphorylated eNOS Thr495 versus eNOS. **e**. Immunoblot analysis of phosphorylated eNOS Thr495, eNOS and tubulin levels in non-infected and infected HULEC cells with strains *Kp*43816 and the T6SS tssB mutant (Δ*tssB*; 43816-Δ*tssB*) for the indicated time points. Densitometry analysis of blots (n = 3) representing the ratio of phosphorylated eNOS Thr495 versus eNOS. **f**. Immunoblot analysis of phosphorylated eNOS Thr495, eNOS and tubulin levels in non-infected and infected HULEC cells with strains *Kp*43816, *vgrG4* mutant (Δ*vgrG4*; 43816-ΔvgrG4), and the complemented mutant for the indicated time points. Densitometry analysis of blots (n = 3) representing the ratio of phosphorylated eNOS Thr495 versus eNOS. Blots are representative of three independent experiments. In all panels, data were compared against the non-infected (Ni) control using one-way ANOVA and Tukey’s multiple group comparison correction. The asterisks indicate p < 0.05 (*), < 0.01 (**). < 0.001 (***), or <0.0001 (****). ns, not significant. In panels **e** and **f**, Φ, not significant; Δ, <0.01 (**), #, <0.001 (***) for comparisons between time points of infected samples with *Kp*43816, and mutants or complemented strain.

Our findings demonstrate that *Klebsiella* blunts the NO pathway, with vasodilation restored through the use of an NO donor. Because *Klebsiella* infection does not impair Ca^2+^ responses, an upstream requirement for eNOS activation^6,7^, we investigated whether *Klebsiella* reduces eNOS expression in a VgrG4-dependent manner. Immunoblot analyses showed that infected HULECs did exhibit altered eNOS protein levels (Fig. 4a). Similar results were obtained in primary human pulmonary microvascular endothelial cells (HPMECs) (Extended Data Fig. 4a). We next examined whether *Klebsiella* targets post-translational modifications that regulate eNOS activity^36,37^. Phosphorylation of Ser1177 activates eNOS^38,39^, whereas phosphorylation of Thr495 in the calmodulin-binding domain inhibits enzyme activity^38,39^. eNOS agonists induce phosphorylation of Ser1177, whereas inhibitory stimuli promote Thr495 phosphorylation^38,39^. Importantly, Thr495 phosphorylation alone is sufficient to inhibit eNOS regardless of the phosphorylation status of the Ser1177 residue^39^. We first investigated whether *Klebsiella* targets Ser1177 phosphorylation. Immunoblot analyses showed that *Kp*43816, unlike the eNOS agonists bradykinin, histamine and ionomycin, did not induce Ser1177 phosphorylation of eNOS (Fig. 4b). Notably, *Kp*43816 inhibited agonist-induced Ser1177 phosphorylation (Fig. 4b), which suggests that *Klebsiella* limits phosphorylation of the activating eNOS site. Control experiments confirmed that pre-incubation of agonists with *Klebsiella* did not impair their intrinsic ability to induce Ser1177 phosphorylation (Extended Data Fig. 4b). To determine whether the T6SS effector VgrG4 mediated this effect, we infected cells with a *tssB* mutant. Inhibition of agonist-induced Ser1177 phosphorylation persisted in these cells (Fig. 4c). However, in cells infected with *Ye*VgrG4, we did not observe any decrease in the agonist-induced Ser1177 phosphorylation (Fig. 4d). This evidence indicates that the T6SS is not responsible for this phenotype. By contrast, cells infected with a *cps* mutant retained agonist-induced Ser1177 phosphorylation (Fig. 4e), which reveals that *Klebsiella*-mediated inhibition of Ser1177 phosphorylation depends on the CPS.

We next questioned whether *Klebsiella* induces phosphorylation of the inhibitory Thr495 site. *Kp*43816 induced Thr495 phosphorylation in both HULECs (Fig. 4f) and HPMECs (Extended Data Fig. 4c). This phosphorylation also occurred in cells treated with eNOS agonists (Extended Data Fig. 4d). However, Thr495 phosphorylation was not observed in cells infected with the *tssB* or *vgrG4* mutant (Fig. 4f and Extended Data Fig. 4e). Complementation of the *vgrG4* mutant restored Thr495 phosphorylation (Extended Data Fig. 4f). Infection with *Ye*VgrG4 also triggered Thr495 phosphorylation (Fig. 4g). Altogether, this evidence establishes that VgrG4 is required to induce phosphorylation of the eNOS inhibitory site Thr495 to blunt eNOS activity.

Collectively, these results demonstrate that *Klebsiella* blunts eNOS activity by inducing phosphorylation of the inhibitory site Thr495 in a T6SS VgrG4-dependent manner and limiting phosphorylation of the activation site Ser1177 in a CPS-dependent manner. Notably, VgrG4-dependent Thr495 phosphorylation alone is sufficient to halt eNOS activity and thereby impairing vasodilation^39–41^.

### CPS limits eNOS(Ser1177) phosphorylation via EGFR–PP2Ac

**Extended Data Fig. 5:**
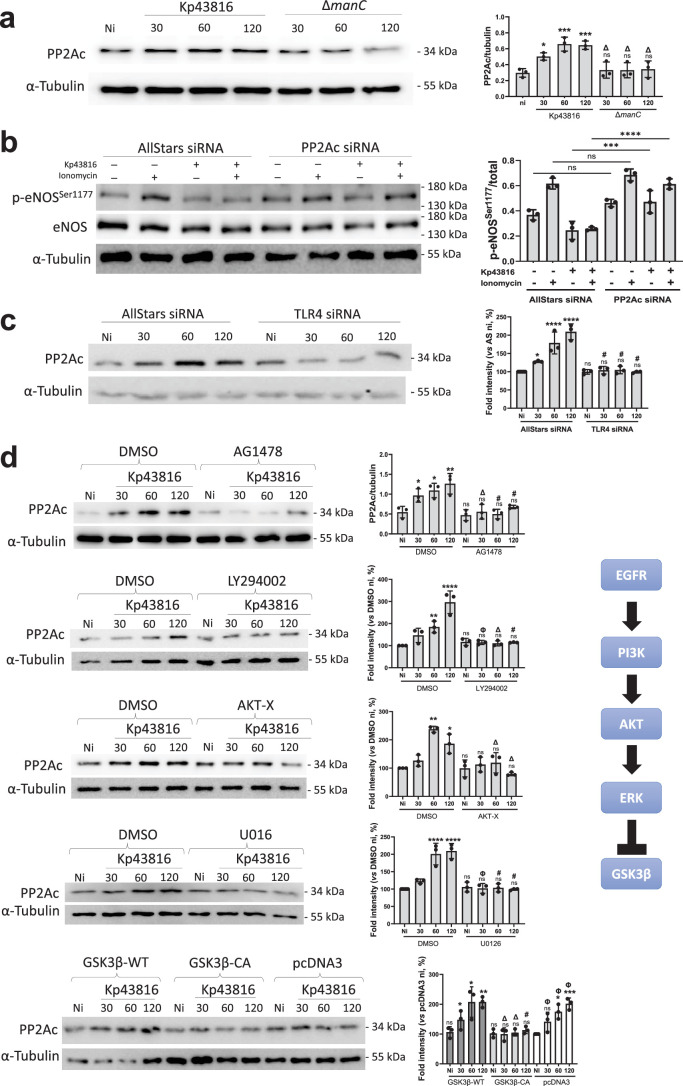
*K. pneumoniae* licences PP2Ac phosphatase to abrogate agonist-induced eNOS Ser^1177^ phosphorylation. **a**. Immunoblot analysis of PP2Ac and tubulin levels in HULEC cells infected with *Kp*43816 and the *cps* mutant (Δ*manC*; 43816-Δ*manC*) for the indicated time points. Densitometry analysis of blots (n = 3) representing the ratio of PP2Ac versus tubulin. **b**. Immunoblot analysis of phosphorylated eNOS Ser1177 in HULEC cells transfected with All Stars siRNA control, or PP2Ac siRNA. Cells were non-infected, treated for 5 min 1 μM ionomycin, infected for 60 min, or infected and treated with ionomycin. Densitometry analysis of blots (n = 3) representing the ratio of phosphorylated eNOS Ser1177 versus eNOS. **c**. Immunoblot analysis of PP2Ac and tubulin levels in HULEC cells transfected with All Stars siRNA control, or TLR4 siRNA infected with Kp43816 for the indicated time points. Densitometry analysis of blots (n = 3) representing the ratio of PP2Ac versus tubulin. **d**. Immunoblot analysis of PP2Ac and tubulin levels in HULEC cells infected with *Kp*43816 for the indicated time points. Cells were pretreated with AG1478 (5 μM), LY294002 (20 μM), AKT-X (30 μM), U0126 (10 μM), or DMSO (vehicle solution) for 2 h before infection where indicated. Where indicated, cells were transfected with plasmids expressing GSK3β either wild-type (GSK3β-WT) or with a S9A mutation that renders the protein constitutively active (GSK3β-CA) or with pcDNA3, empty vector. The signalling pathway EGFR-PI3K-AKT-ERK-GSK3β activated in *K. pneumoniae* infection is depicted to represent the several steps inhibited in this figure. Densitometry analysis of blots (n = 3) representing the ratio of PP2Ac versus tubulin. Blots are representative of three independent experiments. In all panels, data were compared against the non-infected (Ni) control using one-way ANOVA and Tukey’s multiple group comparison correction. The asterisks indicate p,0.05 (*), < 0.01 (**). < 0.001 (***), or <0.0001 (****). ns, not significant. In **a**, **c**, and **d**, Δ and # indicate p < 0.01 (**) and <0.001 (***), respectively, and Φ not significant for comparisons between time points of infected samples, or between transfected cells with pcDNA3 or GSK3β and transfected cells with GSK3β-WT.

**Extended Data Fig. 6:**
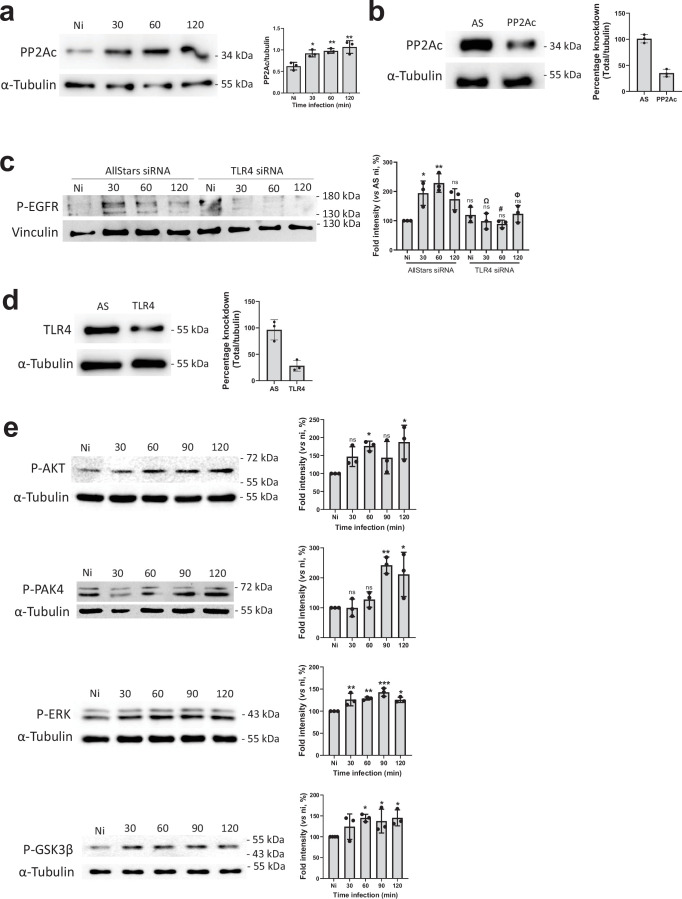
*K. pneumoniae* induces PP2Ac phosphatase to target eNOS Ser^1177^. **a**. Immunoblot analysis of PP2Ac and tubulin levels in infected HPMEC cells for the indicated time points. Densitometry analysis of blots (n = 3) representing the ratio of PP2Ac versus tubulin. **b**. Immunoblot analysis of PP2AC and tubulin levels in cells transfected with All Stars siRNA control, or PP2Ac siRNA. Densitometry analysis of blots (n = 3) representing the percentage of PP2Ac reduction in transfected cells with All Stars siRNA control, normalized to 100%, or PP2Ac siRNA. **c**. Immunoblot analysis of phosphorylated EGFR and vinculin levels in HULEC cells transfected with All Stars siRNA control, or tlr4 siRNA. And infected with Kp43816 for the indicated time points. Densitometry analysis of blots (n = 3) representing the ratio of PP2Ac versus vinculin. **d**. Immunoblot analysis of TLR4 and tubulin Densitometry analysis of blots (n = 3) representing the percentage of TLR4 reduction in transfected cells with All Stars siRNA control, normalized to 100%, **e**. Immunoblot analysis of phosphorylated AKT, PAK4, ERK and GSK3β, and tubulin levels in HULEC cells infected with *Kp*43816 for the indicated time points. Densitometry analysis of blots (n = 3) representing the ratio of phosphorylated protein versus tubulin. Blots are representative of three independent experiments. In all panels, data were compared against the non-infected (Ni) control using one-way ANOVA and Tukey’s multiple group comparison correction. The asterisks indicate p < 0.05 (*), < 0.01 (**). < 0.001 (***) or. ns, not significant. In panel **c**, Φ, not significant; Ω, <0.05 (*), #, <0.001 (***) for comparisons between infected All Stars siRNA control transfected cells and infected tlr4 siRNA transfected cells.

We investigated how *Klebsiella* inhibits Ser1177 phosphorylation in a CPS-dependent manner. Because *Kp*43816 inhibited Ser1177 phosphorylation induced by three different agonists through distinct receptors and mechanisms, we reasoned that *Klebsiella* does not target an upstream eNOS activation pathway. Instead, we proposed that *Klebsiella* activates a cellular phosphatase that limits Ser1177 phosphorylation. This possibility is consistent with reports showing that inhibitors of Ser1177 phosphorylation activate the phosphatase PP2Ac^41,42^. In support of this mechanism, *Kp*43816 increased PP2Ac levels in HULECs (Extended Data Fig. 5a) and HPMECs (Extended Data Fig. 6a). Infection with the *cps* mutant, however, did not increase PP2Ac levels (Extended Data Fig. 5a), which suggests that CPS mediates PP2Ac upregulation. To test whether PP2Ac is required for inhibition of Ser1177 phosphorylation, we reduced PP2Ac levels using short interfering RNA (siRNA) and assessed Ser1177 phosphorylation levels by immunoblotting. The knockdown efficiency of *pp2ac* is shown in Extended Data Fig. 6b. In *pp2ac* knockdown cells, *Kp*43816 did not inhibit ionomycin-induced Ser1177 phosphorylation unlike in cells treated with AllStars control siRNA (Extended Data Fig. 5b). These findings indicate that *Klebsiella* increases PP2Ac levels in a CPS-dependent manner to inhibit Ser1177 phosphorylation.

To decipher how CPS upregulates PP2Ac, we considered whether CPS activates a pathway that controls phosphatase expression. We previously demonstrated that CPS activates a TLR4–EGFR–phosphatidylinositol 3-OH kinase (PI3K)–AKT–PAK4–ERK–GSK3β signalling pathway to upregulate CYLD deubiquitinase in human lung cells^43^. We therefore investigated whether this pathway also regulates PP2Ac in human endothelial cells. In *tlr4* knockdown cells, *Kp*43816 did not phosphorylate EGFR (Extended Data Fig. 6c). *tlr4* knockdown efficiency was higher than 70% (Extended Data Fig. 6d). Immunoblotting confirmed that *Kp*43816 activated phosphorylation of AKT, PAK4 and ERK (Extended Data Fig. 6e). Because GSK3β is constitutively active and inhibited by AKT-mediated phosphorylation of the Ser9 residue^44,45^, we assessed its phosphorylation status and found that *Kp*43814 induced GSK3β phosphorylation, as expected (Extended Data Fig. 6e). *Kp*43816 did not increase PP2Ac levels in *tlr4* knockdown cells (Extended Data Fig. 5c). We used pharmacological inhibitors of EGFR (AG1478), PI3K (LY294002), AKT (AKT-X) and MEK (U0126) to block the pathway at four different levels. In each case, *Kp*43186-induced PP2Ac expression was reduced (Extended Data Fig. 5d). We also examined the role of GSK3β inhibitory phosphorylation by expressing either wild-type GSK3β (GSK3β(WT)) or a constitutive active mutant (GSK3β(CA)) in which the Ser9 residue was changed to alanine in HULECs. Unlike cells transfected with the empty vector pcDNA3 or with GSK3β(WT), cells expressing GSK3β(CA) did not upregulate PP2Ac following *Kp*43816 infection (Extended Data Fig. 5d).

In summary, these findings support a model in which *Klebsiella* CPS activates a TLR4–EGFR–PI3K–AKT–PAK4–ERK–GSK3β signalling pathway to increase phosphatase PP2Ac levels to inhibit eNOS-mediated Ser1177 phosphorylation.

### VgrG4 phosphorylates eNOS(Thr495) via NLRX1–mtROS–PKCβ

**Fig. 5:**
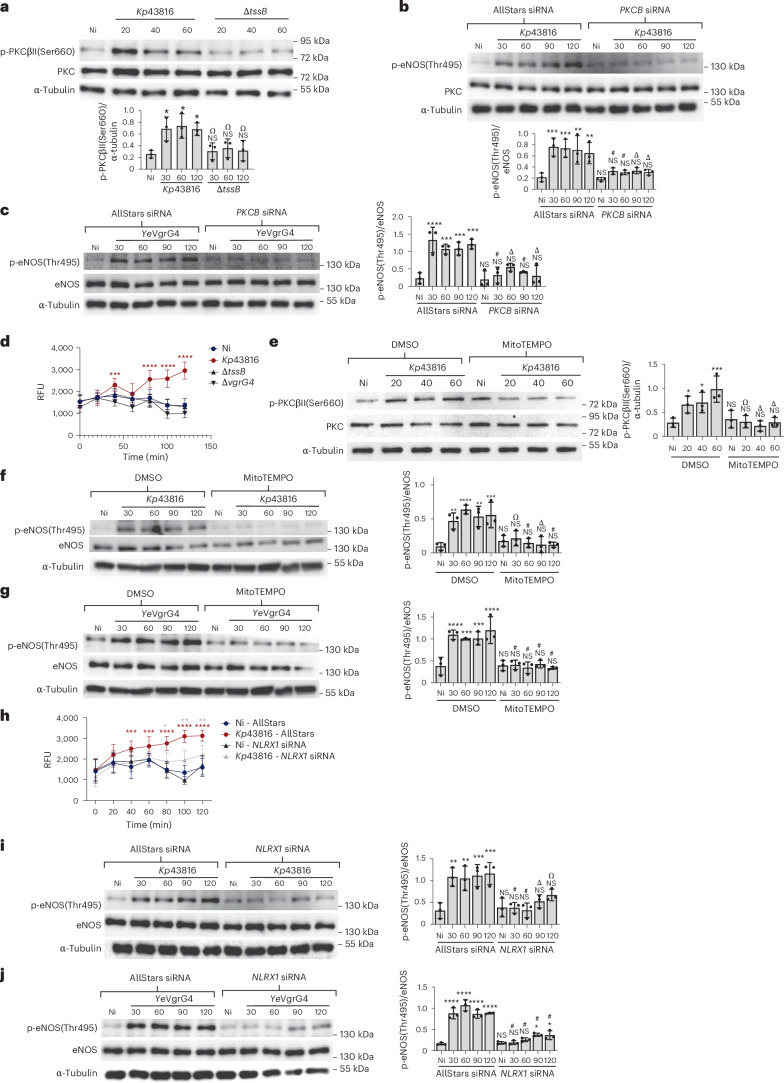
*K.* *pneumoniae* licenses PKCβ kinase to phosphorylate eNOS(Thr495). **a**, Top, immunoblot analysis of phosphorylated PKCβII(Ser660) (p-PKCβII(Ser660)), PKC and α-tubulin levels in HULECs infected with *Kp*43816 or Δ*tssB* for the indicated time points. Bottom, densitometry analysis of the blots (*n* = 3) representing the ratio of p-PKCβII(Ser660) versus α-tubulin. **b**, Top, immunoblot analysis of p-eNOS(Thr495), eNOS and α-tubulin levels in HULECs transfected with AllStars siRNA control or with *PKCB*-targeting siRNA (to deplete PKCβ) and infected with *Kp*43816 for the indicated time points. Bottom, densitometry analysis of the blots (*n* = 3) representing the ratio of p-eNOS(Thr495) versus eNOS. **c**, Left, immunoblot analysis of p-eNOS(Thr495), eNOS and α-tubulin levels in HULECs transfected with AllStars siRNA control or with *PKCB*-targeting siRNA and infected with *Ye*VgrG4 for the indicated time points. Right, densitometry analysis of the blots (*n* = 3) representing the ratio of p-eNOS(Thr495) versus eNOS. **d**, mtROS was quantified as relative fluorescence units (RFU) in HULECs treated with MitoSOX (2 µM, 45 min before infection) and then infected with *Kp*43816 or with Δ*tssB* and Δ*vgrG4* mutants for the indicated time points. **e**, Left, immunoblot analysis of p-PKCβII(Ser660), PKC and α-tubulin levels in HULECs treated with vehicle solution (DMSO) or MitoTEMPO and infected with *Kp*43816 for the indicated time points. Right, densitometry analysis of the blots (*n* = 3) representing the ratio of p-PKCβII(Ser660) versus α-tubulin. **f**, Left, immunoblot analysis of p-eNOS(Thr495), eNOS and α-tubulin levels in HULECs treated with DMSO or MitoTEMPO and infected with *Kp*43816 for the indicated time points. Right, densitometry analysis of blots (*n* = 3) representing the ratio of p-eNOS(Thr495) versus eNOS. **g**, Left, immunoblot analysis of p-eNOS(Thr495), eNOS and α-tubulin levels in HULECs treated with vehicle solution or MitoTEMPO, and infected with *Ye*VgrG4 for the indicated time points. Right, densitometry analysis of the blots (*n* = 3) representing the ratio of p-eNOS(Thr495) versus eNOS. **h**, mtROS was quantified as RFU in HULECs transfected with AllStars siRNA control or with *NLRX1*-targeting siRNA, treated with MitoSOX (2 µM, 45 min before infection) and then infected with *Kp*43816 for the indicated time points. **i**, Left, immunoblot analysis of p-eNOS(Thr495), eNOS and α-tubulin levels in HULECs transfected with AllStars siRNA control or *NLRX1*-targeting siRNA and infected with *Kp*43816 for the indicated time points. Right, densitometry analysis of the blots (*n* = 3) representing the ratio of p-eNOS(Thr495) versus eNOS. **j**, Left, immunoblot analysis of p-eNOS(Thr495), eNOS and α-tubulin levels in HULECs transfected with AllStars siRNA control or *NLRX1*-targeting siRNA and infected with *Ye*VgrG4 for the indicated time points. Right, densitometry analysis of the blots (*n* = 3) representing the ratio of p-eNOS(Thr495) versus eNOS. Blots are representative of three independent experiments. In **d** and **h**, data are the mean ± s.d. of three independent experiments. In all panels for densitometry, data were compared against the non-infected (Ni) control using one-way ANOVA and Tukey’s multiple group comparison correction. In **d** and **h**, data were compared against non-infected control using two-way ANOVA and Dunnet’s multiple group comparison correction. *P < 0.05, **P < 0.01, ***P < 0.001, ****P < 0.0001. In **a**–**c**, **e**–**g**, **i** and **j**, Ω, *P < 0.05; Δ, **P < 0.01; and #, ***P < 0.001 for comparisons between time points of infected samples.

**Extended Data Fig. 7:**
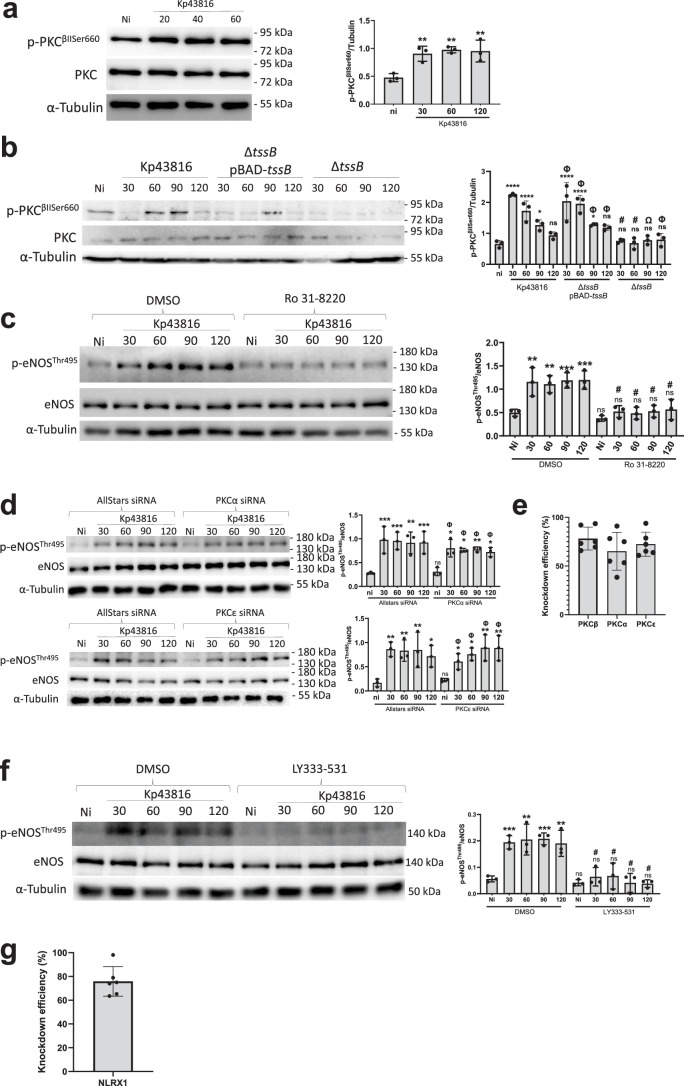
*K. pneumoniae* activates PKCβ kinase to phosphorylate eNOS Thr^495^. **a**. Immunoblot analysis of phosphorylated PKC and tubulin levels in infected HPMEC cells for the indicated time points. Densitometry analysis of blots (n = 3) representing the ratio of phosphorylated PKC versus tubulin. **b**. Immunoblot analysis of phosphorylated PKC and tubulin levels in infected HULEC cells with Kp43816, tssB mutant (ΔtssB; 43816-Δ*tssB*), and the complemented mutant for the indicated time points. Densitometry analysis of blots (n = 3) representing the ratio of phosphorylated PKC versus tubulin. **c**. Immunoblot analysis of phosphorylated eNOS Thr495, eNOS and tubulin levels in infected HULEC cells treated with vehicle solution DMSO and the PKC inhibitor RO 31-8220 for the indicated time points. Densitometry analysis of blots (n = 3) representing the ratio of phosphorylated eNOS Thr495 versus eNOS. **d**. Immunoblot analysis of phosphorylated eNOS Thr495, eNOS and tubulin levels in HULEC cells transfected with All Stars siRNA control, PKCα or PKCε siRNA infected with *Kp*43816 for the indicated time points. Densitometry analysis of blots (n = 3) representing the ratio of phosphorylated eNOS Thr495 versus eNOS. **e**. Efficiency of transfection of siRNA into HULEC cells. Efficiency of transfection presented as percent knockdown after transfection. mRNA levels of the indicated PKC transcripts were accessed 48 h post transfection as fold change against control non silencing agents (AS [AllStars control, non silencing siRNA] after gene normalization. Values are presented as the means ± SD from three independent experiments measured in duplicates. **f**. Immunoblot analysis of phosphorylated eNOS Thr495, eNOS and tubulin levels in infected HULEC cells and treated with vehicle solution DMSO and the PKCβ inhibitor LY333-531 for the indicated time points. Densitometry analysis of blots (n = 3) representing the ratio of phosphorylated eNOS Thr495 versus eNOS. **g**. Efficiency of transfection presented as percent knockdown after transfection. mRNA levels of the NLRX1 transcripts were accessed 48 h post transfection as fold change against control non silencing agents (AS [AllStars control, non silencing siRNA] after gene normalization. Values are presented as the means ± SD from three independent experiments measured in duplicates. Blots are representative of three independent experiments. In all panels, data were compared against the non-infected (Ni) control using one-way ANOVA and Tukey’s multiple group comparison correction. The asterisks indicate p < 0.05 (*), < 0.01 (**). < 0.001 (***) or. ns, not significant. In panels **b**, **c**, **d** and **f**, Φ, not significant; Ω, <0.05 (*), #, <0.001 (***) for comparisons between *Kp*43816 infected samples, and those infected with mutants, complemented strain, treated with drugs, or transfected with PKCα or PKCε siRNA.

Structure–function studies indicate that VgrG4 lacks any kinase activity^26^. To explain VgrG4-induced Thr495 phosphorylation, we speculated that VgrG4 licenses a host kinase involved in the phosphorylation of this eNOS inhibitory site. Several PKC family kinases phosphorylate Thr495 under conditions such as hypoglycaemia and atherosclerosis^39,41,46–48^. We therefore asked whether *Klebsiella* activates PKC. Infection of HULECs and HPMECs with *Kp*43816, but not the *tssB* mutant, induced phosphorylation of the conserved PKC activation site common to all PKC isoforms (Fig. 5a and Extended Data Fig. 7a). Complementation of the *tssB* mutant restored PKC phosphorylation (Extended Data Fig. 7b), which demonstrates that PKC activation depends on the T6SS. To link PKC activation with *Klebsiella*-induced phosphorylation of the inhibitory eNOS(Thr495) site, infections were performed in the presence of the PKC inhibitor Ro31-8220. Under these conditions, Thr495 phosphorylation was abolished (Extended Data Fig. 7c). To identify the PKC isoform responsible, we carried out a siRNA-based screen and assessed Thr495 phosphorylation by immunoblotting. Among the PKCα, PKCβ and PKCε isoforms tested, *Kp*43816-induced Thr495 phosphorylation was only abrogated in cells in which PKCβ was depleted (Fig. 5c and Extended Data Fig. 7d). Control experiments confirmed the reduction in *PKC* transcripts in HULECs (Extended Data Fig. 7e). Thr495 phosphorylation was also absent in infected cells treated with the PKCβ inhibitor LY333-531 (ref. ^49^) (Extended Data Fig. 7f). Similarly, *Ye*VgrG4 did not induce Thr495 phosphorylation in PKCβ-depleted cells (Fig. 5c). Altogether, these results demonstrate that VgrG4 activates PKCβ to phosphorylate Thr495, which abrogates eNOS activity.

ROS is an established signal that activates PKCβ^50^. The fact that VgrG4 induces mtROS in human epithelial cells^24^ suggests that VgrG4-induced mtROS may activate PKCβ in endothelial cells. We first ascertained whether VgrG4 induces mtROS in HULECs by measuring oxidation of the mitochondria-localized fluorescent dye MitoSOX. *Kp*43816 induced mtROS production in HULECs in a T6SS VgrG4-dependent manner because neither the *tssB* nor the *vgrG4* mutant increased the levels of mtROS above non-infected cells (Fig. 5d). To substantiate that VgrG4-induced mtROS activates PKCβ to phosphorylate Thr495, infections were done in the presence of the mtROS scavenger MitoTEMPO. MitoTEMPO prevented *Kp*43816-induced PKC activation (Fig. 5e). Moreover, neither *Kp*43816 (Fig. 5f) nor *Ye*VgrG4 (Fig. 5g) indued Thr495 phosphorylation in MitoTEMPO-treated cells, which demonstrates that mtROS mediates PKCβ activation.

We recently demonstrated that VgrG4-induced mtROS depends on activation of the mitochondria innate immune receptor NLRX1 in human lung epithelial cells^24^. Quantification of MitoSOX oxidation confirmed that *Kp*43816 infection did not increase mtROS levels in cells in which *NLRX1* was knocked down using siRNA (Fig. 5h). This result establishes that NLRX1 also controls *Klebsiella*-induced mtROS in human endothelial cells. *NLRX1* knockdown efficiency is shown in Extended Data Fig. 7g. In *NLRX1* knockdown cells, neither *Kp*43816 (Fig. 5i) nor *Ye*VgrG4 (Fig. 5j) triggered Thr495 phosphorylation, which demonstrated that VgrG4-mediated phosphorylation of Thr495 depends on NLXR1.

Altogether, this evidence supports a model in which VgrG4-induced NLRX1-dependent mtROS activates PKCβ, which leads to phosphorylation of the inhibitor eNOS residue Thr495 to blunt eNOS activity.

### Targeting mtROS and PKCβ restores vasodilation

**Fig. 6:**
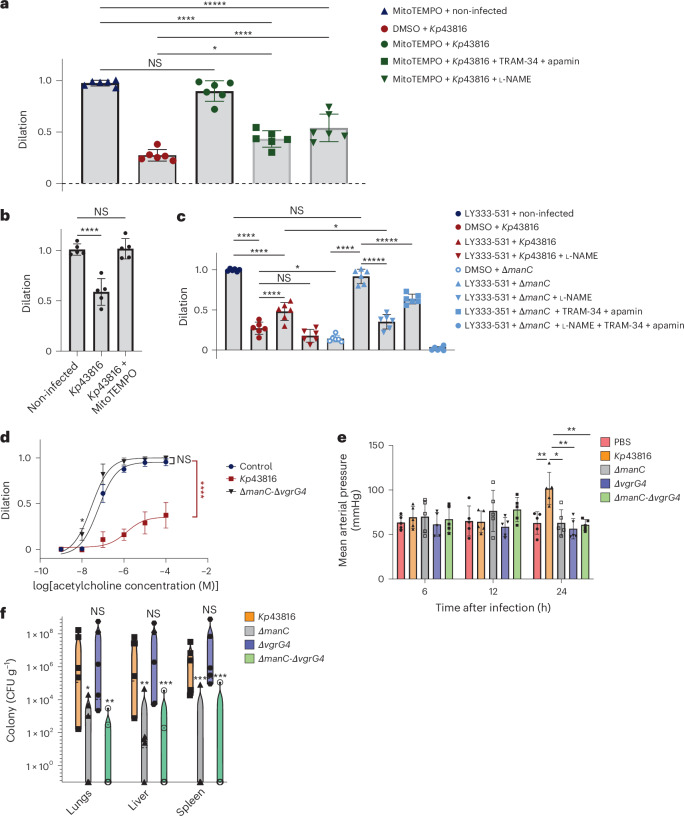
Inhibition of mtROS and PKCβ restores ACh-induced vasodilation in vessels infected with *K.* *pneumoniae*. **a**, ACh-induced peak dilation in vessels treated with vehicle solution (DMSO) or MitoTEMPO and infected with *Kp*43816. Where indicated, infected vessels were also treated with the NO pathway inhibitor L-NAME and with the EDH pathway inhibitors TRAM-34 and apamin. **b**, NS309-induced peak dilation in vessels treated with NS309 in DMSO and infected with *Kp*43816. Where indicated, infected vessels were also treated with MitoTEMPO. **c**, ACh-induced peak dilation in vessels treated with vehicle solution (DMSO) or the PKCβ inhibitor LY333-531 and infected with *Kp*43816 or Δ*manC*. Where indicated, infected vessels were also treated with the NO pathway inhibitor L-NAME and the EDH pathway inhibitors TRAM-34 and apamin. **d**, Dose–response curves of ACh-induced peak dilation in non-infected and infected vessels with *Kp*43816 or the Δ*manC*-Δ*vgrG4* mutant. **e**, Blood pressure, expressed as mmHg, in PBS mock-infected mice and in mice infected with *Kp*43816, Δ*manC*, Δ*vgrG4* or Δ*manC*-Δ*vgrG4* at the indicated times. **f**, Bacterial loads in lung, spleen and liver 24 h after infection in the mice used to measure blood pressure. In **a** and **c**, ACh was used at 1 μM. Peak dilation is shown as the mean ± s.d. from six vessels from three rats (two per rat) per group and analysed using two-way ANOVA and Dunnet’s multiple group comparison correction. In **e**, five mice per group were followed over time in two independent infections and analysed using two-way ANOVA and Dunnet’s multiple group comparison correction. Results of individual mice are displayed, with bars representing the median ± s.d. In **f**, data are shown as violin plots with one mouse per symbol, and significant differences between each of the mutants and the wild-type-infected mice were analysed using two-way ANOVA and Dunnet’s multiple group comparison correction. *P < 0.05, **P < 0.01, ***P < 0.001, ****P < 0.0001.

**Extended Data Fig. 8:**
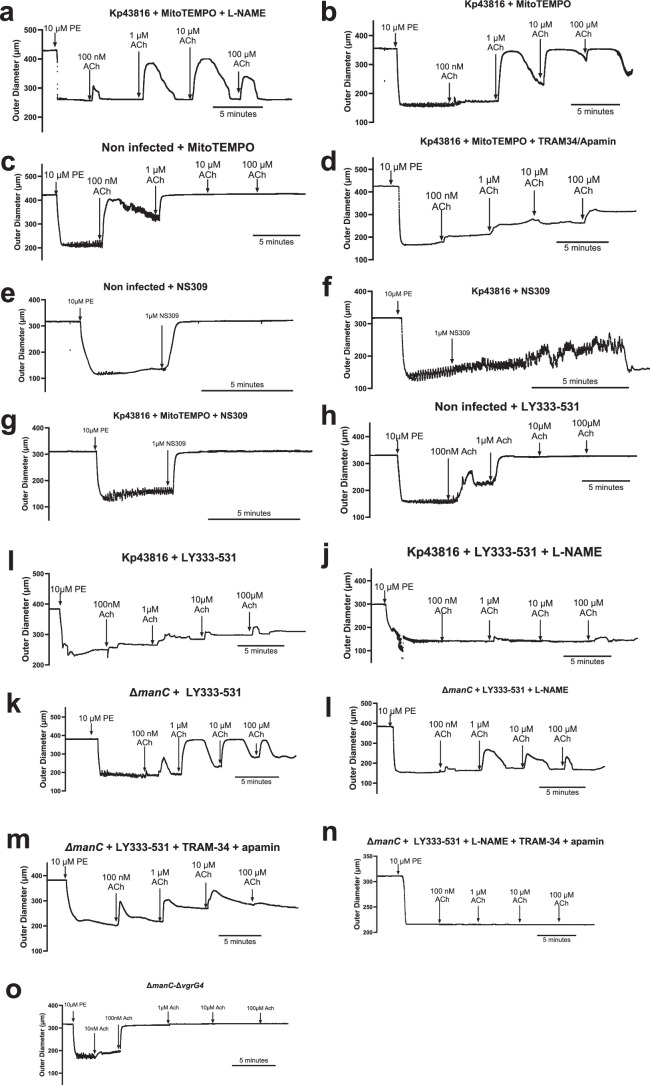
Treatment with MitoTEMPO and PKCβ inhibitor restores ACh-induced vasodilation in *K. pneumoniae*-infected vessels. **a**. Representative trace of the lack of effect of MitoTEMPo on ACh-induced vasodilation in non-infected PE-constricted vessels. **b**. Representative trace of the effect of MitoTEMPO on ACh-induced vasodilation in *Kp*43816-infected PE-constricted vessels. **c**. Representative trace of the effect of MitoTEMPO and L-NAME on ACh-induced vasodilation in *Kp*43816-infected PE-constricted vessels. **d**. Representative trace of the effect of MitoTEMPO, TRAM-34 and apamin on ACh-induced vasodilation in *Kp*43816-infected PE-constricted vessels. **e**. Representative trace of NS309-indcued vasodilation in non-infected PE-constricted vessels. **f**. Representative trace of *Kp*43816-mediated inhibition of NS-309-indcued vasodilation in PE-constricted vessels. **g**. Representative trace of the effect of MitoTEMPO and NS309-induced vasodilation in Kp43816-infected PE-constricted vessels. **h**. Representative trace of the lack of effect of the PKCβ inhibitor LY333-531 on ACh-induced vasodilation in non-infected PE-constricted vessels. **i**. Representative trace of the effect of LY333-531 on ACh-induced vasodilation in *Kp*43816-infected PE-constricted vessels. **j**. Representative trace of the effect of LY333-531 and L-NAME on ACh-induced vasodilation in *Kp*43816-infected PE-constricted vessels. **k**. Representative trace of the effect of LY333-531 on ACh-induced vasodilation in PE-constricted vessels infected with the *cps* mutant (Δ*manC*; 43816-Δ*manC*). **l**. Representative trace of the effect of LY333-531 and L-NAME on ACh-induced vasodilation in PE-constricted vessels infected with the *cps* mutant (Δ*manC*; 43816-Δ*manC*). **m**. Representative trace of the effect of LY333-531, TRAM-34 and apamin on ACh-induced vasodilation in PE-constricted vessels infected with the *cps* mutant (Δ*manC*; 43816-Δ*manC*). **n**. Representative trace of the effect of LY333-531, L-NAME, TRAM-34 and apamin on ACh-induced vasodilation in PE-constricted vessels infected with the *cps* mutant (Δ*manC*; 43816-Δ*manC*). **o**. Representative trace of the lack of effect of infection with the double *cps* and *vgrG4* mutant (Δ*manC*-Δ*vgrG4*; 43816-Δ*manC*-Δ*vgrG4*) on ACh-induced vasodilation in PE-constricted vessels. The traces are representative of six vessels from three rats (two per rat).

On the basis of our cell culture studies and the role of Thr495 phosphorylation in inhibiting eNOS activity^38,39^, we investigated whether inhibition of mtROS and PKCβ restores ACh-induced vasodilation in infected vessels. MitoTEMPO did not affect ACh-induced vasodilation in controls vessels (Fig. 6a and Extended Data Fig. 8a), but restored vasodilation in *Kp*43816-infected vessels to levels comparable to control vessels (Fig. 6a and Extended Data Fig. 8b). Restoration of ACh-induced vasodilation involved both the NO and EDH pathways, as shown using L-NAME, TRAM-34 and apamin to delineate these pathways (Fig. 6a and Extended Data Fig. 8c,d). These findings demonstrate that inhibition of VgrG4-induced mtROS restores NO-mediated and EDH-mediated vasodilation impaired by *K.* *pneumoniae* infection

To investigate how VgrG4-induced mtROS blunts the EDH pathway, we assessed vasodilation induced using NS309, an agonist of SKCa and IKCa channels that drive endothelial hyperpolarization^51^. NS309 induced vasodilation in non-infected vessels (Fig. 6b and Extended Data Fig. 8e), whereas this response was impaired in *Klebsiella*-infected vessels (Fig. 6b and Extended Data Fig. 8f). MitoTEMPO restored NS309-induced vasodilation in infected vessels to levels comparable to non-infected controls (Fig. 6b and Extended Data Fig. 8g). Together with previous evidence showing that *K.* *pneumoniae* does not alter endothelial Ca^2+^ signalling, these findings indicate that mtROS-mediated inhibition of the EDH pathway occurs at, or downstream of, the SKCa–IKCa channel signalling axis.

We next examined the effect of PKCβ inhibition in infected vessels. The PKCβ inhibitor LY333-531 did not affect ACh-induced vasodilation in controls vessels (Fig. 6c and Extended Data Fig. 8h), but significantly increased vasodilation in treated infected vessels (Fig. 6c and Extended Data Fig. 8i). This restored response was mediated by NO signalling because L-NAME abrogated ACh-triggered vasodilation in infected vessels treated with LY333-531 (Fig. 6c and Extended Data Fig. 8j). Because PKCβ inhibition did not fully restore vasodilation to levels of control vessels, we considered whether a CPS-mediated effect on Ser1177 phosphorylation contributed to the observed residual impairment. Consistent with this hypothesis, full restoration of vasodilation occurred in vessels infected with the *cps* mutant and treated with LY333-531 (Fig. 6c and Extended Data Fig. 8k). L-NAME, TRAM-34 and apamin significantly reduced ACh-induced vasodilation (Fig. 6c and Extended Data Fig. 8l,m), whereas their combination abolished ACh-triggered vasodilation (Fig. 6c and Extended Data Fig. 8n). Infection with a double mutant lacking *cps* and *vgrG4* did not blunt ACh-induced vasodilation, and responses were comparable to non-infected vessels (Fig. 6d and Extended Data Fig. 8o). Together, this evidence establishes a synergistic contribution of *Klebsiella*-driven modulation of eNOS phosphorylation at Ser1177 and Thr495 in suppressing vasodilation.

To determine the contribution of the CPS and VgrG4 to vascular physiology in vivo, we measured blood pressure in mice after intraperitoneal infection. Unlike mice infected with *Kp*43816, mice infected with a *cps* mutant, a *vgrG4* mutant or a double *cps* and *vgrG4* mutant did not show increases in blood pressure (Fig. 6e). Plating experiments revealed significantly lower bacterial loads in mice infected with the *cps* mutant or with the double *cps* and *vgrG4* mutant than in mice infected with *Kp*43816 (Fig. 6f), with bacterial loads below detection limit in two mice infected with the *cps* mutant and in three mice infected with the double *cps* and *vgrG4* mutant. These findings indicate that attenuation of CPS-deficient mutants prevents a rigorous assessment of the CPS contribution to blood pressure regulation. By contrast, bacterial loads in the lung, spleen and liver of mice infected with the *vgrG4* mutant were comparable to those infected with the wild-type strain (Fig. 6f), thereby highlighting the role of VgrG4 in triggering hypertension.

## Discussion

**Extended Data Fig. 9:**
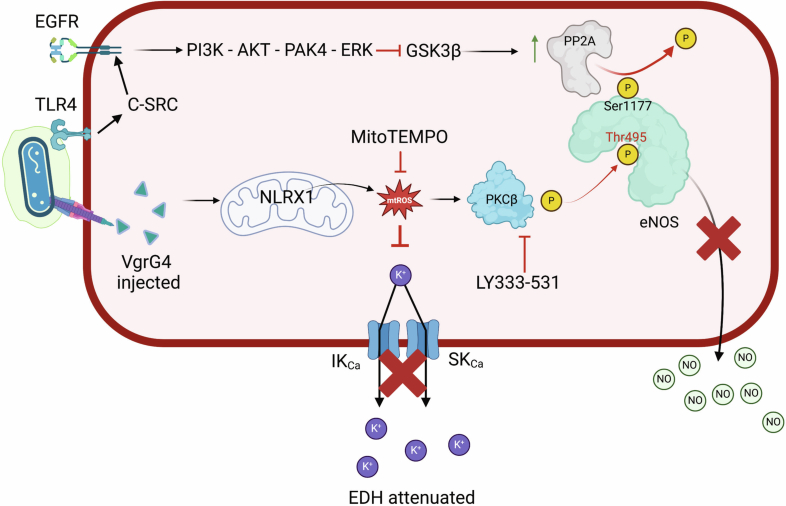
*K. pneumoniae* disrupts vasodilation by targeting eNOS post translational modifications. Working model of the strategies deployed by *Klebsiella* to abrogate ACh-induced vasodilation by affecting the NO and EDH pathways controlling vasodilation in the vessels probed in this work. Klebsiella targets eNOS post translational modifications to blunt the NO pathway. *Klebsiella* activates a TLR4-EGFR-dependent pathway to increase the phosphatase PP2Ac to abrogate the phosphorylation of the eNOS Ser1177 active site whereas the T6SS effector VgrG4 via the innate receptor NLRX1 induces mtROS which licences the PKCβ kinase to phosphorylate eNOS inhibitory site Thr495. VgrG4-induced mtROS attenuates the EDH pathway at the level of the SKCa and IKCa channel axis. Figure created in BioRender; Alphonse, N. https://biorender.com/v7z6dji (2026).

Here we established a research platform, an ex vivo blood vessel model coupled with human primary endothelial cells, to dissect how pathogens affect vascular physiology. We demonstrated that *K.* *pneumoniae* inhibits vasodilation by blunting the NO-dependent pathway and attenuating the EDH pathway (Extended Data Fig. 9). Mechanistically, *Klebsiella* exploits the trans-kingdom T6SS effector VgrG4 to trigger phosphorylation of the eNOS inhibitory site Thr495 via PKCβ in a NLRX1–mtROS-dependent manner (Extended Data Fig. 9). By contrast, the CPS independently inhibits phosphorylation of the eNOS activation site Ser1177 through an EGFR–phosphatase PP2Ac axis (Extended Data Fig. 9). VgrG4-induced mtROS attenuates the EDH pathway at, or downstream of, the SKCa–IKCa signalling axis. These ex vivo findings translated to in vivo physiology, whereby *K.* *pneumoniae* induced hypertension in mice in a VgrG4-dependent manner. Collectively, this work illustrates a new axis through which a human pathogen governs vascular physiology. We propose that the research platform established in this work should help to provide mechanistic insights into the infection biology of other pathogens that cause BSIs.

The research platform described in this work enabled the mechanistic dissection of the *Klebsiella*–vasculature interface, which has so far received little attention, despite the global clinical relevance of *Klebsiella*-caused BSIs. This is a poorly understood niche in the host–pathogen arms race for most bacterial pathogens that cause BSI. Previous research on bacteria–endothelial interactions has relied predominantly on umbilical vein or retinal endothelial cells. Such research has generated valuable observations, but with limited insights into how the findings translate into vascular physiology. Our ex vivo model overcomes this limitation by enabling direct quantification of functional vascular responses to infection. The concordance between findings in human primary endothelial cells and the ex vivo vessel model underscores the robustness of the platform. In vivo confirmation of *K.* *pneumoniae*-induced hypertension further validates its translational relevance.

Inhibition of vasodilation reduces perfusion of infected tissues and impairs immune cell recruitment, which in turn promotes bacterial persistence, tissue damage and the heightened inflammatory cascade characteristic of severe *K.* *pneumoniae* infection. Strikingly, unlike viral and bacterial pathogens that induce endothelial dysfunction by damaging the endothelial barrier^52,53^, *K.* *pneumoniae* achieves this outcome without significant damage to the endothelial layer. Instead, *K.* *pneumoniae* reduces the availability of NO. The pathophysiological importance of this mechanism is underscored by the fact that pharmacological inhibition of NO production with L-NAME increases mortality in *Klebsiella* infection^54^. The central molecular mechanism of this process involves dual-site manipulation of eNOS phosphorylation. Although Thr495 and Ser1177 are well-characterized targets of inhibitors and agonists of vascular vasodilation, until now, there has been minimal evidence to suggest that pathogens commonly exploit these targets. *Klebsiella* achieves this by licensing the kinase PKCβ and the phosphatase PP2Ac, which are both homeostatic regulators of eNOS activity^55^, rather than deploying bacterial effectors that directly engage host targets, as used by *Salmonella*, *Legionella*, *Escherichia* and *Shigella*. This finding extends a theme emerging from the research of our group, whereby *Klebsiella* exploits host enzymes responsible for restoring cellular homeostasis, including the deubiquitinase CYLD, the phosphatase MKP-1, the deSUMOylase SENP2 and the deneddylase CSN5 (refs. ^43,56–61^), to manipulate post-translational modifications to alter cell biology and promote infection.

Another finding is that T6SS activity controls vascular physiology, which substantially expands the capabilities of this secretion system. Although the T6SS is not a major determinant of *Klebsiella* bloodstream fitness in the mouse model^62^, our results demonstrated a direct physiological consequence of T6SS activity beyond interbacterial competition. This new anti-host function adds to established roles of *Klebsiella* T6SS in blocking NF-κB signalling through mitochondrial alteration^24^, in governing the landscape of lung myeloid cells and their interactions with *Klebsiella* to promote lung infection^25^, in facilitating gut colonization^63,64^ and in antimicrobial defence against bacteria and fungi^26^. VgrG4 mediates these anti-host activities; however, the significant effector diversity within *K.* *pneumoniae* T6SS^26,65^ strongly suggests that there are additional effectors with anti-host functions, including ones affecting vascular physiology, and these remain to be identified.

The VgrG4 exerted inhibition of vasodilation by targeting eNOS Thr495 depends on NLRX1-controlled mtROS. *Klebsiella* similarly exploits NLRX1-induced mtROS to inhibit NF-κB signalling with a concomitant reduction in inflammation^24^. There are different reports on the role of NLRX1 in infection, whereby it can act as a positive or negative regulator depending on the infection context^66–69^. There is also conflicting data on the role of NLRX1 on cell death^70–73^. Our work unequivocally places NLRX1 at the fulcrum of *Klebsiella* anti-host strategies. Moreover, our work implicates NLRX1 in the regulation of vascular physiology, laying the groundwork for future investigations into its contribution to diseases characterized by endothelial dysfunction that results from deficiencies in NO bioavailability. Whereas PKCβ inhibition alone restores the NO pathway in infected vessels, recovery of the EDH pathway required mtROS suppression, which identifies mtROS as the master upstream signal that governs *Klebsiella*-mediated inhibition of both vasodilatory axes. The mechanism by which VgrG4-indued mtROS impairs the EDH pathway remains an open question. Our data suggest that the effect of mtROS occurs at the level of, or downstream of, SKCa and IKCa channel activation. The observations that *K.* *pneumoniae* does not alter intracellular Ca^2+^ signalling and that vasodilatory responses to the SKCa–IKCa agonist NS309 are impaired support the idea that mtROS may reduce channel activity by lowering their open probability in response to Ca^2+^, which then limits endothelial hyperpolarization. It is also possible that mtROS interferes with the transmission of hyperpolarizing signals through myoendothelial gap junctions. Future efforts are warranted to investigate the effect of VgrG4-induced mtROS on the SKCa–IKCa signalling axis.

Although VgrG4 is necessary and sufficient to inhibit vasodilation, the CPS independently contributed by suppressing Ser1177 phosphorylation via EGFR–PP2Ac signalling. The synergistic effect of both actions is marked by the fact that a double mutant lacking CPS and VgrG4 did not impair ACh-induced vasodilation. The work of our laboratory and others have established that *Klebsiella* CPS is a bona fide immune evasin^23^. Beyond limiting innate immune recognition^74^, the CPS activates EGFR-dependent signalling to dampen NF-κB activation through the inhibition of K63 ubiquitination of TRAF6 (refs. ^43,56^) and restricts two other post-translational modifications, SUMOylation and NEDDylation, through the activation of this EGFR-dependent pathway^57^. This evidence supports the notion that *Klebsiella* controls the post-translational modifications of host proteins via this EGFR-governed pathway.

Finally, this work also offers translational promise. The phenotypic overlap between *Klebsiella*-induced endothelial dysfunction and cardiovascular disease opens a compelling opportunity to repurpose approved cardiovascular therapeutics as adjunctive or host-directed treatments for *K.* *pneumoniae* infection. Host-directed therapy is gaining traction as a strategy to combat antimicrobial resistance^75^, and leveraging drugs with established safety profiles offers a viable fast-track route from preclinical discovery to clinical evaluation. Future studies shall confirm whether this is the case.

## Methods

### Ethics statement

The experiments involving mice were approved by the Queen’s University Belfast’s Ethics Committee and were conducted in accordance with the UK Home Office regulations (project licence PPL2910) issued by the UK Home Office. The infection protocol adhered to the ARRIVE and NC3Rs guidelines.

### Mice

All experiments were carried out at the Queen’s University Biological Services Unit and involved 8–9-week-old mice of both sexes. C57BL/6 mice were purchased from Charles River Laboratories and placed in sterile conditions after their arrival and for least 1 week before the start of experiments for acclimation. The animals were supplied with food and water ad libitum and were placed in individually ventilated cages when moved to the Biosafety Level 2 laboratory in which the infection experiments were performed. Male and female animals were randomized for interventions, but the researchers who processed the samples and analysed the data were aware of which intervention group corresponded to which cohort of animals.

### Bacterial strains and growth conditions

Details of the strains and bacterial mutants used are provided in Supplementary Table 1. *K.* *pneumoniae* strains and mutants were cultured over 16–18 h in 5 ml LB medium and refreshed the next day by the addition of 0.5 ml of the overnight culture into 4.5 ml fresh medium. Bacteria were collected at mid-exponential phase (2,500*g*, 20 min) and adjusted to an optical density of 1.0 at 600 nm (OD_600_) in PBS (5 × 10^8^ CFU ml^−1^). *Y.* *enterocolitica* strains were grown in 5 ml LB overnight and refreshed in tryptic soy broth (TSB) medium containing 20 mM MgCl_2_ and 20 mM sodium oxalate for 3.5 h at 21 °C followed by 30 min at 37 °C to activate *Yersinia* T3SS. Bacteria were recovered by centrifugation and adjusted to an OD_600_ of 1.0 in PBS (5 × 10^8^ CFU ml^−1^).

### Ex vivo blood vessel and infection

Male Sprague Dawley rats between the age of 6 and 12 weeks were culled using a schedule 1 method, and their mesenteric arcade was isolated and placed into ice-cold PSS (118 mM NaCl, 4.7 mM KCl, 20 mM HEPES, 5 mM NaHCO_3_, 1.2 mM MgCl_2_, 1.2 mM KH_2_PO_4_, 2 mM CaCl_2_ and 5 mM glucose). The mesenteric arcade was pinned onto a sylgard-coated dish using insect pins. Using vannas scissors and microdissection forceps, second and third order mesenteric arteries were separated from the adipose tissue and placed into low-calcium PSS (118 mM NaCl, 4.7 mM KCl, 20 mM HEPES, 5 mM NaHCO_3_, 1.2 mM MgCl_2_, 1.2 mM KH_2_PO_4_, 100 μM CaCl_2_ and 5 mM glucose) and stored at 4 °C. Vessels over 250 μm, under no pressure, were discarded. Each vessel was cannulated using pipettes pulled with a Stutter instrument P-97, using thin-walled borosilicate glass capillaries with an outer diameter of 1.5 mm. Vessels were tied down on the cannula using two nylon size 8/0 sutures half knotted on each end in a DMT Culture myograph 204 chamber. The myograph chamber contained PSS and the vessel was perfused with PSS. The chamber and the PSS were kept at 37 °C.

After cannulation, vessels were allowed to equilibrate for 20 min at an intraluminal pressure of 70 mmHg. Subsequently, 10 μM PE was added into the chamber to induce sustained constriction, which was typically achieved within 2–3 min. As indicated in the traces, ACh was added to the chamber in a step-wise manner (concentrations from 1 nM to 100 μM). When the vessels were infected, bacteria were flowed into the vessel and then the flow was stopped. Vessels were infected for 1 h with *K.* *pneumoniae* strains or for 30 min with *Y.* *enterocolitica* before the addition of PE and ACh. The outer diameter of the vessel was imaged and recorded using an infrared microscope (DMT). The traces were reproduced using DMT Myoview 5 software.

NO-pathway-dependent dilation was inhibited with 100 μM L-NAME, whereas EDH-pathway-controlled dilation was inhibited with 1 μM TRAM-34 and 100 nM apamin. Inhibitors were added to the chamber 30 min before infection and maintained during the duration of the experiment. To ascertain smooth muscle cell reactivity to NO, 5 min after addition of 10 μM PE, 100 μM SNP was added to the chamber. To determine the effect of infection on the EDH pathway elicited by the NS-309 agonist of SKCa and IKCa channels, 5 min after addition of 10 μM PE, 1 μM NS309 was added to the chamber. In the experiments testing the effect of inhibiting PKCβ and mtROS, vessels were incubated with 100 nM LY333-531 and 10 μM MitoTEMPO, respectively, 1 h before infection and maintained during the duration of the experiment. In the experiments using ciprofloxacin to kill extracellular and intracellular bacteria, after 1 h of infection, vessels were perfused with ciprofloxacin (100 µg ml^−1^), and the antibiotic was also added to the chamber. After 1 h, 10 μM PE was added into the chamber to induce sustained constriction followed by 1 μM ACh 5 min later.

To damage the endothelium, after cannulation and equilibration, the vessel lumen was perfused with a stream of air for 30 s, followed by flushing with PSS to remove residual air. This enables controlled endothelial disruption while leaving the underlying smooth muscle layer intact^76^. Successful denudation was evidenced by the loss of ACh-induced relaxation in pre-contracted vessels.

All myography experiments were performed using two vessels per rat. Vessels from three rats were tested unless stated otherwise.

### Calcium signalling in endothelial cells

To quantify intracellular Ca^2+^ signals in cultured endothelial cells in response to agonists and infection, we used Fura2-QBT assays (Molecular Devices). HULECs were seeded in 96-well black-walled, clear-bottom plates at 20,000 cells per well 24 h before the experiment. To load cells with the dye Fura2QBT, wells were washed extensively with HBSS to remove cell medium and loaded with 200 μl Fura2QBT in HBSS. Cells were incubated for 1 h at 37 °C before the addition of drugs. Drugs were prepared in a 96-well V-bottom master plate in HBSS at 4× the final concentration. The plates were placed in a temperature-controlled Flexstation 3 system at 37 °C, which included custom manufactured tips (Molecular Devices) that were used to pipette 50 μl of the drugs at predetermined settings (as per the manufacturer’s guidance). To assess the effect of infection, cells were infected at a multiplicity of infection of 100 bacteria per cell in HBSS after loading the cells with Fura2QBT. After 2 h, the plates were placed in the Flexstation 3 system for the addition of the drugs as described above.

Intracellular Ca^2+^ levels were assessed for 30 s before and for 5 min after the addition of the drugs. Excitation was performed at 340/380 nm, with emission measurements recorded at 510 nm. All experiments were performed with six technical replicates, in three independent occasions.

### Endothelial cell viability

To assess cell viability, we adapted a previously published protocol^77^. HULECs were seeded in a 96-well plate to a density of 40,000 cells per well 24 h before infection. Cells were infected at a multiplicity of infection of 100 to 1 in a final volume of 190 μl antibiotic-free DMEM tissue culture medium supplemented with 10% FCS. Synchronization of infection was performed by centrifugation (200*g* for 5 min). After 120 min, cells were washed once with PBS, and 180 μl fresh medium containing gentamycin (100 μg ml^−1^) was added to the wells to kill extracellular bacteria. Medium containing gentamycin was kept until the end of the experiment. At the indicated time points, cells were washed twice with PBS and incubated with 100 μl freshly prepared neutral red medium (final concentration of 40 μg ml^−1^ (w/v) neutral red (Sigma-Aldrich) in tissue culture medium) for 3 h. Cells were washed twice with PBS, and neutral red taken up by the cells was released by incubation of the cells with 150 μl destaining solution (50% ethanol 96%; 49% deionized water, 1% glacial acetic acid) at room temperature for 15 min with agitation. The released neutral red was quantified by determining the OD_540_ in a plate reader (POLARstar Omega). Neutral red crystallizes in acidic vacuoles in the cells. Dead cells are unable to maintain acidic compartments and hence do not retain neutral red. Next, 2 mM H_2_O_2_ solution was added to the cells to induce 100% cell death. Experiments were carried out in triplicate on three independent occasions.

### Ex vivo measurement of Ca^2+^ responses by the endothelium

Small rat mesenteric resistance arteries (second and third order) were cut open longitudinally and pinned out using tungsten wire, and the endothelium was exposed using microdissection scissors. Vessels were loaded with Cal520-AM (5 µM with 0.02% Pluronic F-127 in PSS at 37 °C for 30 min). After incubation with the dye, the chamber was washed with PSS to remove excess dye. A custom-made chamber was placed on an upright fluorescence microscope and the endothelium was imaged with a ×16 (0.8 NA) water-immersion objective lens at 10 Hz (488 nm excitation wavelength). All images were captured using a Photometrics Evolve 13 EMCCD camera (1,024 × 1,024), and data were recorded using μManager (v.2) software.

Endothelium Ca^2+^ responses were measured before and after the addition of ACh in step-wise increments (3 nM, 100 nM and 3 μM) in control vessels. After completing the recording session with the last concentration of ACh, the vessel was thoroughly washed and then mock-infected or infected with 10^6^ CFU ml^−1^ *Kp*43816 for 1 h before the same concentrations of ACh were added. At the end of the experiment, 4-DAMP (a muscarinic M_3_ receptor antagonist) was added to demonstrate that the observed ACh-induced Ca^2+^ signalling depended on the activation of M_3_ receptors. All results for Ca^2+^ signalling experiments for mock-infected and infected vessels were normalized to their respective vessel control values. Data were plotted using a published algorithm^78^.

In these experiments, a total of number of Ca^2+^ events evoked by ACh were probed.

### Construction of *vgrG4* mutants

Primers for construction of the mutants (Supplementary Table 2) were designed on the basis of the whole-genome sequence of *K.* *pneumoniae* ATCC43816 (GenBank accession no. CP009208.1). The primer pairs *vgrG4* up and *vgrG4* down were used in separate PCR reactions using Q5 DNA Polymerase (New England Biolabs) to amplify 730 bp and 1,027 bp fragments flanking the *vgrG4* gene. BamHI restriction sites internal to these flanking regions were incorporated at the end of each amplicon. Purified *vgrG4* up and down fragments were then polymerized and amplified as a single PCR amplicon using the primers vgrG4_UPFWD and vgrG4_DWNRVS. The 1.7 kb PCR amplicon was gel-purified and cloned by homologous alignment cloning^79^ into EcoRI-digested Antarctic Phosphatase (New England Biolabs)-treated and gel-purified pFOK suicide vector^80^ to generate pFOKVgrG4. This plasmid was transformed by heat shock into *Escherichia* *coli* JKe201 (ref. ^81^), and transformants were selected on LB agar plates containing 100 μM DAP (required for growth of JKe201) and kanamycin (50 μg ml^−1^). pFOKVgrG4 was mobilized to *K.* *pneumoniae* ATCC43816 by conjugation as previously described^80^, and co-integrants were selected on LB plates containing kanamycin (50 μg ml^−1^). At least three co-integrant clones were combined and grown for 4 h at 37 °C in 2 ml of LB. Bacteria were then streaked on freshly prepared LB no-salt agar plates containing 20% sucrose and 0.5 μg ml^−1^ anhydrous tetracycline. Plates were incubated at 28 °C protected from light for at least 24 h. Candidate mutant clones were confirmed by PCR using *vgrG4* SCREEN primer pairs. The confirmed mutant was named 43816-Δ*vgrG4*.

To construct a double mutant lacking CPS and VgrG4, the Km cassette from strain 43Δ*manCKm* was removed by Flp-mediated recombination using the pFLP2Tp plasmid as previously described^82^. The generated mutant was named 43816-Δ*manC*. pFOKVgrG4 was mobilized into 43816-Δ*manC* to obtain the double mutant 43816-Δ*manC*-Δ*vgrG4*.

### Complementation of mutants

Primers for complementation of *vgrG4* and *tssB* mutants were designed on the basis of the *K.* *pneumoniae* ATCC43816 genome sequence (GenBank accession no. CP009208.1). The primer pairs (Supplementary Table 2) pBAD30Cm-vgrG4_F and pBAD30Cm-vgrG4_R or pBAD30Cm-tssB_F and pBAD30Cm-tssB_R were used to amplify the target gene using Q5 High-Fidelity DNA Polymerase (New England Biolabs) and subsequently gel purified. The PCR fragment was cloned into pGEM-T Easy (Promega) and transformed into *E*. *coli* TOP10. After XbaI digestion of the plasmids, the purified fragments were cloned by homologous alignment cloning^79^ into XbaI-digested Antarctic Phosphatase (New England Biolabs)-treated pBAD30Cm vector to generate pBAD30Cm_vgrG4 and pBAD30Cm_tssB. Plasmids were transformed into *E.* *coli* GT115 and subsequently transformed into DAP auxotrophic *E.* *coli* JKe201 (ref. ^81^) and mobilized into *K.* *pneumoniae* by conjugation. *Klebsiella* strains harbouring the vectors were selected in LB agar supplemented with chloramphenicol (30 µg ml^−1^) at 37 °C and confirmed by PCR. The *Ara* promoter of the pBAD plasmid was induced with arabinose (0.2%) for 30 min in LB before collection of bacteria.

### Assessment of bacterial growth

**Extended Data Fig. 10:**
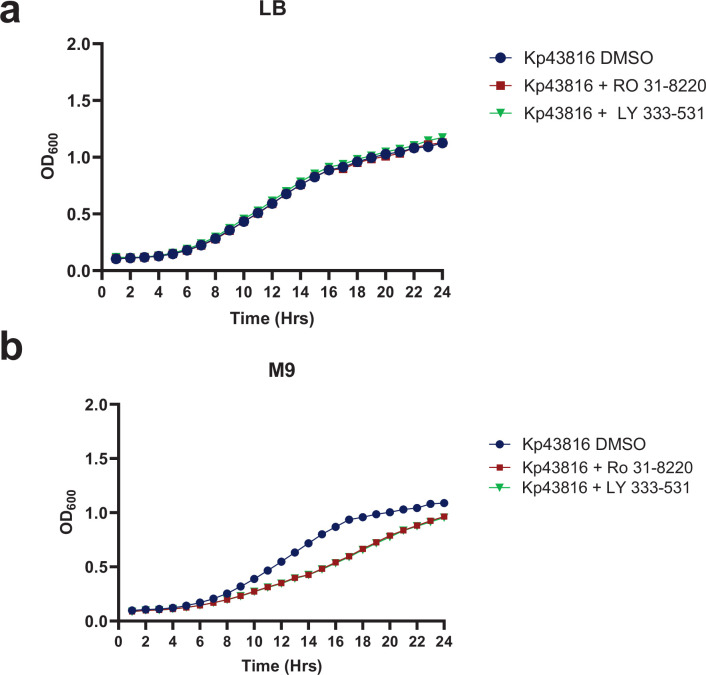
Effect of inhibitors on *K. pneumoniae* growth. **a**. Growth of *Kp*43186 in LB in the presence of vehicle solution DMSO, and the inhibitors Ro 31-8220 and LY333-531. **b**. Growth of *Kp*43186 in M9 minimal medium in the presence of vehicle solution DMSO, and the inhibitors Ro 31-8220 and LY333-531. In a and b, results are shown as mean ± SD from four independent experiments.

To test the effect of inhibitors on the growth of *Klebsiella*, 5 μl from an overnight culture in a 96-well plate was diluted in 250 μl LB or M9 minimal medium (5× M9 minimal salts (Sigma-Aldrich) supplemented with 2% glucose, 3 mM thiamine and 2 mM MgSO_4_) and incubated at 37 °C with continuous, normal shaking in a Bioscreen C Automated Microbial Growth Analyzer (MTX Lab Systems). Absorbance at OD_600_ was measured and recorded every 20 min. Results, reported in Extended Data Fig. 10, demonstrate that the inhibitors analysed did not affect the growth of *Klebsiella*.

### Cell culture

Immortalized the human lung microvascular endothelial cell line HULEC-5a (American Type Culture Collection) was grown in glutamine-free MCDB131 medium (ThermoFisher Scientific) supplemented with 10 mM glutamine, 10 mM HEPES (Sigma), 1 µg ml^−1^ hydrocortisone, 10 ng ml^−1^ human epidermal growth factor and 10% FBS. Unless otherwise indicated, cells were seeded at 125,000 per well in a 12-well plate without antibiotic 24 h before infection. Cells were used between passages 3 and 10.

Primary HPMECs (Innoprot) were grown in endothelial cell medium supplemented with 10% FBS and endothelial cell growth supplement (Innoprot).

HULECs and HPMECs were infected at a multiplicity of infection of 100 bacteria per cell. Cells were routinely tested for *Mycoplasma* contamination.

### Transfection and knockdown efficiency

For transfections, cells were seeded at 60,000 cells per well in a 12-well plat, 48 h before infection. siRNA transfection was performed using Lipofectamine 2000 (Invitrogen) as per the manufacturer’s instructions (3 μl per 100 μl Optimem) with 20 nM siRNA or negative control siRNA (AllStars, Qiagen) to a final volume of 1 ml per well. Knockdown efficiency was confirmed by immunoblotting or by qPCR analysis of duplicate samples from three independent transfections by normalizing to the human glyceraldehyde 3-phosphate dehydrogenase (*GAPDH*) gene and comparing gene expression in the knockdown sample with the AllStars siRNA control. Primers used are listed in Supplementary Table 2.

### Immunoblotting

HULECs and HPMECs were seeded in 12-well plates at a density of 125,000 and 80,000 cells, respectively, 24 h before infection. Infections for western blot analysis were performed with *K.* *pneumoniae* or *Y.* *enterocolitica* for various time points as indicated in the figure legends. When inhibitors or siRNA were used, the details are provided in the figure legends. Cells were then washed in 1 ml ice-cold PBS and lysed in 80 μl 2× SDS sample buffer (1× SDS sample buffer, 62.5 mM Tris-HCl pH 6.8, 2% w/v SDS, 10% glycerol, 50 mM DTT and 0.01% w/v bromophenol blue). Cell lysates were sonicated for 10 s at 10% amplitude with a Branson 450 digital sonicator and were then stored at –20 °C until use. Samples were boiled at 95 °C for 5 min and centrifuged at 12,000*g* for 1 min. Next, 25% (20 µl) of the lysates was resolved by standard 10% SDS–PAGE and electroblotted onto nitrocellulose membranes using semi-dry Trans-Blot (Bio-Rad). Membranes were blocked with 3% BSA (w/v) in TBST for 1 h before being incubated overnight at 4 °C with primary antibodies. The following antibodies were used: anti-eNOS (anti-rabbit, 1:1,000; 9572, Cell Signalling); anti-phospho-eNOS Thr495 (anti-rabbit, 1:1,000; 9574, Cell Signalling); anti-phospho-eNOS(Ser1177) (anti-rabbit, 1:1,000; 9571, Cell Signalling); anti-phospho-PKCβII(Ser660) (anti-rabbit, 1:1,000; 9371, Cell Signalling); anti-PKC (anti-mouse, 1:500; sc-17769, Santa Cruz); anti-PP2A subunit C (anti-mouse, 1:1,000; 610556, BD Transduction Laboratories); anti-phospho-AKT1/2/3 (Ser 473) (anti-rabbit, 1:1,000; sc-33437, Santa Cruz); anti-phospho-ERK (anti-rabbit, 1:1,000; 9101, Cell Signalling); anti-phospho-EGFR^Tyr1068^ (anti-rabbit, 1:1,000; 3777, Cell Signalling); anti-TLR4 (anti-mouse, 1:1,000; sc-293072, Santa Cruz); anti-phospho-PAK4^Ser474^ (anti-rabbit, 1:1,000; 3241, Cell Signalling); anti-phospho-GSK3β^Ser9^ (anti-rabbit, 1:1,000; 5558, Cell Signalling); and vinculin (1:3,000, Sigma-Aldrich, V4505, RRID:AB_477617). Immunoreactive bands were visualized by incubation with horseradish-peroxidase-conjugated goat anti-rabbit immunoglobulins (1:5,000, Bio-Rad, 170-6515) or goat anti-mouse immunoglobulins (1:5,000, Bio-Rad, 170-6516). Bands were detected using chemiluminescence reagents in a G:BOX Chemi XRQ chemiluminescence imager (Syngene).

To detect multiple proteins, membranes were reprobed after stripping off previously used antibodies using a pH 2.2 glycine-HCl–SDS buffer. To ensure that equal amounts of proteins were loaded, blots were reprobed with mouse anti-human tubulin (1:3,000, Sigma T5168).

### Densitometry analysis

Images of the blots were exported from GeneSys software (Syngene) and analysed in Image studio lite (Licor) to obtain signal intensities of the bands. When analysing phosphorylated protein, densitometric analysis was performed by dividing the phosphorylated protein signal intensity over the total protein signal intensity (phospho/total). In cases when total protein was observed, the total protein signal intensity was divided by the signal intensity of the loading control (tubulin or vinculin).

### mtROS detection

HULECs were seeded in a 96-well plate (black, clear bottom) at 15,000 cells per well and incubated with 2 µM MitoSOX red for 45 min in antibiotic-free Hanks’ balanced salt solution (HBSS). Cells were then gently washed three times with prewarmed PBS and infected in fresh antibiotic-free HBSS with *K.* *pneumoniae* as described in ‘Calcium signalling in endothelial cells’. Fluorescence was measured over 2 h (readings taken every 20 min) at excitation and emission of 544 nm and 590 nm, respectively, in a POLARStar Omega BMG LabTech plate reader. All experiments were performed with six technical replicates across three independent occasions.

### Histology

Rat second and third order mesenteric arteries were isolated from the mesenteric arcade, cannulated and any remnants of blood were flushed. Vessels were infected with *K.* *pneumoniae* intraluminally for 1 h before fixation in 4% paraformaldehyde solution for 1 h. Fixed tissue was then washed thoroughly with PBS before being placed in 10% sucrose solution in PBS for 24 h and a further 24 h in 30% sucrose solution.

For cryosectioning, samples were embedded in OCT matrix and cut to produce 5 µm sections. Standard protocols were used for haematoxylin and eosin staining of the sections, which were then imaged on an Eclipse 80i microscope with a ×40 objective using the NIS-Elements acquisition programme by Nikon. Scoring was carried out in a blinded manner by one researcher from the laboratory who was not involved in this research. The scoring criteria are detailed in Supplementary Table 3.

Six vessels from five different rats were analysed.

### RNA isolation and RT–qPCR

RNA was isolated and extracted by lysing cells in TRIzol reagent using the manufacturer’s provided guidance. RNA concentration and purity were determined using a Nanodrop spectrophotometer by assessing the A_260_/A_280_ ratio. Duplicate cDNA preparations from each sample were generated from 1 μg of RNA using Moloney murine leukaemia virus (M-MLV) reverse transcriptase (Sigma-Aldrich) according to the manufacturer’s instructions. Quantitative real-time PCR analysis of gene expression was undertaken using a KAPA SYBR FAST qPCR kit and a Rotor-Gene Q real-time PCR cycler system (Qiagen). Thermal cycling conditions were as follows: 95 °C for 3 min for enzyme activation, 40 cycles of denaturation at 95 °C for 10 s and annealing at 60 °C for 20 s. Primers used in qPCR reactions are listed in Supplementary Table 2. cDNA samples were tested in duplicate, and relative mRNA quantity was determined using the comparative threshold cycle ($\Delta {\Delta }^{{{\rm{C}}}_{T}}$) method, with human *GAPDH* normalization.

### Mouse infections

*K.* *pneumoniae* strains were grown in 5 ml LB at 37 °C, collected at mid-exponential phase (2,500*g*, 20 min) and adjusted to an optical density of 1.0 at 600 nm in PBS (5 × 10^8^ CFU ml^−1^). Mice were infected with 1 × 10^3^ CFU (100 µl) of bacterial suspension or mock-infected with sterile PBS via intraperitoneal route of administration. Bacterial doses were also confirmed retrospectively by counting colony numbers after 24 h of incubation on LB agar plate at 37 °C. At 24 h after infection, mice were euthanized in accordance with an approved schedule 1 protocol. Lungs, livers and spleens were weighed, immersed in 1 ml PBS and processed for bacterial quantification. Samples were homogenized via mechanical disruption with a Precellys Evolution tissue homogenizer (Bertin Instruments), using 1.4 mm ceramic (zirconium oxide) beads at 4,500 rpm for 7 cycles of 10 s, with a 10 s pause between each cycle. Homogenates were serially diluted in sterile PBS and plated onto *Salmonella–Shigella* agar (Sigma-Aldrich) for the identification of *K.* *pneumoniae* colonies. Colonies were enumerated after overnight incubation at 37 °C. The plating detection limits were as follows: 10 CFU g^−1^ for lung, 2 CFU g^−1^ for liver and 20 CFU g^−1^ for spleen.

### Blood pressure measurement

Blood pressure was measured non-invasively on conscious mice using a tail-cuff system based on volume pressure recording technology (CODA, Kent Scientific). Before the experiments, a 5-day habituation period allowed the mice to acclimate to the plethysmography procedure. Following infection, systolic, diastolic and mean arterial pressures were recorded at 6, 12 and 24 h after infection. Mice were placed in individual restrainers and maintained at a controlled temperature (30–34 °C) to promote tail vasodilation. For each animal and time point, multiple measurement cycles were performed, and the mean of at least three stable recordings was used for analysis. Data were processed using the manufacturer’s software, with readings exhibiting motion artefacts or irregular waveforms excluded from analyses.

### Statistical analysis

No animals or data points were excluded from analyses. Data collection and analyses were not performed blind to the conditions of the experiments No statistical methods were used to predetermine sample sizes, but our sample sizes were similar to those reported in previous publications^33,34,43^. Data are presented as the mean with s.d. Two groups of data were compared using an unpaired, two-tailed Student’s *t*-test. Multiple groups were compared using a one-way or two-way ANOVA followed by Tukey’s or Dunnet’s multiple group comparison correction, respectively. For concentration–response curves, curves were fit using log (agonist) versus response (three parameters) approach and significance was determined using two-way ANOVA, including Dunnet’s multiple group comparison correction. Normality and equal variance assumptions were tested with the Kolmogorov–Smirnov test and the Brown–Forsythe test, respectively. Analyses were performed using Prism software (GraphPad, v.9.02).

### Reporting summary

Further information on research design is available in the Nature Portfolio Reporting Summary linked to this article.

## Supplementary information

Reporting Summary

Peer Review File

Supplementary Tables Table S1: list of bacterial strains. Table S2: primers used in the study. Table S3: histology scoring criteria.

Supplementary Video 1 Video showing the effect of 100 nM ACh on control (non-infected) vessel vasodilation.

Supplementary Video 2 Video showing the effect of 1 µM ACh on control (non-infected) vessel vasodilation.

Supplementary Video 3 Video showing the effect of infection with *Kp*43816 on 100 nM ACh-indued vasodilation.

Supplementary Video 4 Video showing the effect of infection with *Kp*43816 on 1 µM ACh-induced vasodilation.

Supplementary Video 5 Video showing the effect of 100 nM ACh on single-cell calcium signalling in control (non-infected) and infected vessels with *Kp*43816.

## Source data

Source Data Fig. 1 Uncropped western blots.

## Data Availability

All relevant data are presented in the manuscript, the Extended Data figures and the Supplementary information. Should any raw data files be needed in another format, they are available from the corresponding author. Source data are provided with this paper.

## Code availability

This manuscript does not include original code.
