# Supplementary material for: Klebsiella pneumoniae inhibits vasodilation through capsule and T6SS-dependent pathways
Source: Nat Microbiol. 2026 Jul 21;11(8):2170–86. doi: 10.1038/s41564-026-02425-0 (PMC13423877; doi:10.1038/s41564-026-02425-0)
Supplement: Supplementary file 3 — Table S1: list of bacterial strains. Table S2: primers used in the study. Table S3: histology scoring criteria. [file 41564_2026_2425_MOESM3_ESM.pdf]

Table S1. Bacterial strains used in this work.

Strain	Genotype or comments.	Reference
Klebsiella pneumoniae		
NTUH-K2044	Clinical isolate; serotype O1:K2; sequence type ST23	1
ATCC43816	Clinical isolate; serotype O1:K2; sequence type ST493	ATCC
MRSN-14444	Clinical isolate; MRSN Diversity Panel; sequence type 4270	2
AKP5	Clinical isolate; carbapenem resistant	Karolinska Institute collection
43Δ*manCKm*	ATCC43816, Δ*manCKm*, *manC* gene inactivated, Km^R	3
43816-Δ*tssB*	ATCC43816, Δ*tssB, tssB* gene inactivated	4
43816-Δ*vgrG4*	ATCC43816, Δ*vgrG4, vgrG4* gene inactivated	This work
NTUH-Δ*clpV*	NTUH-K2044, ΔclpV*, clpV* gene inactivated	4
Yersinia enterocolitica		
Ye	pYV negative derivative of strain WA-314 serotype O:8, harbouring the pT3SS plasmids; $Spec^R$	5
YeVgrG4	Ye strain harbouring the plasmids pT3SS and pE_{53}-*vgrG4* with a VSVG tagged *vgrG4*; $Spec^R$ Cm^R	6
Escherichia coli		
JKE201	MFDpirΔ*mcrA*Δ(*mrr-hsd*RMS-*mcr*BC) *aac(3)3IV::lacI*^{q}	7

1. Wu, K.M., Li, L.H., Yan, J.J., Tsao, N., Liao, T.L., Tsai, H.C., Fung, C.P., Chen, H.J., Liu, Y.M., Wang, J.T., et al. (2009). Genome sequencing and comparative analysis of Klebsiella pneumoniae NTUH-K2044, a strain causing liver abscess and meningitis. Journal of Bacteriology *191*, 4492-4501.
2. Martin, M.J., Stribling, W., Ong, A.C., Maybank, R., Kwak, Y.I., Rosado-Mendez, J.A., Preston, L.N., Lane, K.F., Julius, M., Jones, A.R., et al. (2023). A panel of diverse Klebsiella pneumoniae clinical isolates for research and development. Microb Genom *9*. 10.1099/mgen.0.000967.
3. Cano, V., March, C., Insua, J.L., Aguilo, N., Llobet, E., Moranta, D., Regueiro, V., Brennan, G.P., Millan-Lou, M.I., Martin, C., et al. (2015). Klebsiella pneumoniae survives within macrophages by avoiding delivery to lysosomes. Cellular microbiology *17*, 1537-1560.
4. Storey, D., McNally, A., Astrand, M., Sa-Pessoa Graca Santos, J., Rodriguez-Escudero, I., Elmore, B., Palacios, L., Marshall, H., Hobley, L., Molina, M., et al. (2020). Klebsiella pneumoniae type VI secretion system-mediated microbial

competition is PhoPQ controlled and reactive oxygen species dependent. PLoS Pathog *16*, e1007969.

5. Wolke, S., Ackermann, N., and Heesemann, J. (2011). The Yersinia enterocolitica type 3 secretion system (T3SS) as toolbox for studying the cell biological effects of bacterial Rho GTPase modulating T3SS effector proteins. Cellular microbiology *13*, 1339-1357.
6. Sá-Pessoa, J., López-Montesino, S., Przybyszewska, K., Rodríguez-Escudero, I., Marshall, H., Ova, A., Schroeder, G.N., Barabas, P., Molina, M., and Curtis, T. (2023). A trans-kingdom T6SS effector induces the fragmentation of the mitochondrial network and activates innate immune receptor NLRX1 to promote infection. Nature Communications *14*, 871.
7. Harms, A., Liesch, M., Körner, J., Québatte, M., Engel, P., and Dehio, C. (2017). A bacterial toxin-antitoxin module is the origin of inter-bacterial and inter-kingdom effectors of Bartonella. PLoS genetics *13*, e1007077.

Table S2. Primers used in this study.

Name	Forward (5’-3’)	Reverse (5’-3’)	Purpose
h_PRKCA	AGAACGTGCACGAGGTGAA	ACCCACAGTGATCGCAGAAG	siRNA knockdown efficiency
h_PRKCB	GGCAGA AGA ACGTGCATGAG	CAA AACGTGGGGCTGGAG TA	siRNA knockdown efficiency
h_PRKCE	ACAAAATCACCAACAGCGGC	GTTGAACTCATCCAGGCCCA	siRNA knockdown efficiency
h_NLRX1	GTGCCCGGAAGCTGGGCTTG	CCGGGCACCACCTTCAGCAG	siRNA knockdown efficiency
h_GAPDH	GAGAAGGCTGGGGCTCATTT	AGTGATGGCATGGACTGTGG	siRNA knockdown efficiency
VgrG4UP	GAATTCGCAACGATAAATCCA ATCAA	GGATCCTATATTCCTGTTTTACTCCG	VgrG4 mutagenesis
VgrG4DOWN	GGATCCACAATGATA TTGTTACCCCG	GAATTCCGTTTATTC TCCTGCGGC	VgrG4 mutagenesis
VgrG4SCREEN	AACTTCCTCCGCTGAACCTG	TGCTTTGGCAAAATTTTCGTGC	VgrG4 mutagenesis

Table S3. Histology scoring criteria.

Damage	Score
Major disruption in endothelium	5
Major disruption in vascular smooth muscle layer	5
Minor disruption in endothelium	2
Minor disruption in vascular smooth muscle layer	2
Small breaks in endothelium	1
Small breaks in vascular smooth muscle layer	1
