# Supplementary material for: Klebsiella pneumoniae inhibits vasodilation through capsule and T6SS-dependent pathways
Source: Nat Microbiol. 2026 Jul 21;11(8):2170–86. doi: 10.1038/s41564-026-02425-0 (PMC13423877; doi:10.1038/s41564-026-02425-0)

Figure 4a

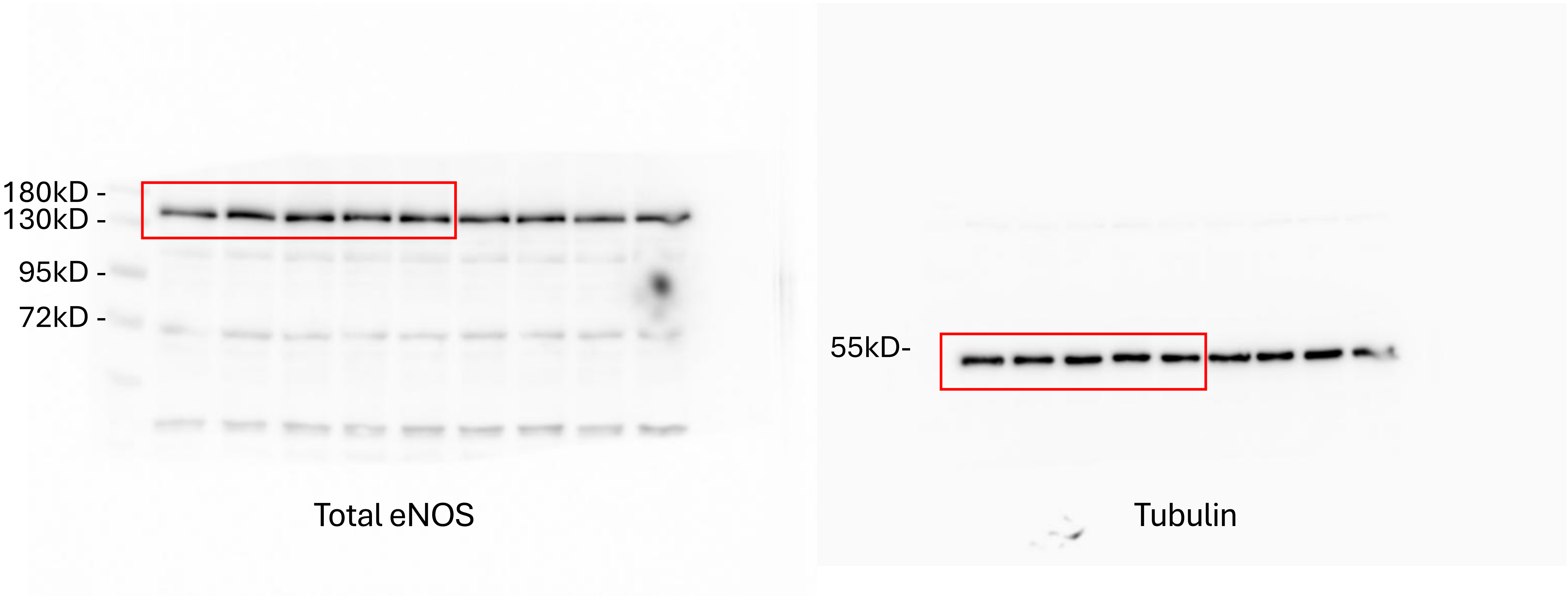

Figure 4b

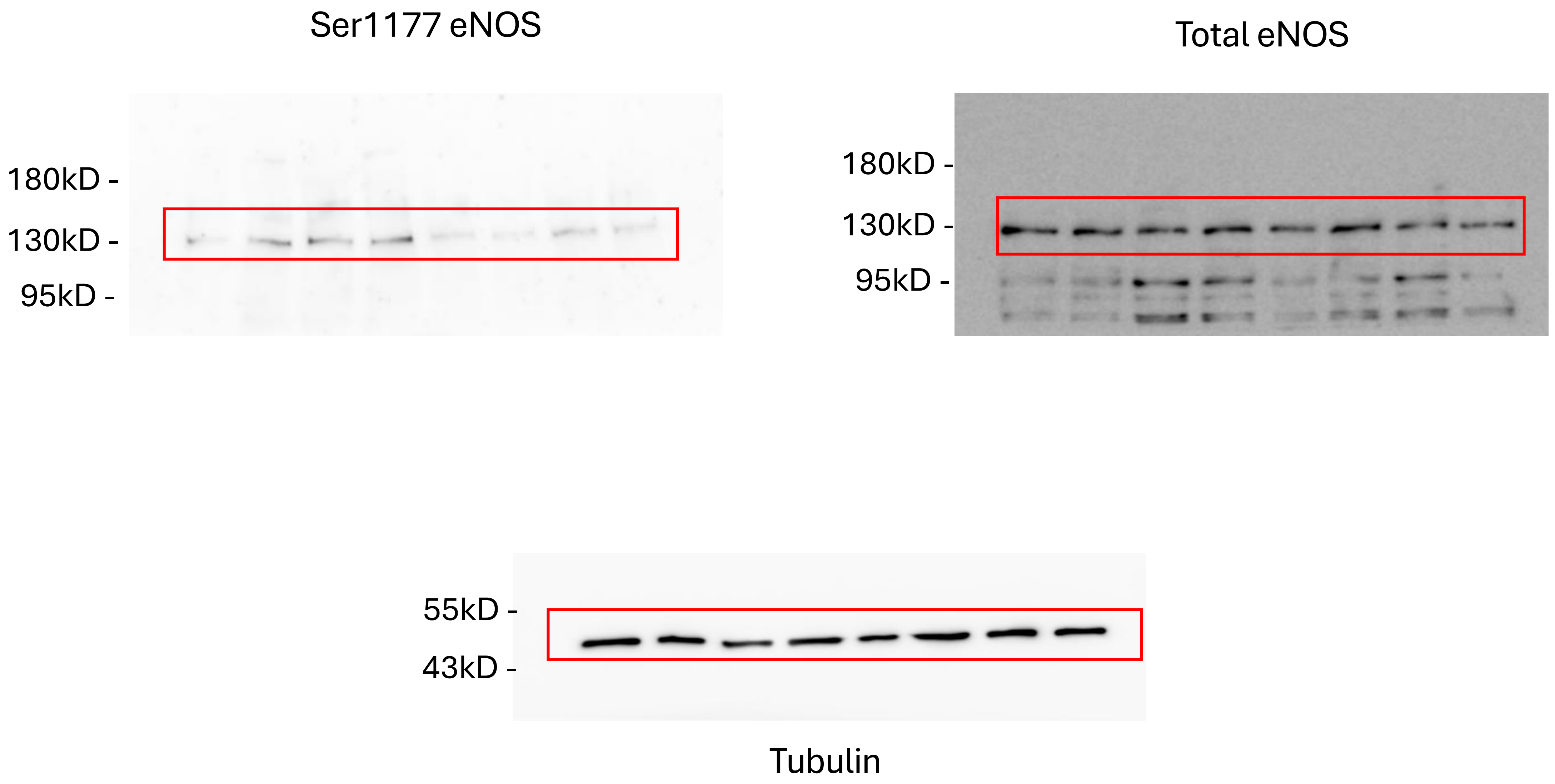
Ser1177 eNOS
180kD -
130kD -
95kD -
Total eNOS
180kD -
130kD -
95kD -
55kD -
43kD -
Tubulin

Figure 4c

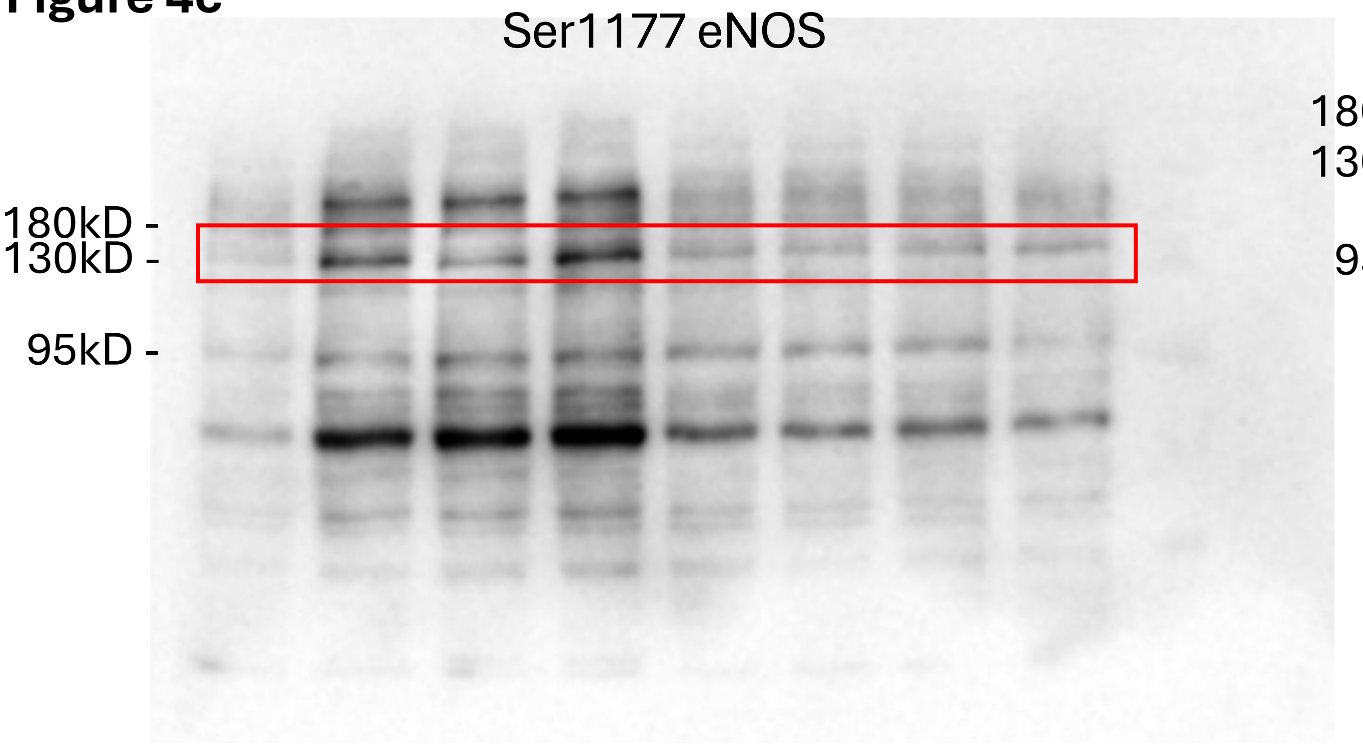
Ser1177 eNOS
180kD -
130kD -
95kD -

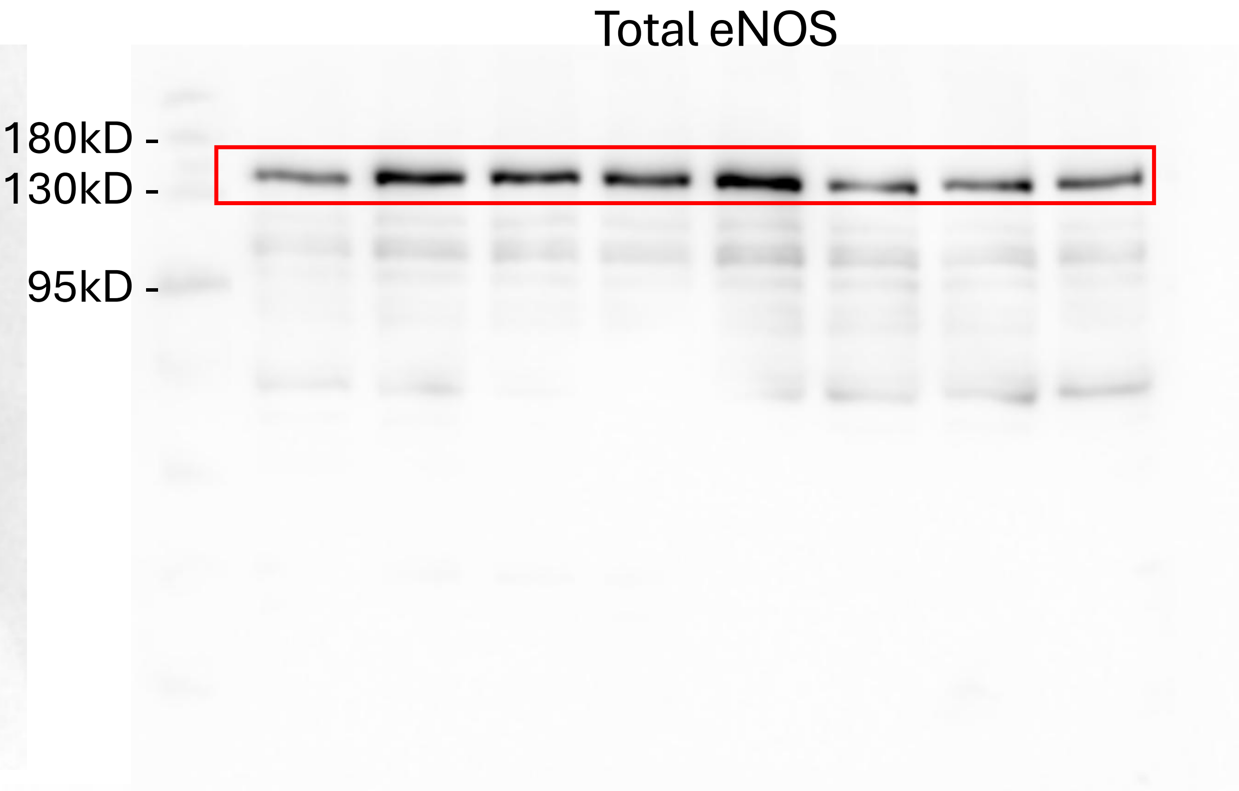
Total eNOS
180kD -
130kD -
95kD -

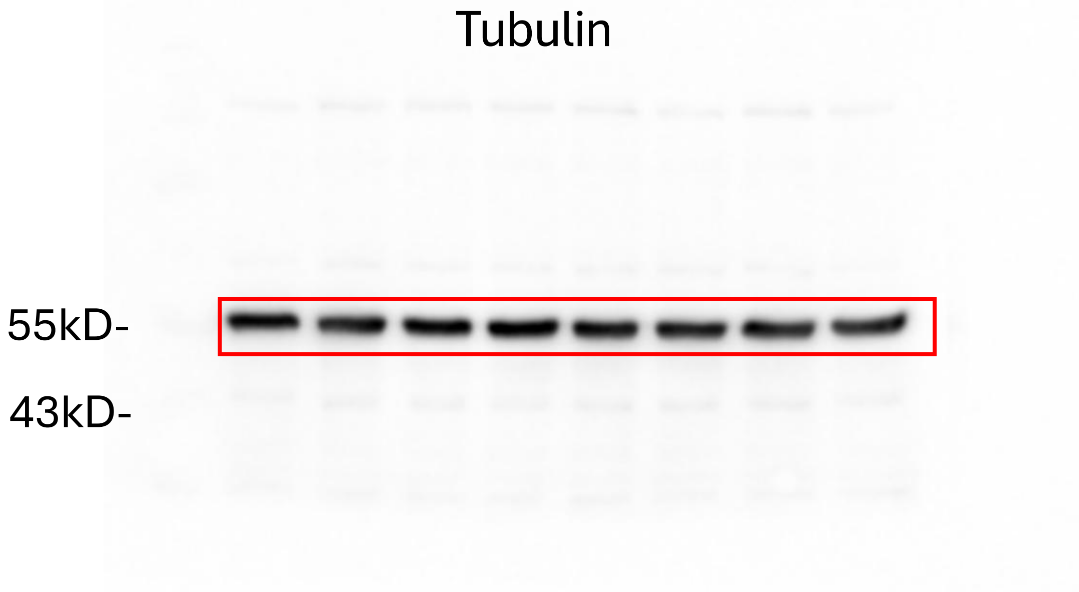
Tubulin
55kD-
43kD-

Figure 4d

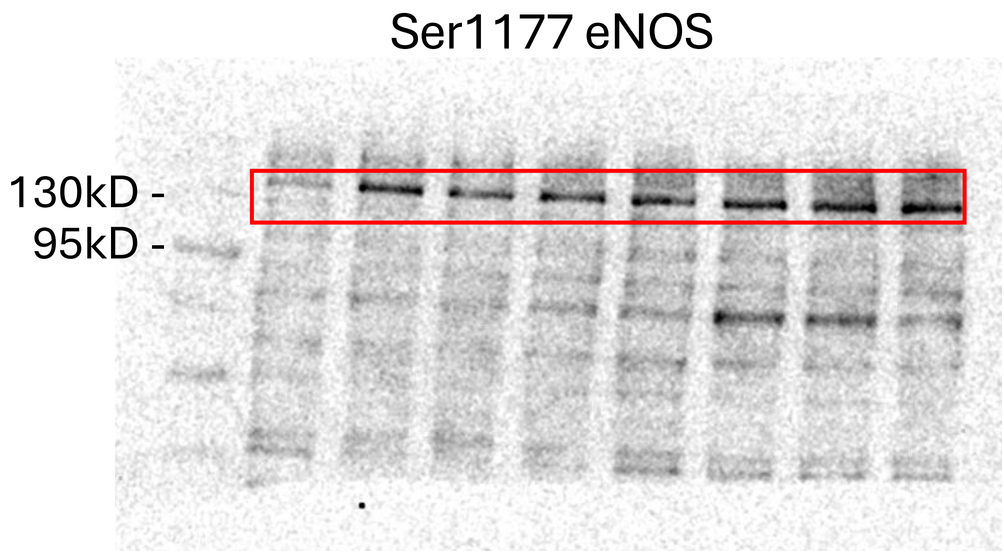

Ser1177 eNOS
130kD -
95kD -

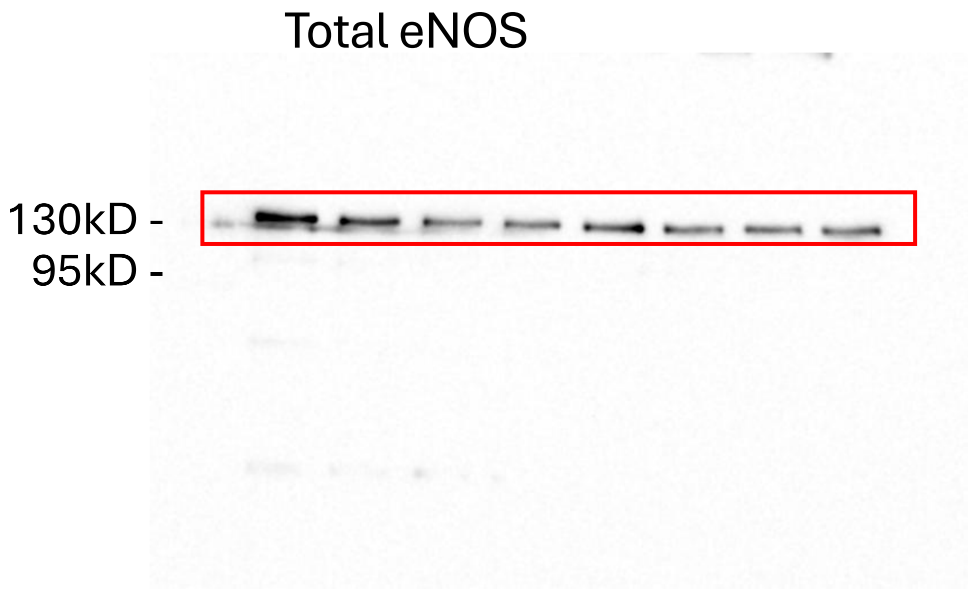

Total eNOS
130kD -
95kD -

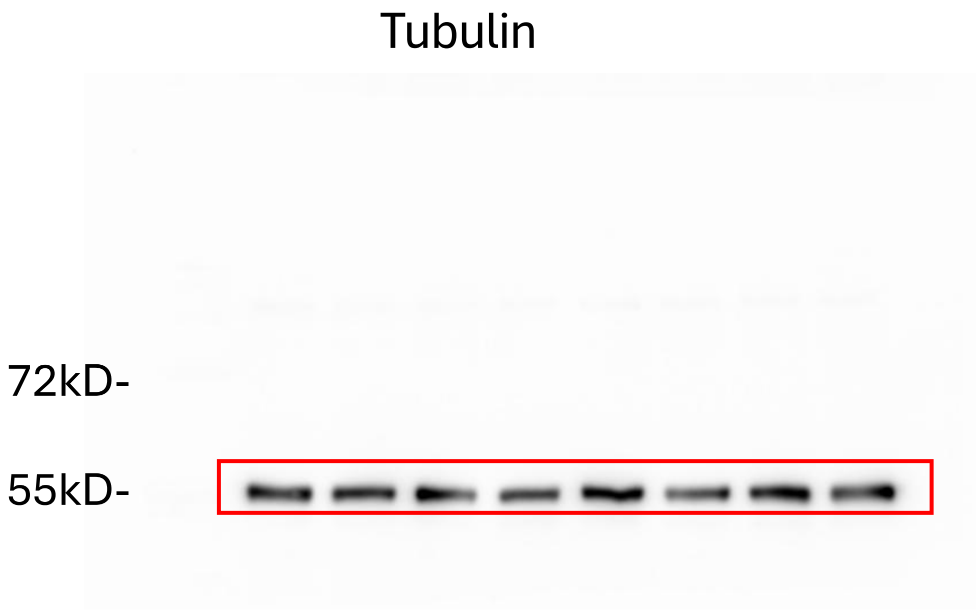

Tubulin
72kD-
55kD-

Figure 4e

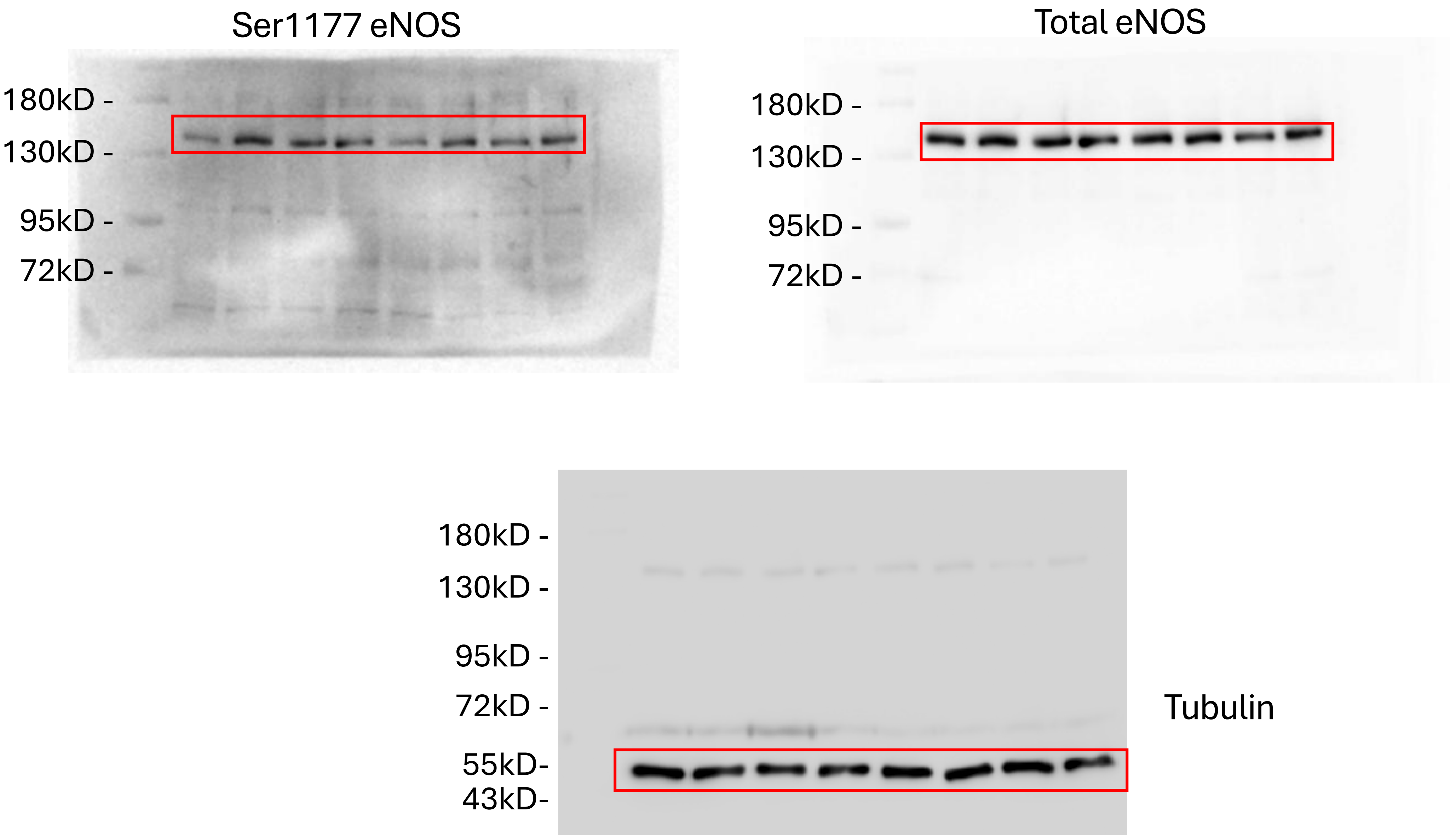
Ser1177 eNOS
180kD -
130kD -
95kD -
72kD -
Total eNOS
180kD -
130kD -
95kD -
72kD -
180kD -
130kD -
95kD -
72kD -
55kD-
43kD-
Tubulin

Figure 4f

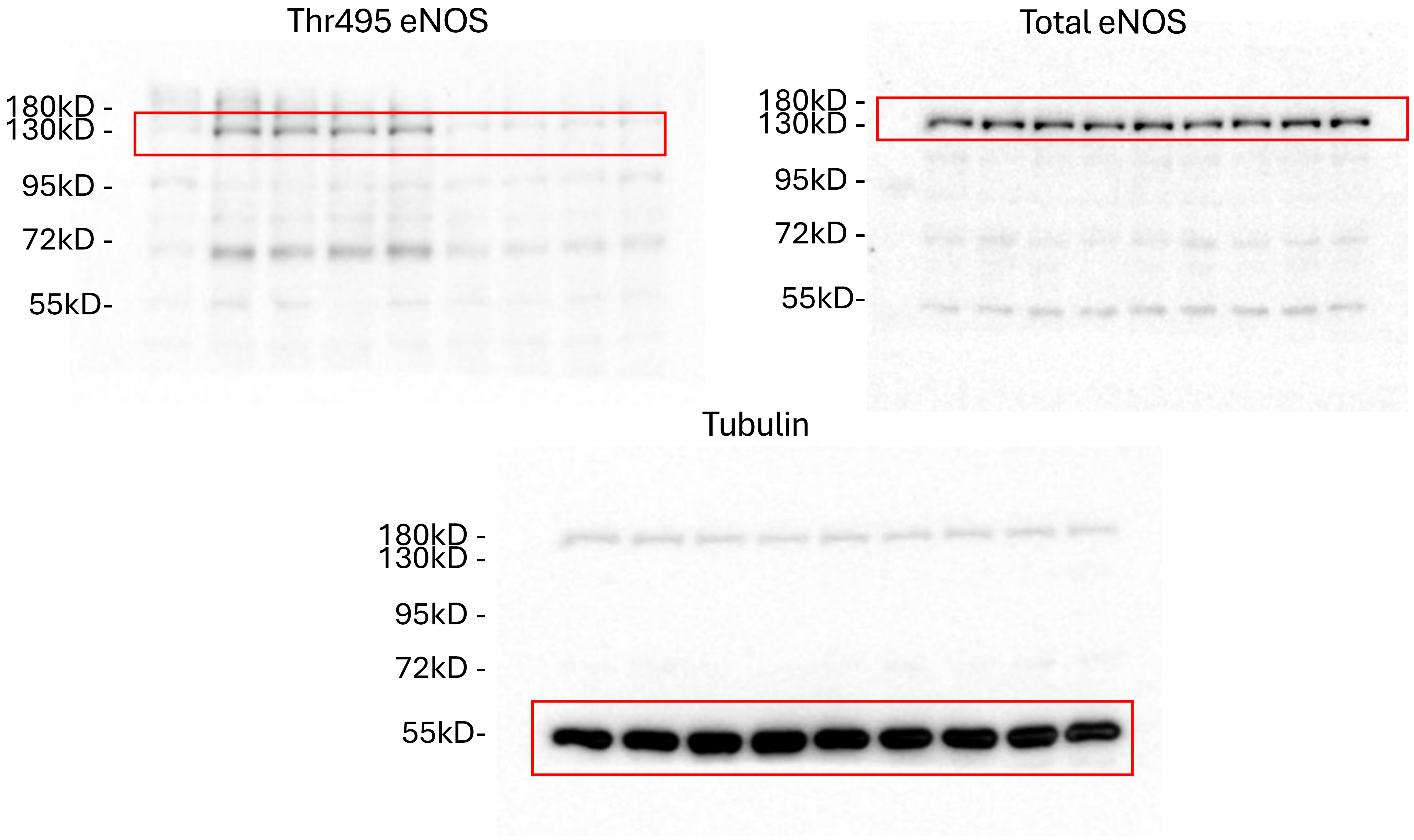
Thr495 eNOS
180kD -
130kD -
95kD -
72kD -
55kD-
Total eNOS
180kD -
130kD -
95kD -
72kD -
55kD-
Tubulin
180kD -
130kD -
95kD -
72kD -
55kD-

Figure 4g

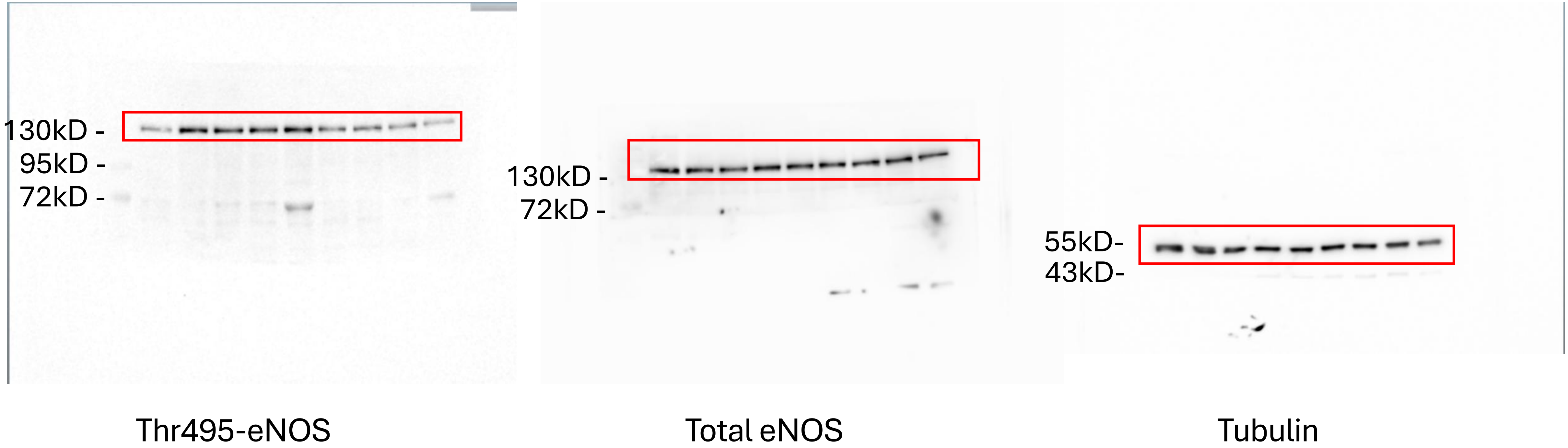
130kD -
95kD -
72kD -
130kD -
72kD -
55kD-
43kD-
Thr495-eNOS
Total eNOS
Tubulin

Figure 5a

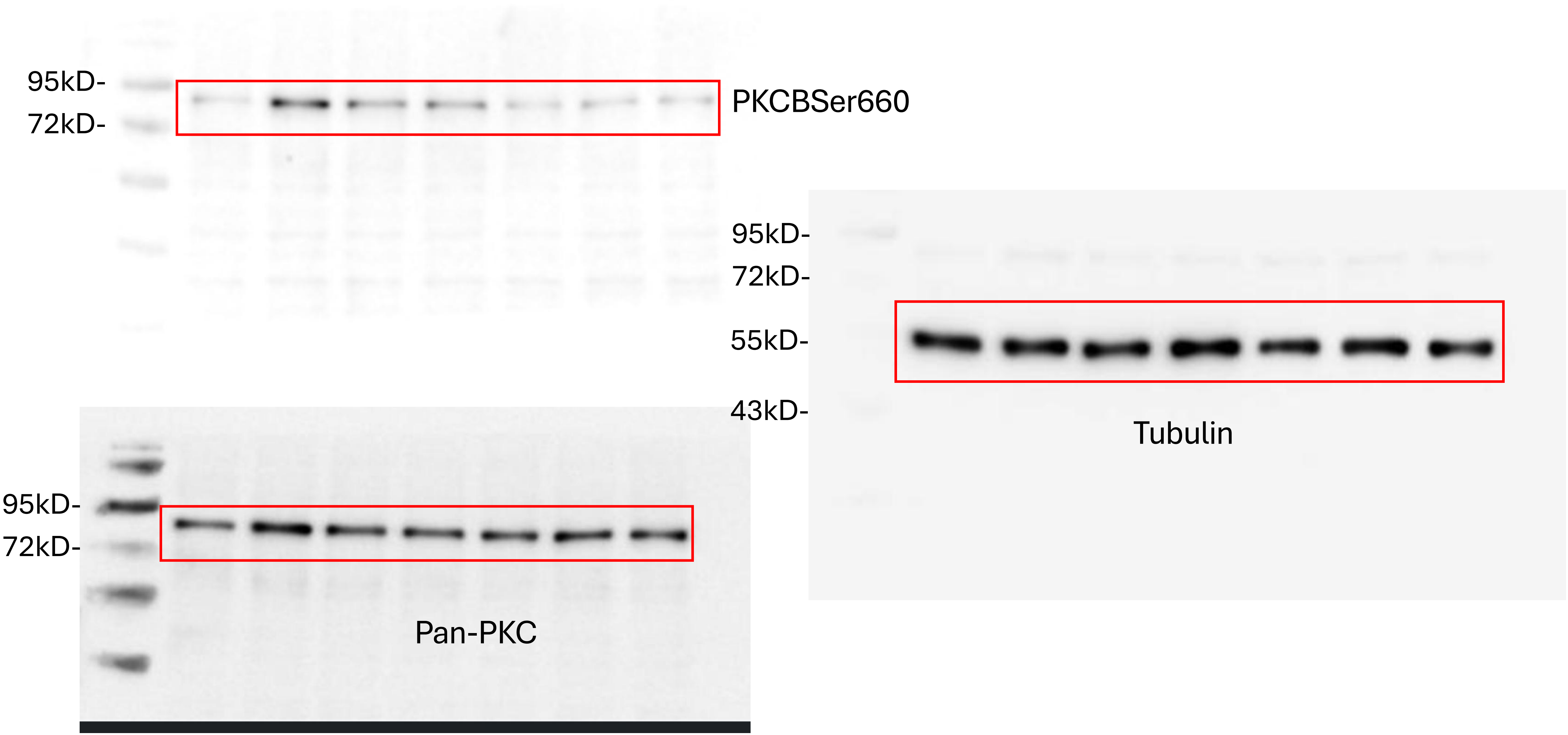
95kD-
72kD-
PKCBSer660
95kD-
72kD-
55kD-
43kD-
Tubulin
95kD-
72kD-
Pan-PKC

Figure 5b

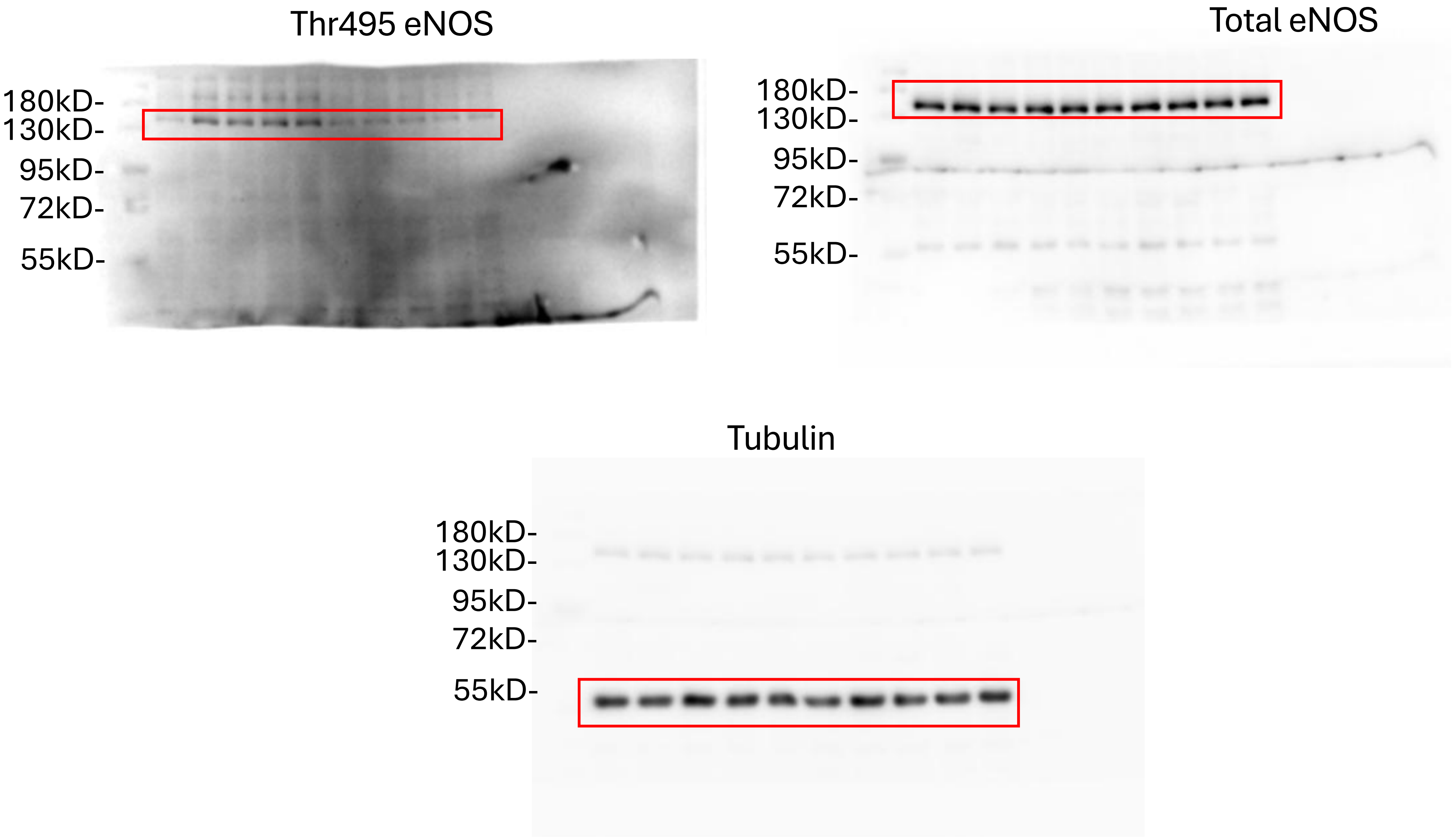
Thr495 eNOS
180kD-
130kD-
95kD-
72kD-
55kD-
Total eNOS
180kD-
130kD-
95kD-
72kD-
55kD-
Tubulin
180kD-
130kD-
95kD-
72kD-
55kD-

Figure 5c

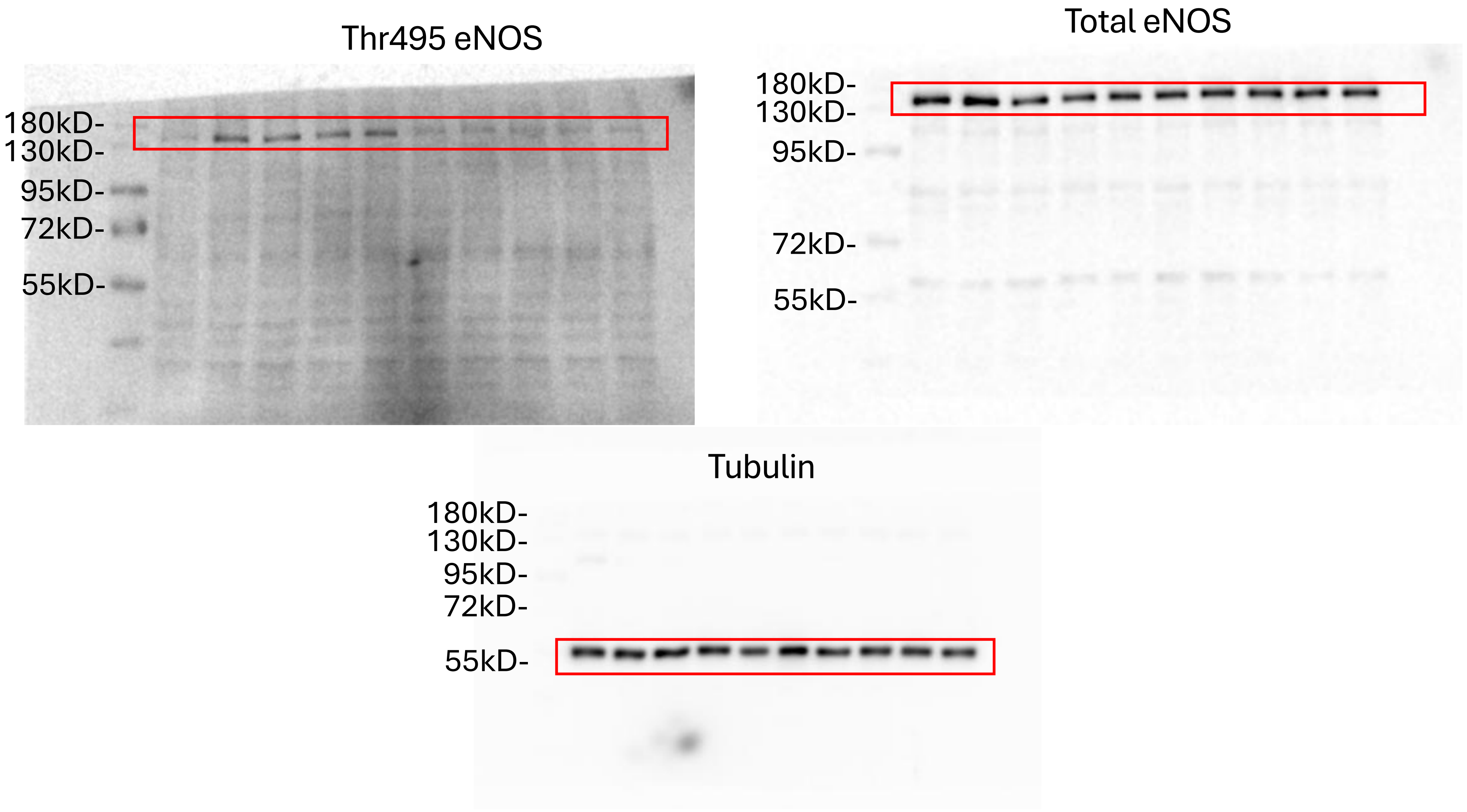
Thr495 eNOS
180kD-
130kD-
95kD-
72kD-
55kD-
Total eNOS
180kD-
130kD-
95kD-
72kD-
55kD-
Tubulin
180kD-
130kD-
95kD-
72kD-
55kD-

Figure 5e

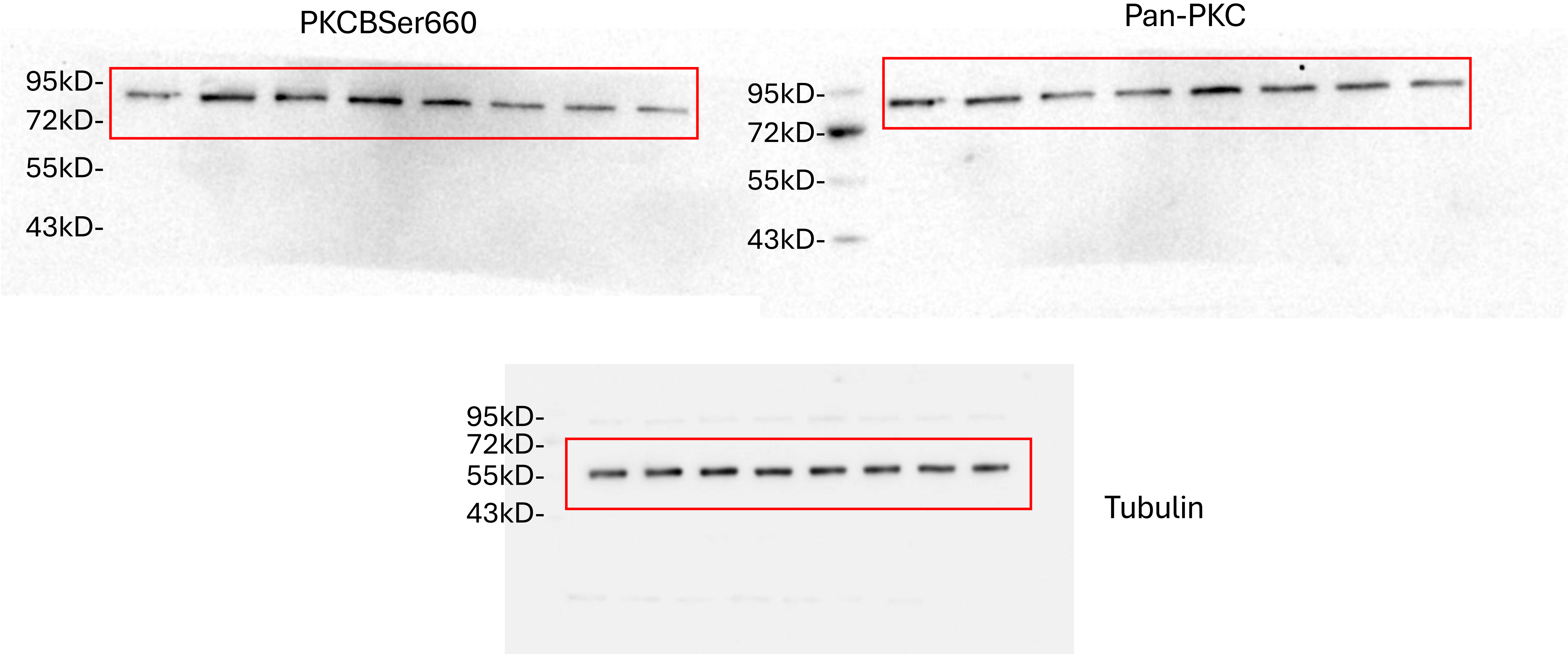
PKCBSer660
95kD-
72kD-
55kD-
43kD-
Pan-PKC
95kD-
72kD-
55kD-
43kD-
95kD-
72kD-
55kD-
43kD-
Tubulin

Figure 5f

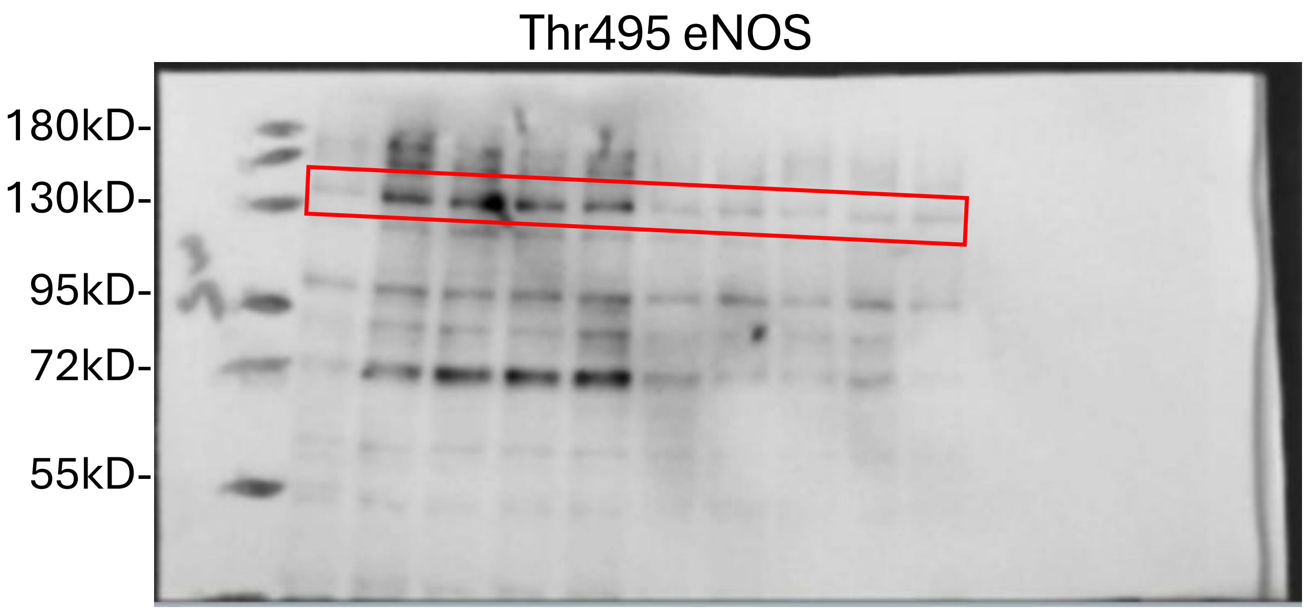
Thr495 eNOS
180kD-
130kD-
95kD-
72kD-
55kD-

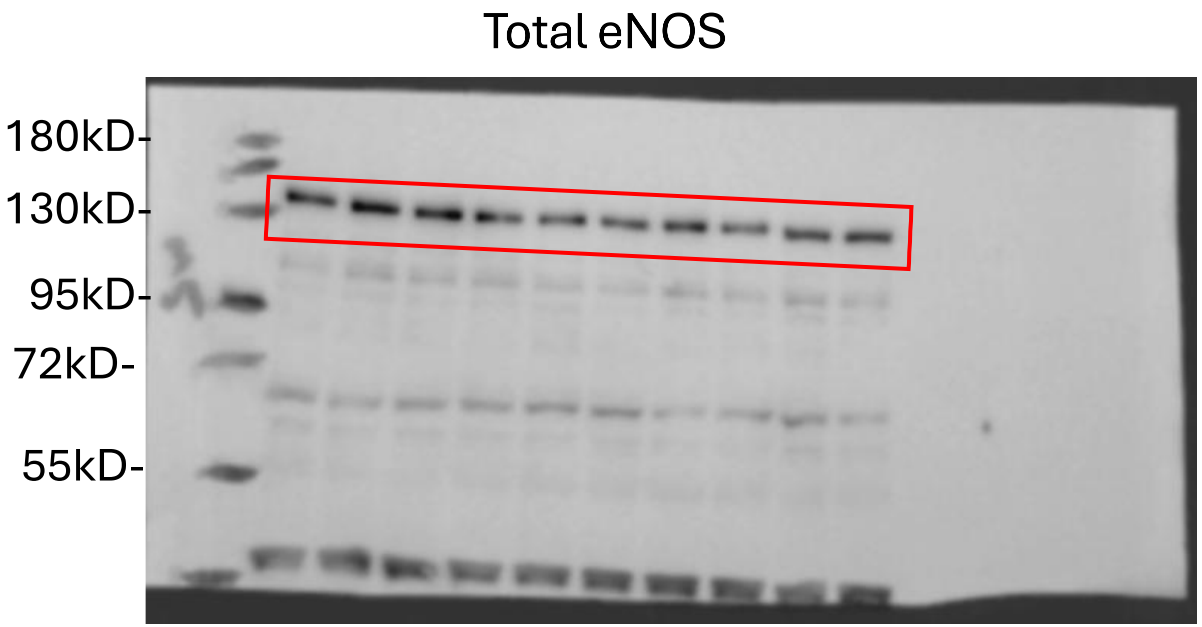
Total eNOS
180kD-
130kD-
95kD-
72kD-
55kD-

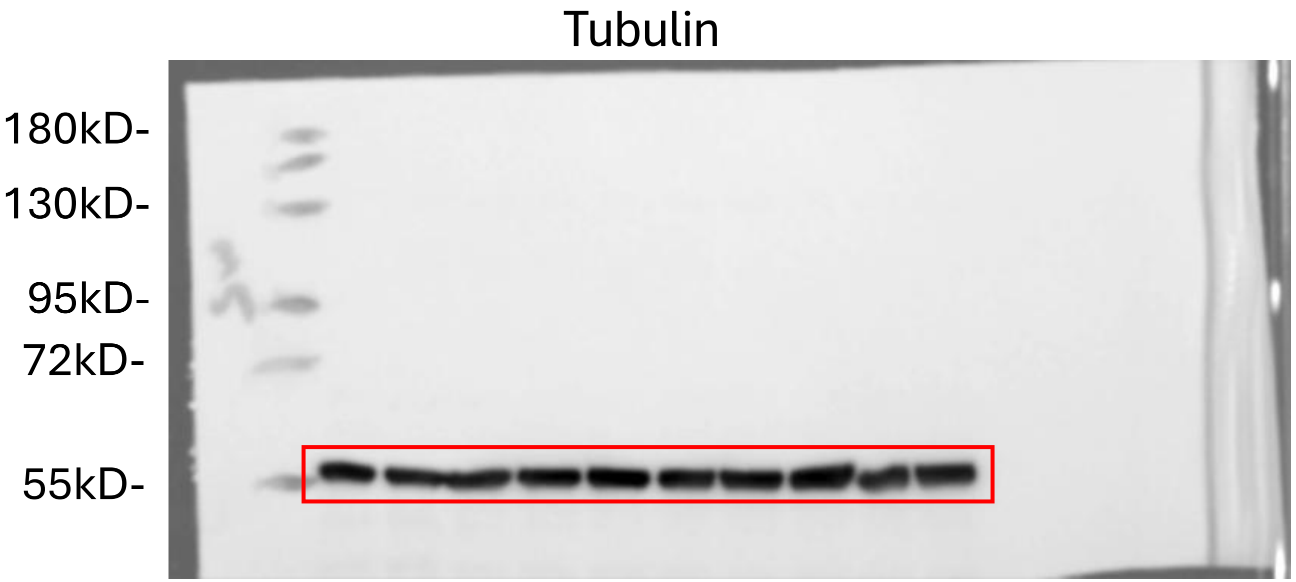
Tubulin
180kD-
130kD-
95kD-
72kD-
55kD-

Figure 5g

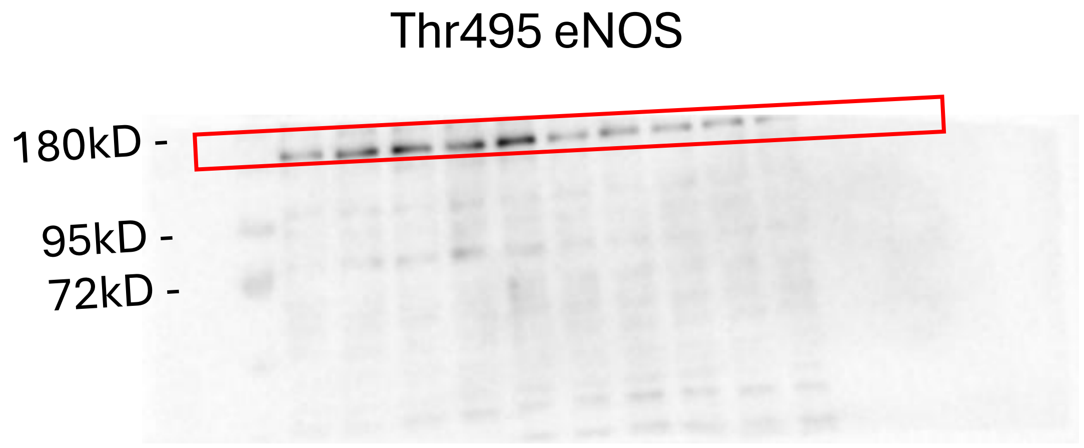
Thr495 eNOS
180kD -
95kD -
72kD -

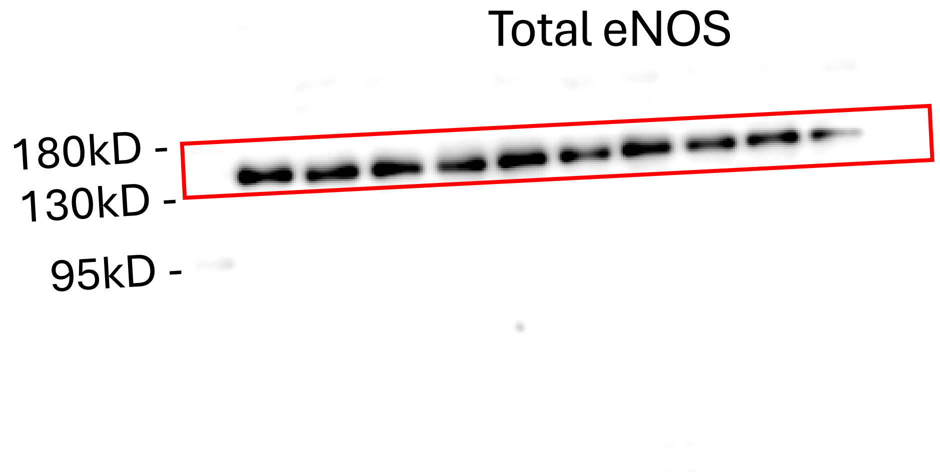
Total eNOS
180kD -
130kD -
95kD -

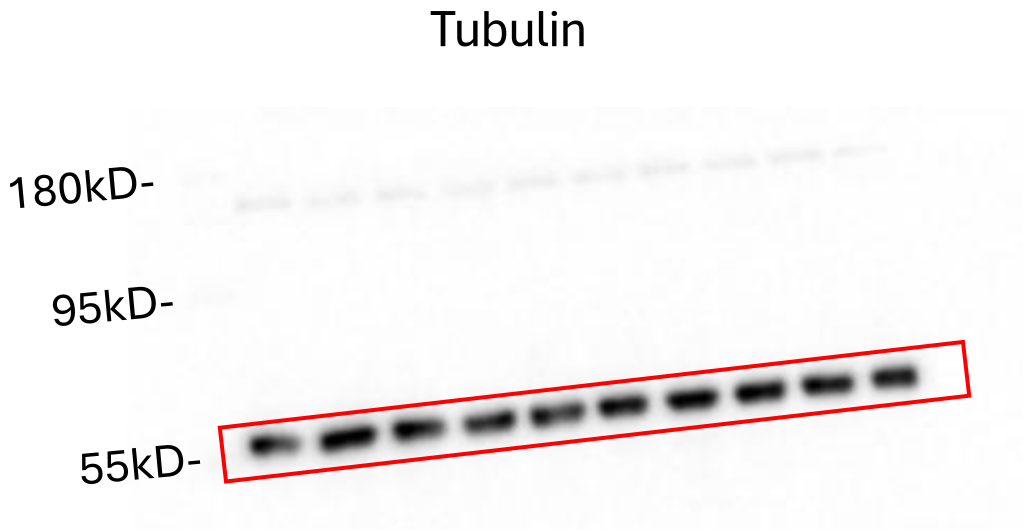
Tubulin
180kD-
95kD-
55kD-

Figure 5i

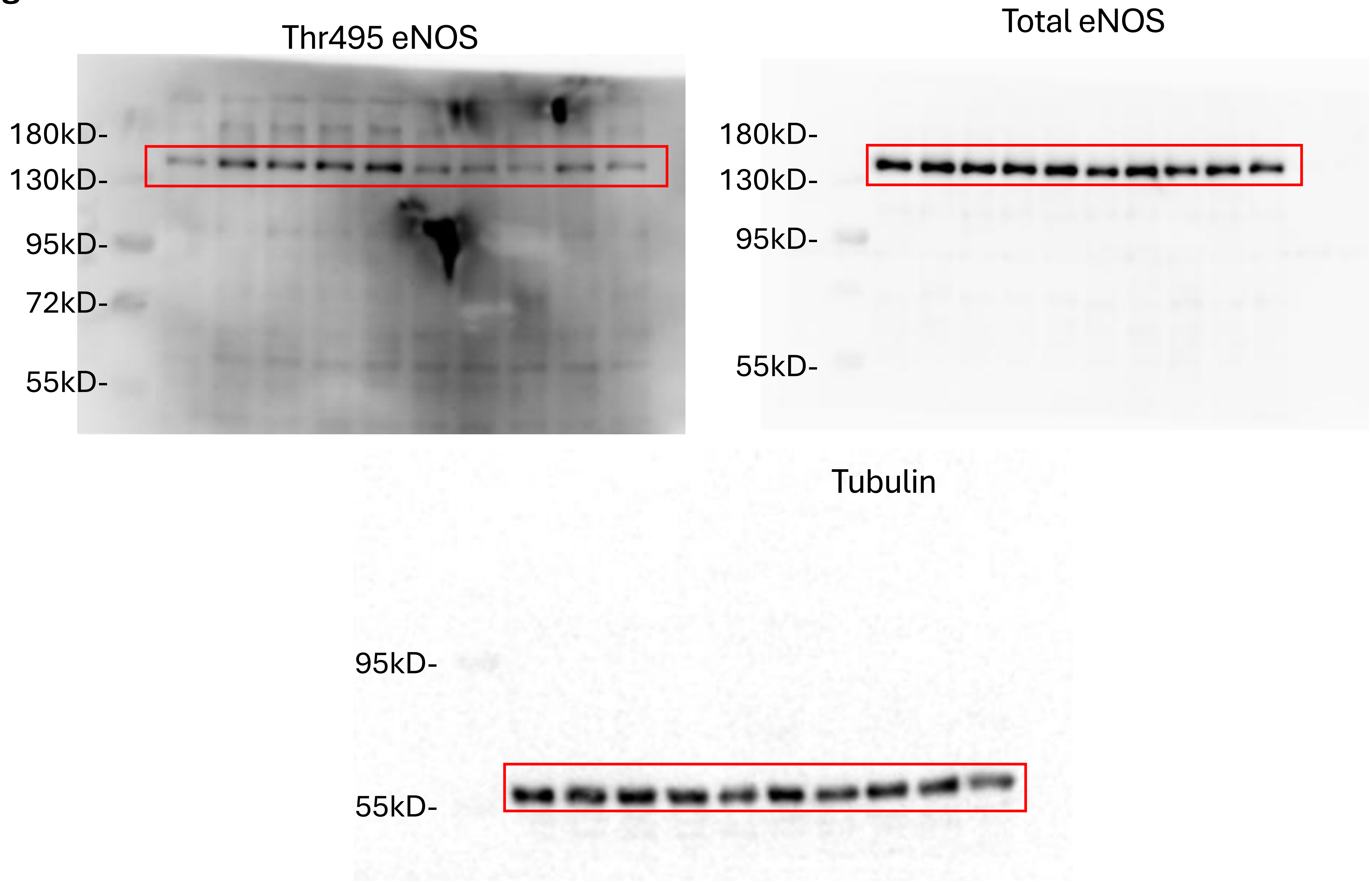

Thr495 eNOS
180kD-
130kD-
95kD-
72kD-
55kD-
Total eNOS
180kD-
130kD-
95kD-
55kD-
Tubulin
95kD-
55kD-

Figure 5j

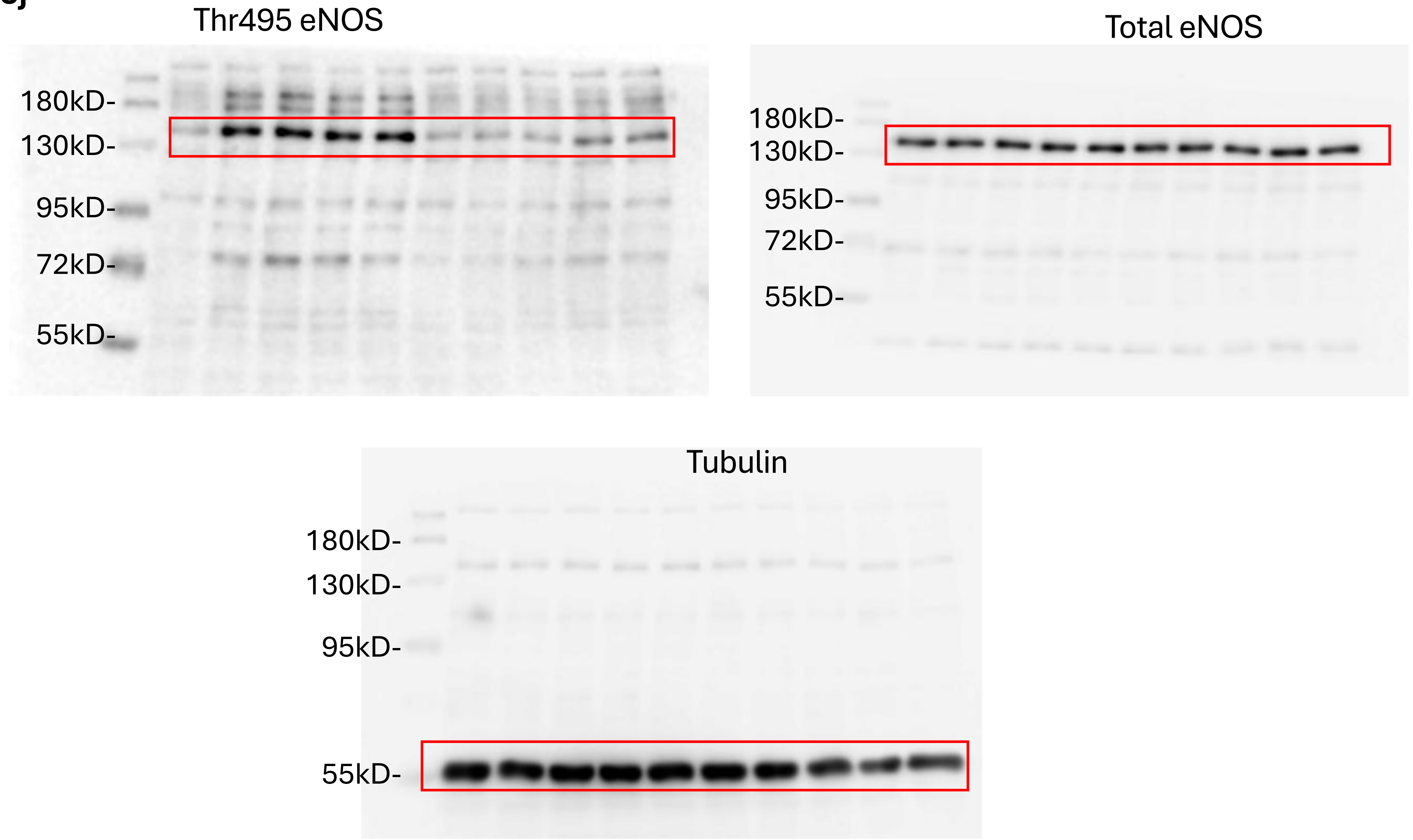
Thr495 eNOS
180kD-
130kD-
95kD-
72kD-
55kD-
Total eNOS
180kD-
130kD-
95kD-
72kD-
55kD-
Tubulin
180kD-
130kD-
95kD-
55kD-

Extended data figures

Extended data Figure 4a

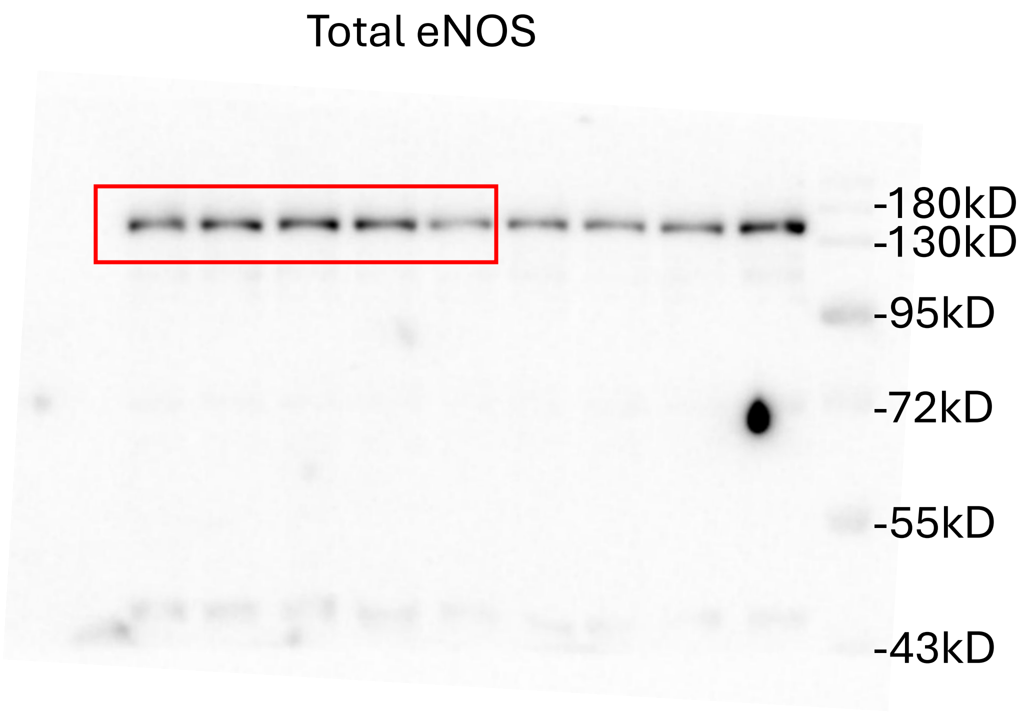

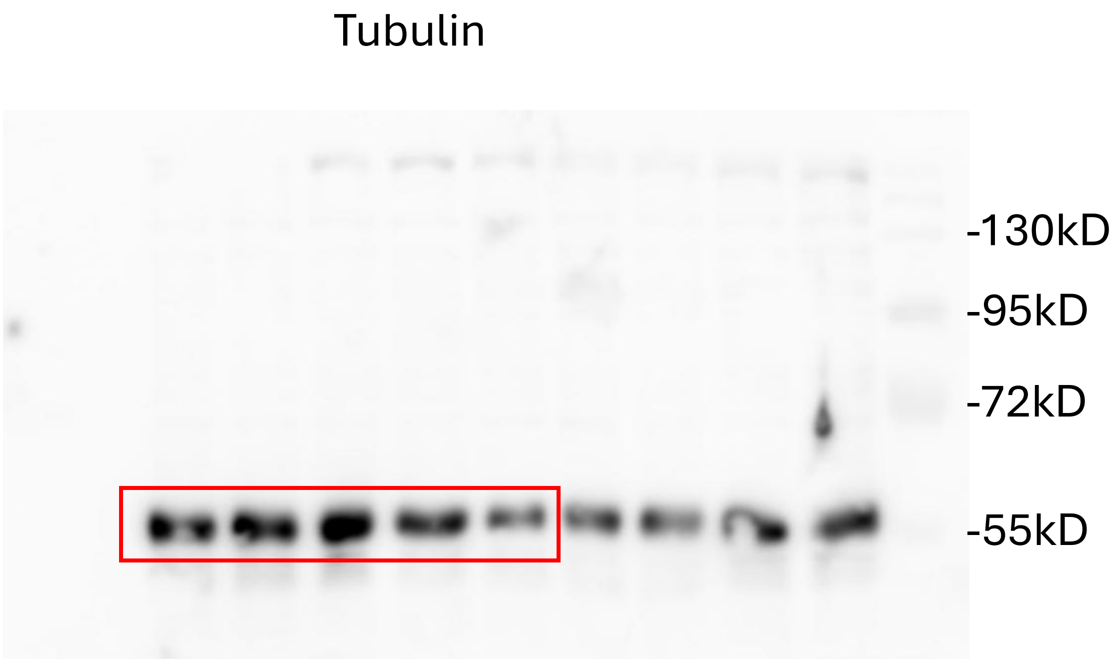

Extended data Figure 4b

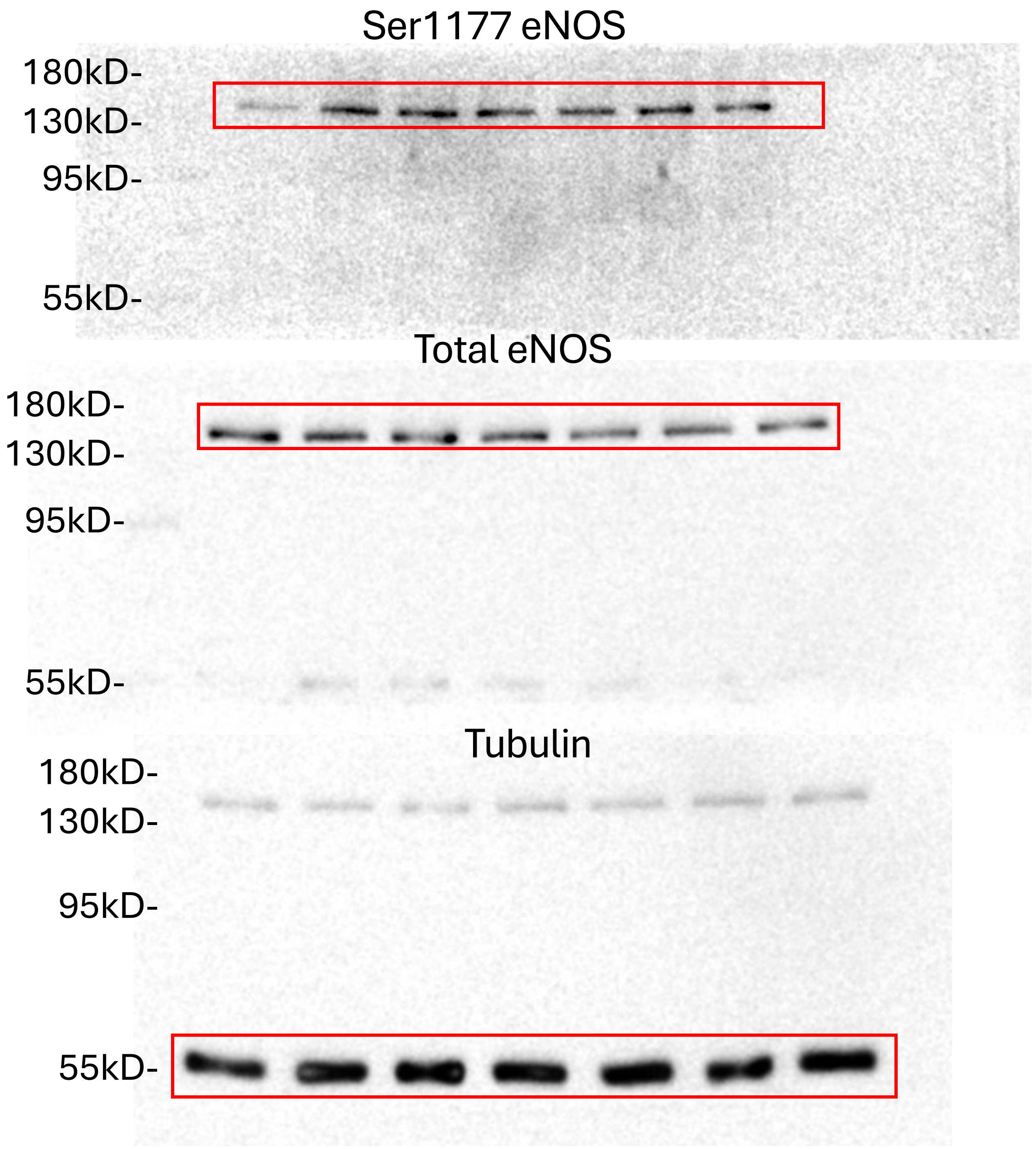

Extended data Figure 4c

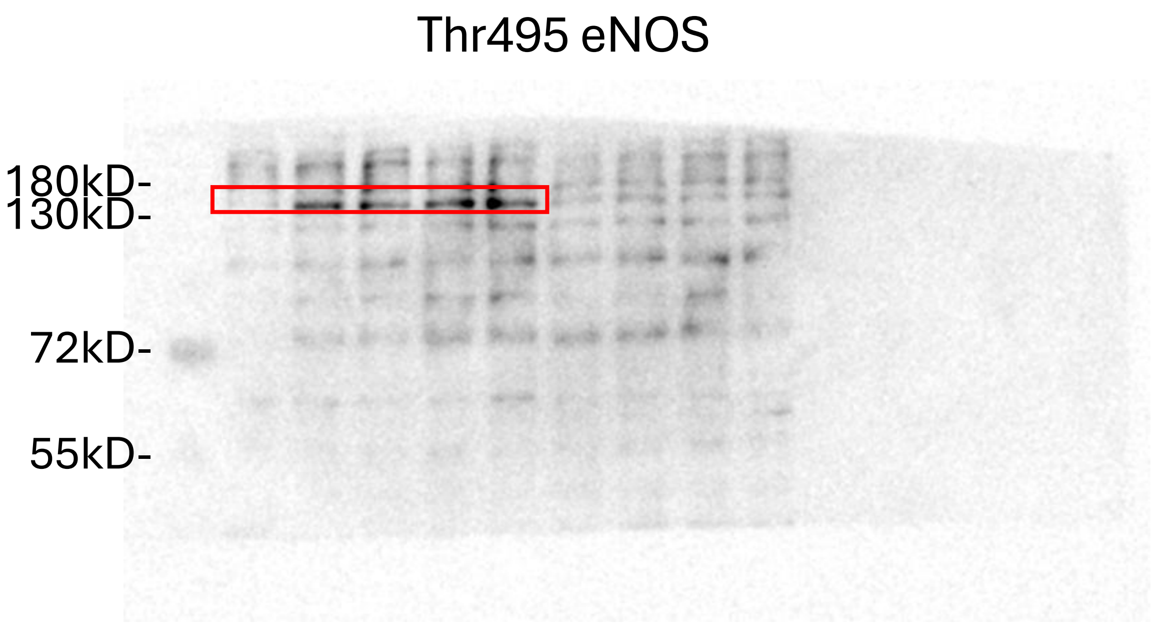

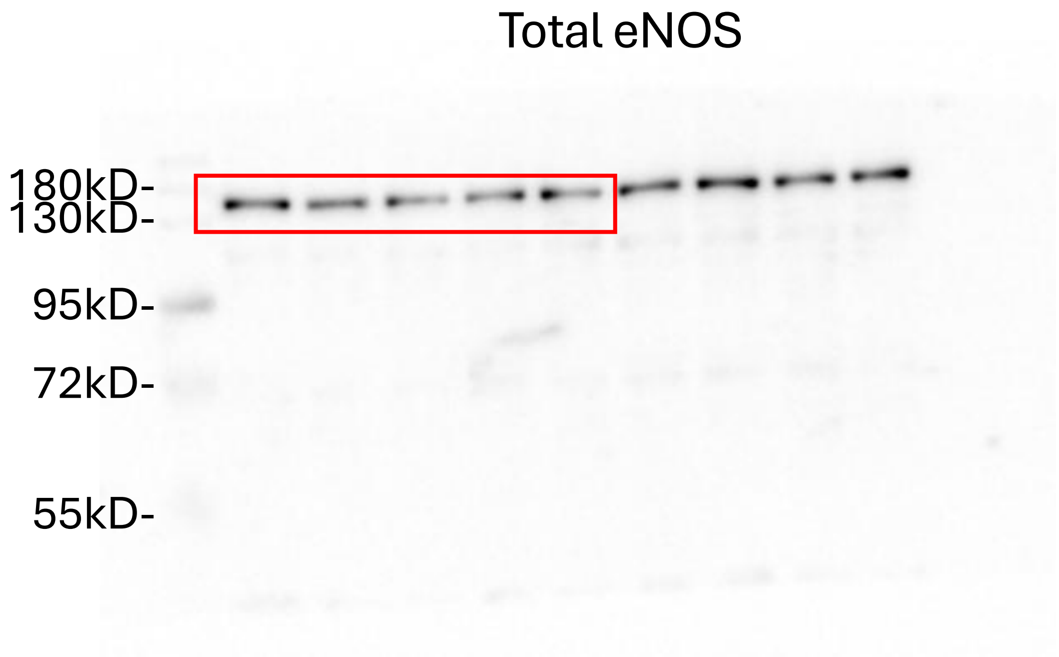

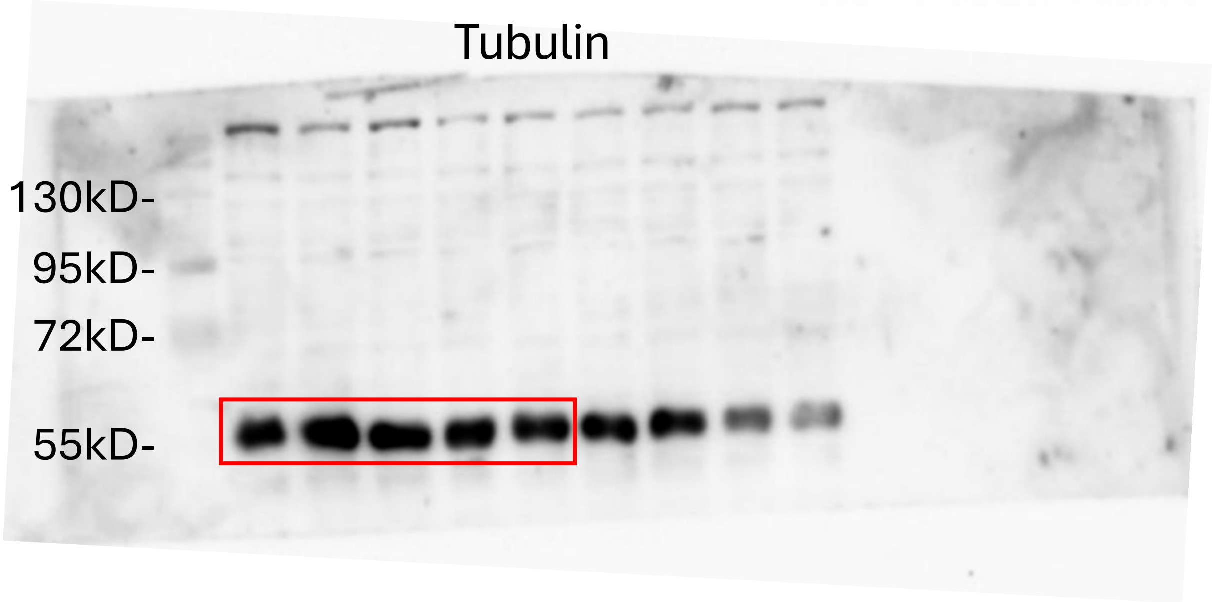

Extended data Figure 4d

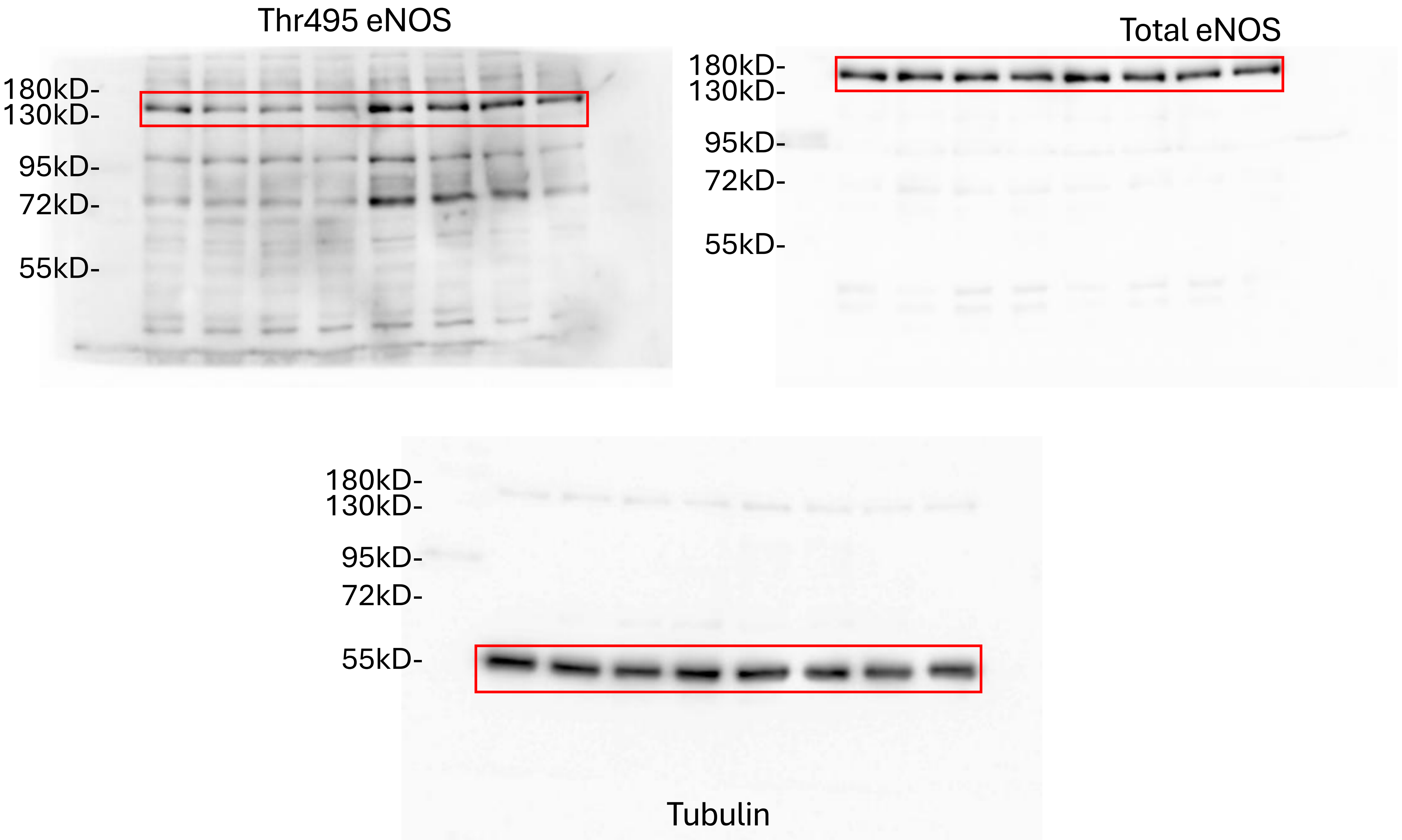

Extended data Figure 4e

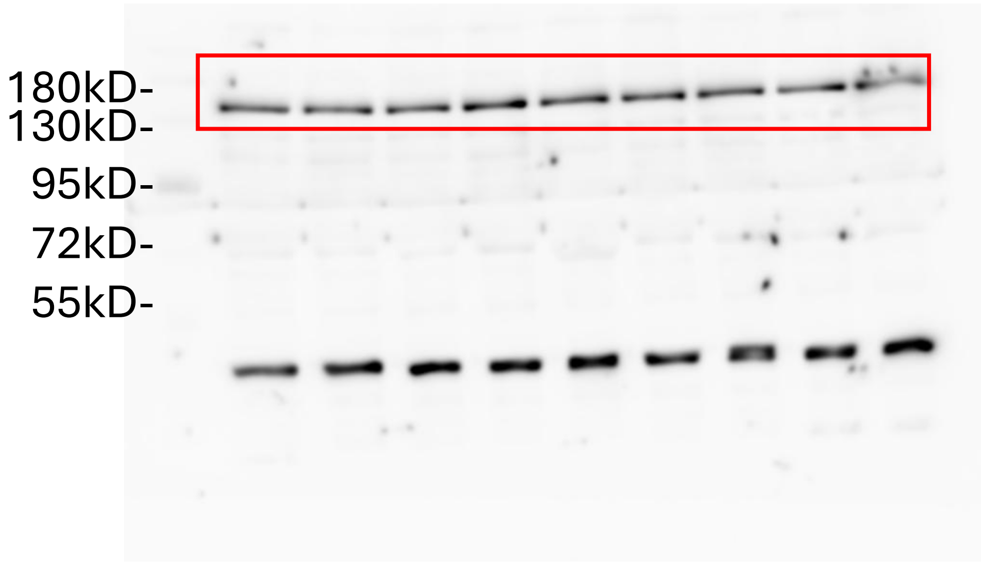

Total eNOS

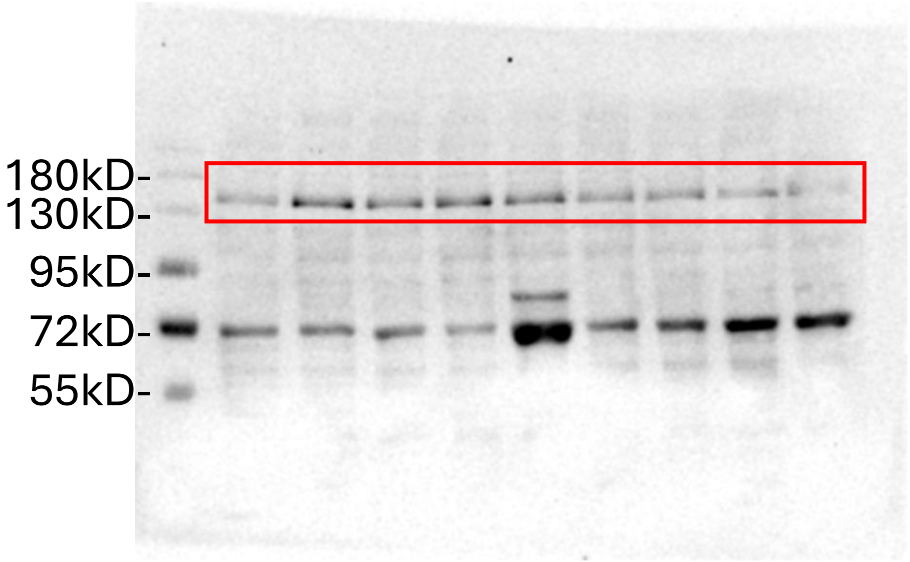

Thr495-eNOS

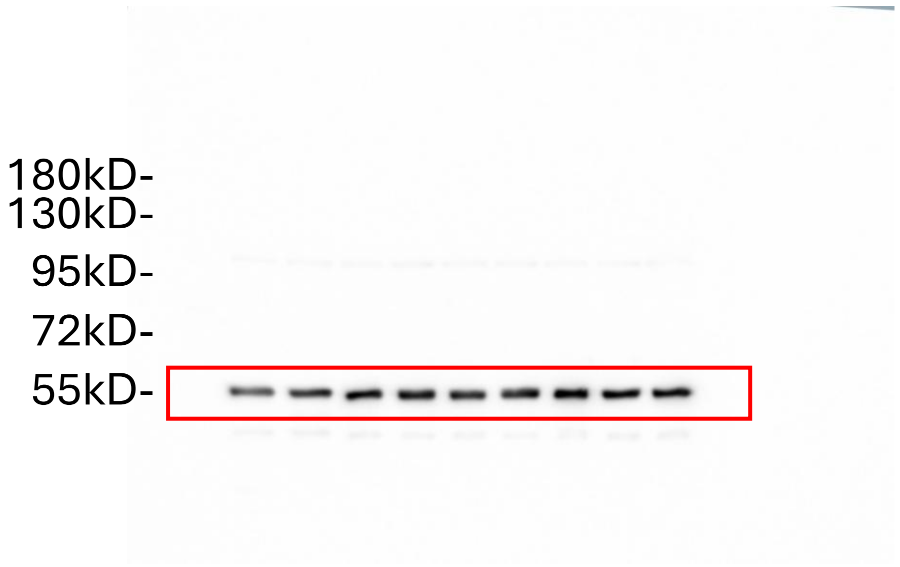

Tubulin

Extended data Figure 4f

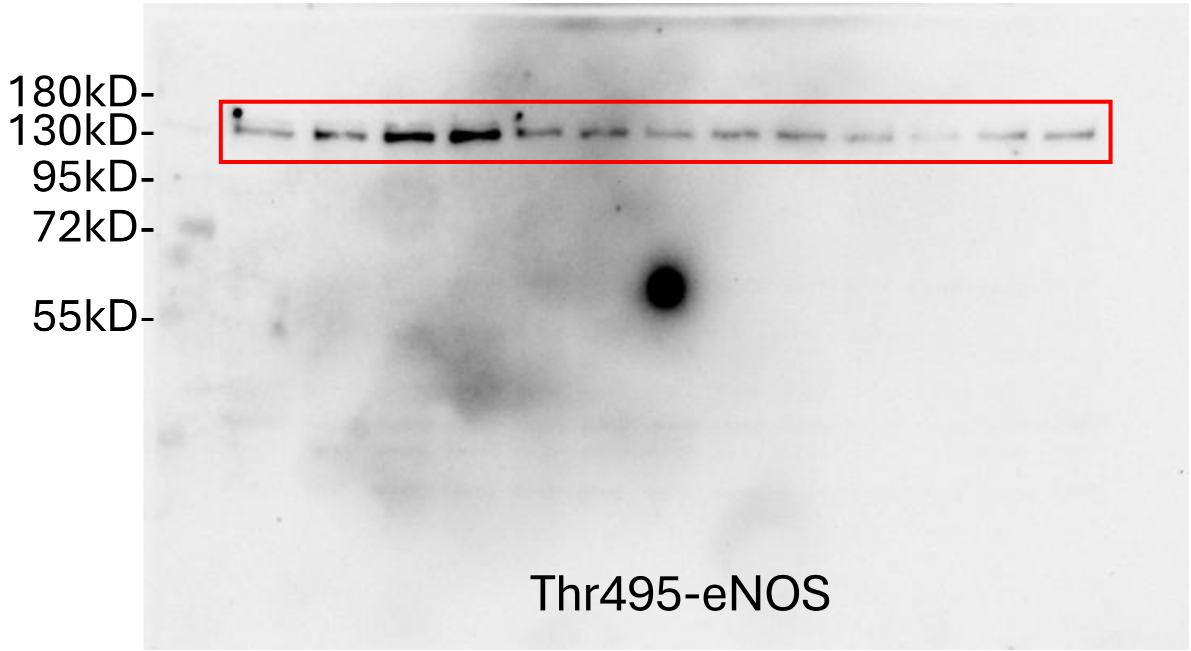

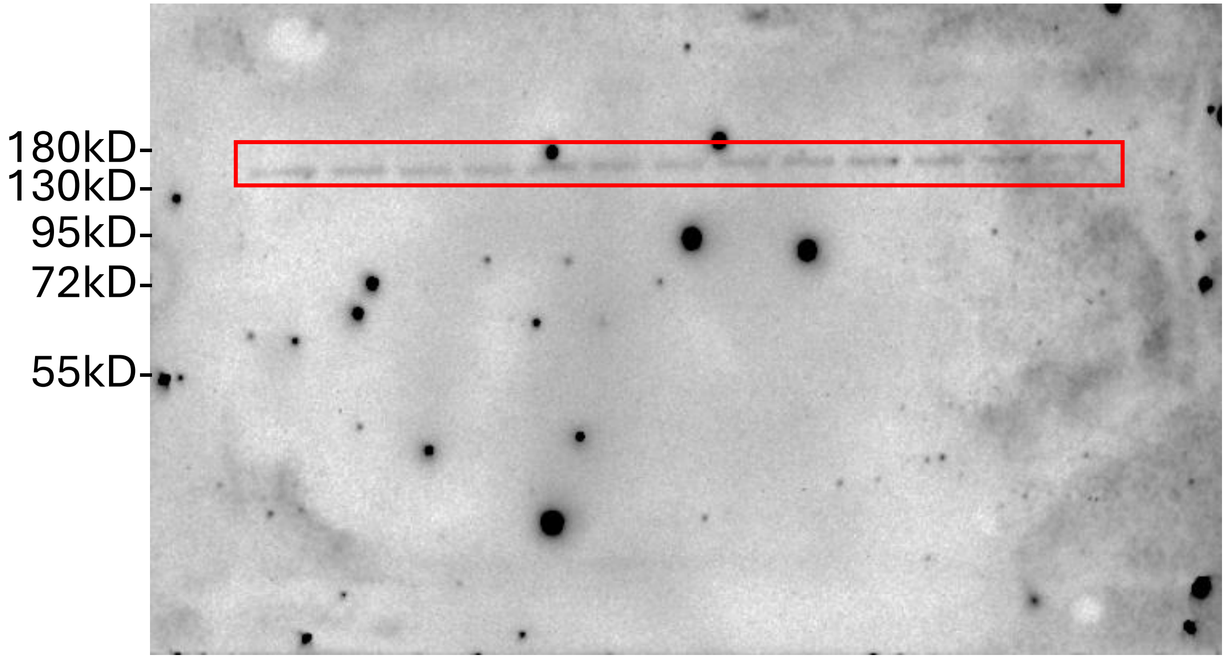

Total eNOS

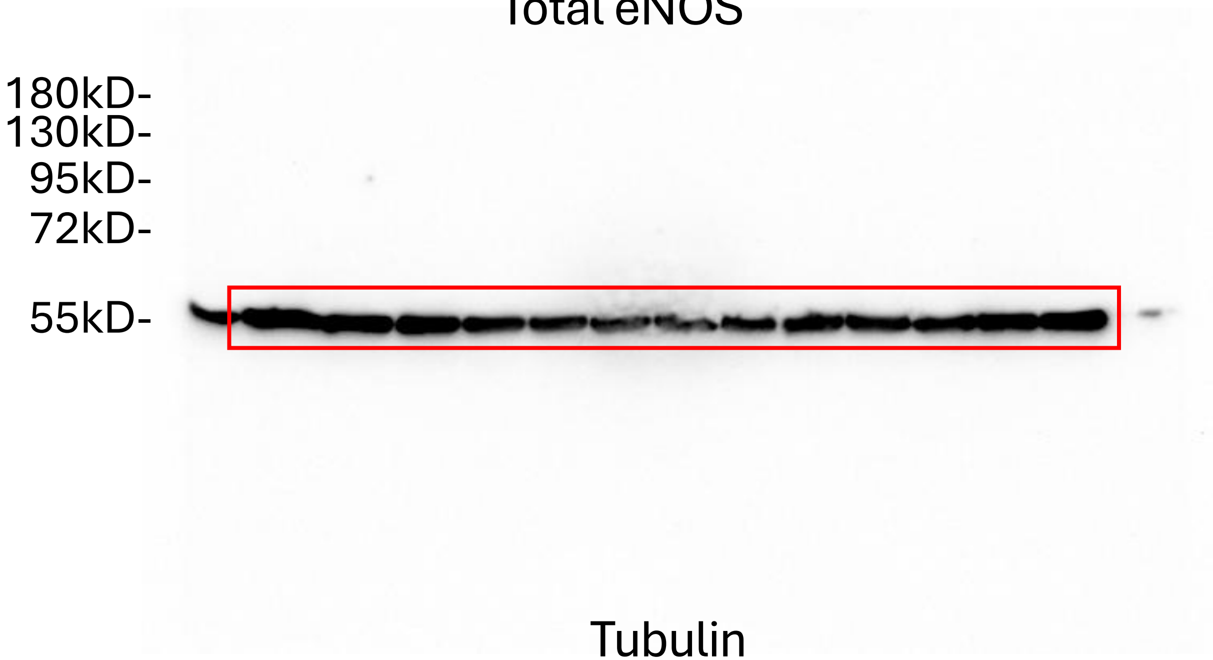

Tubulin

Extended data Figure 5a

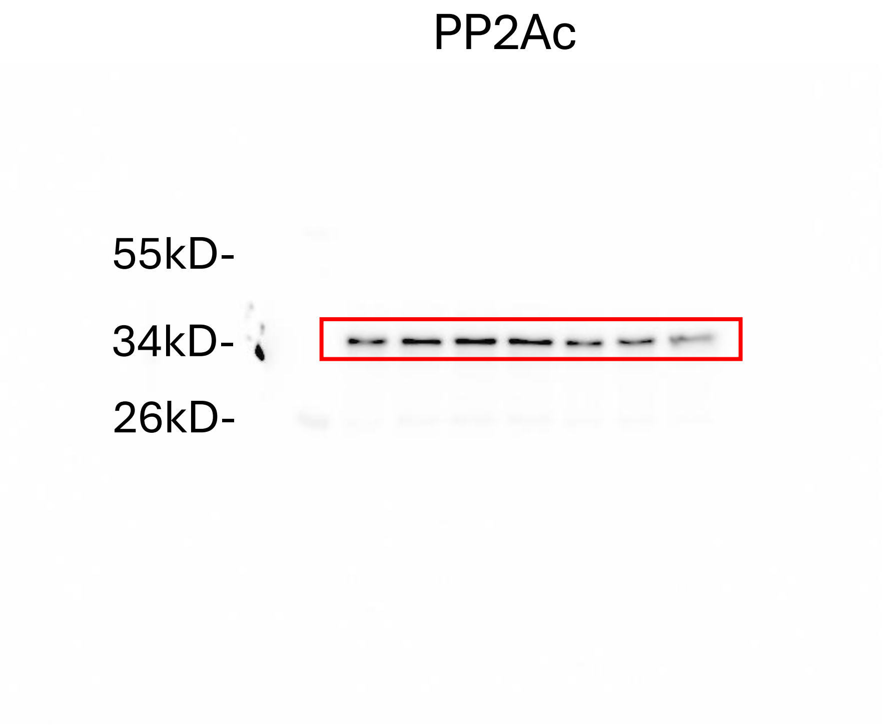

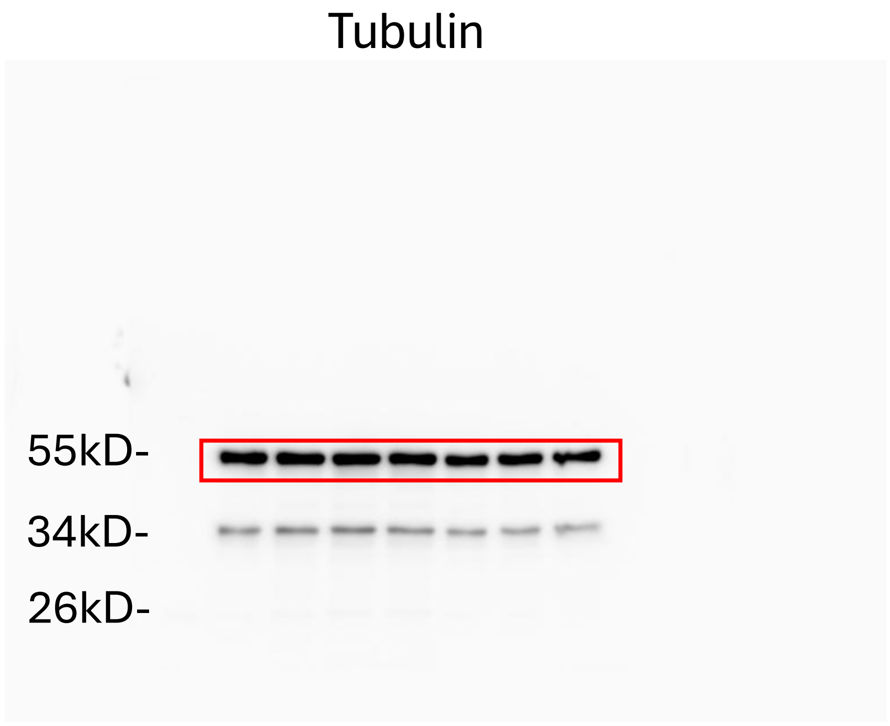

Extended data Figure 5b

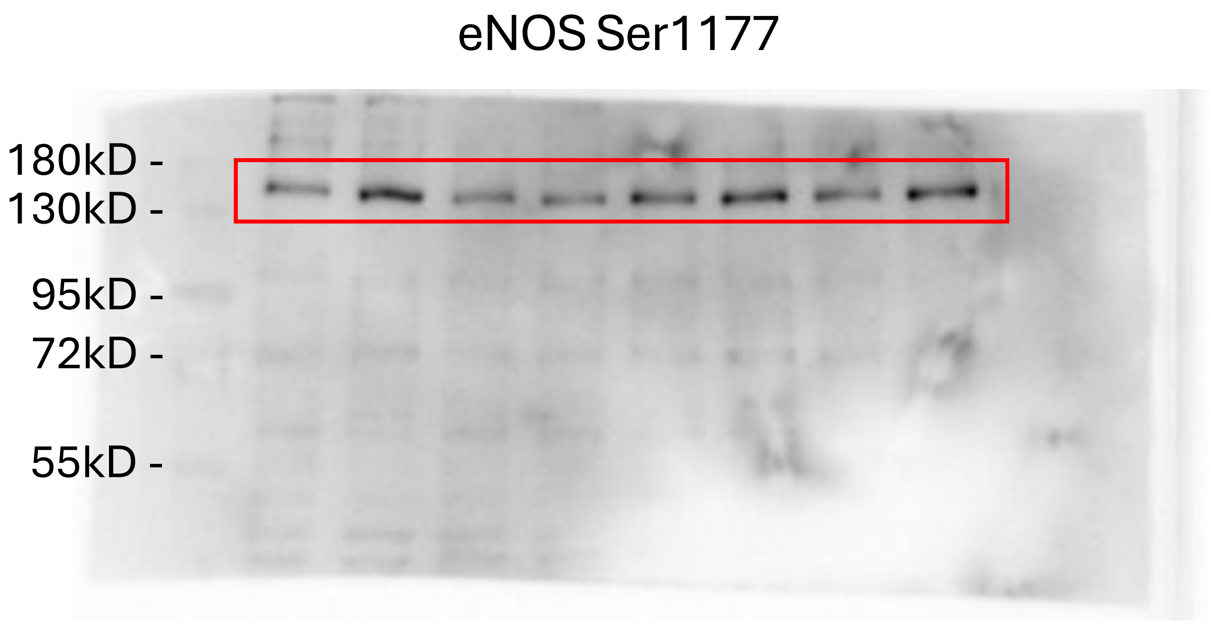

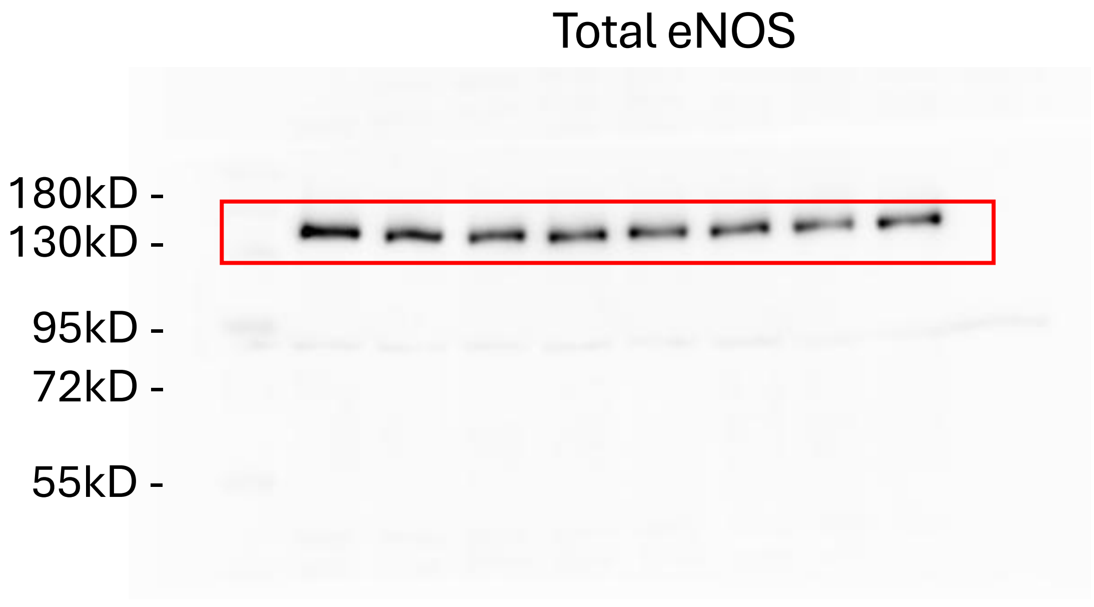

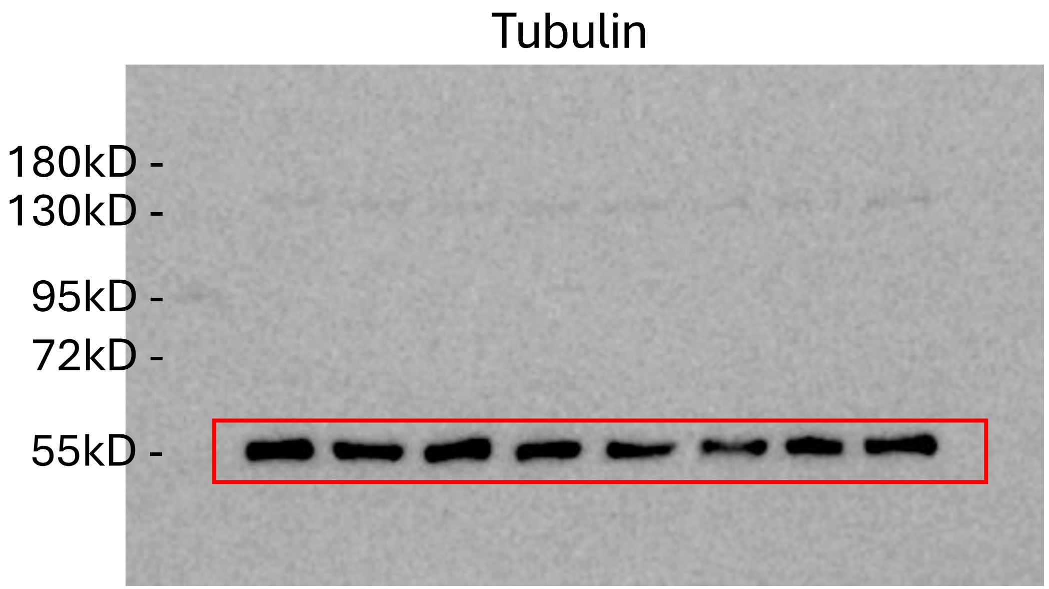

Extended data Figure 5c

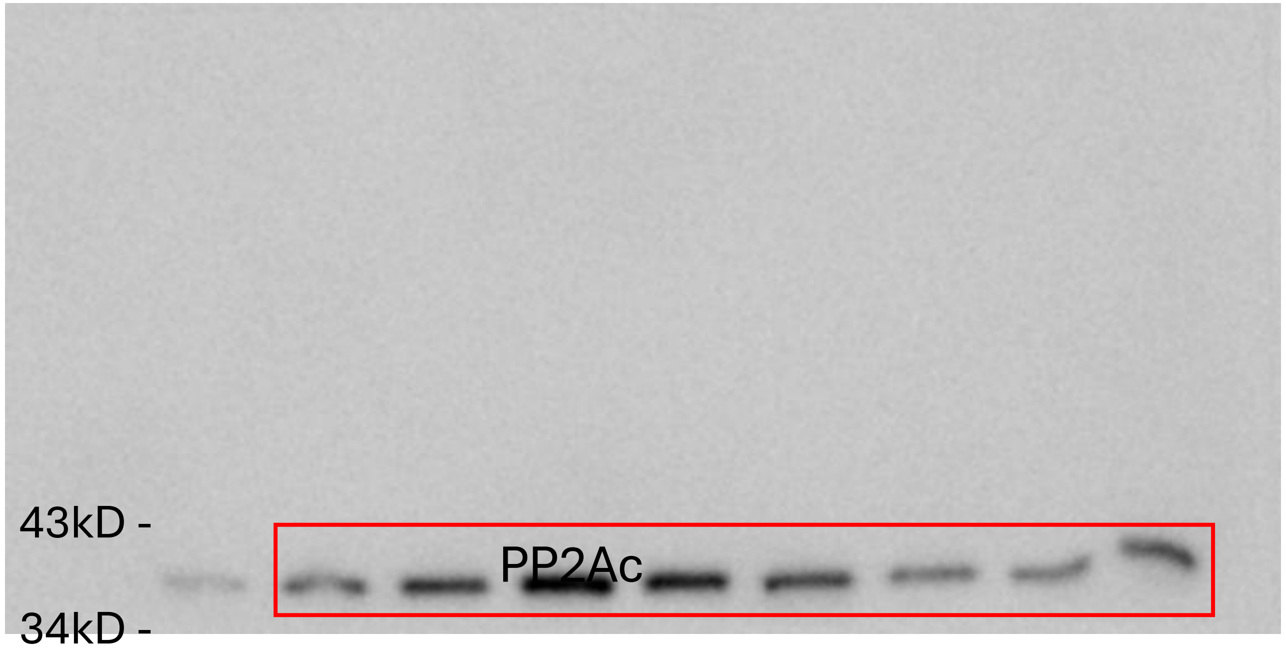

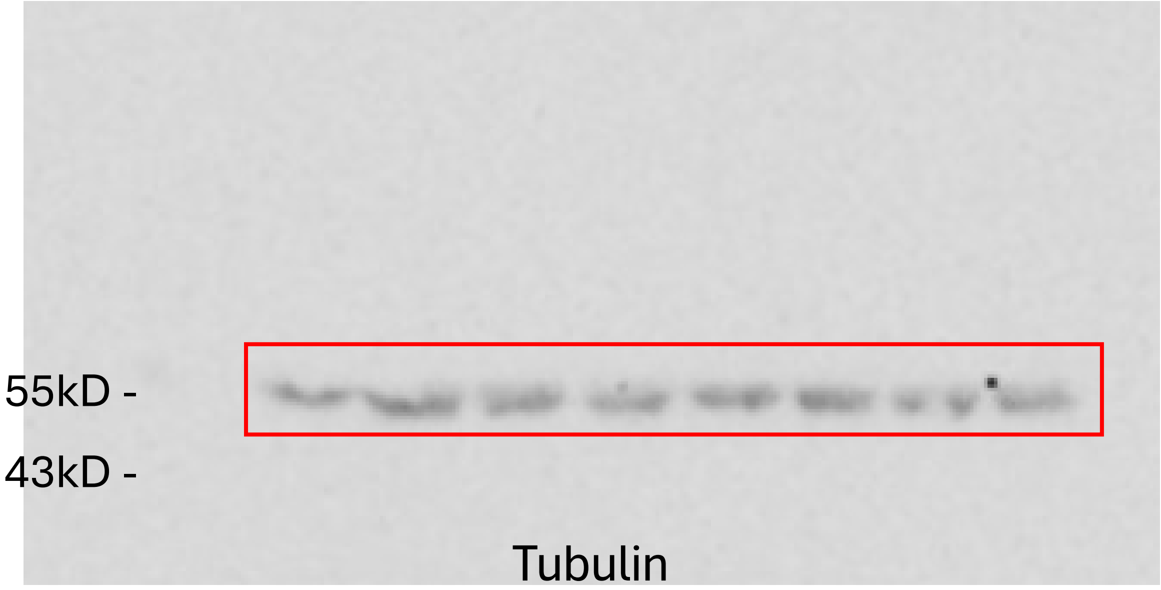

Extended data Figure 5d

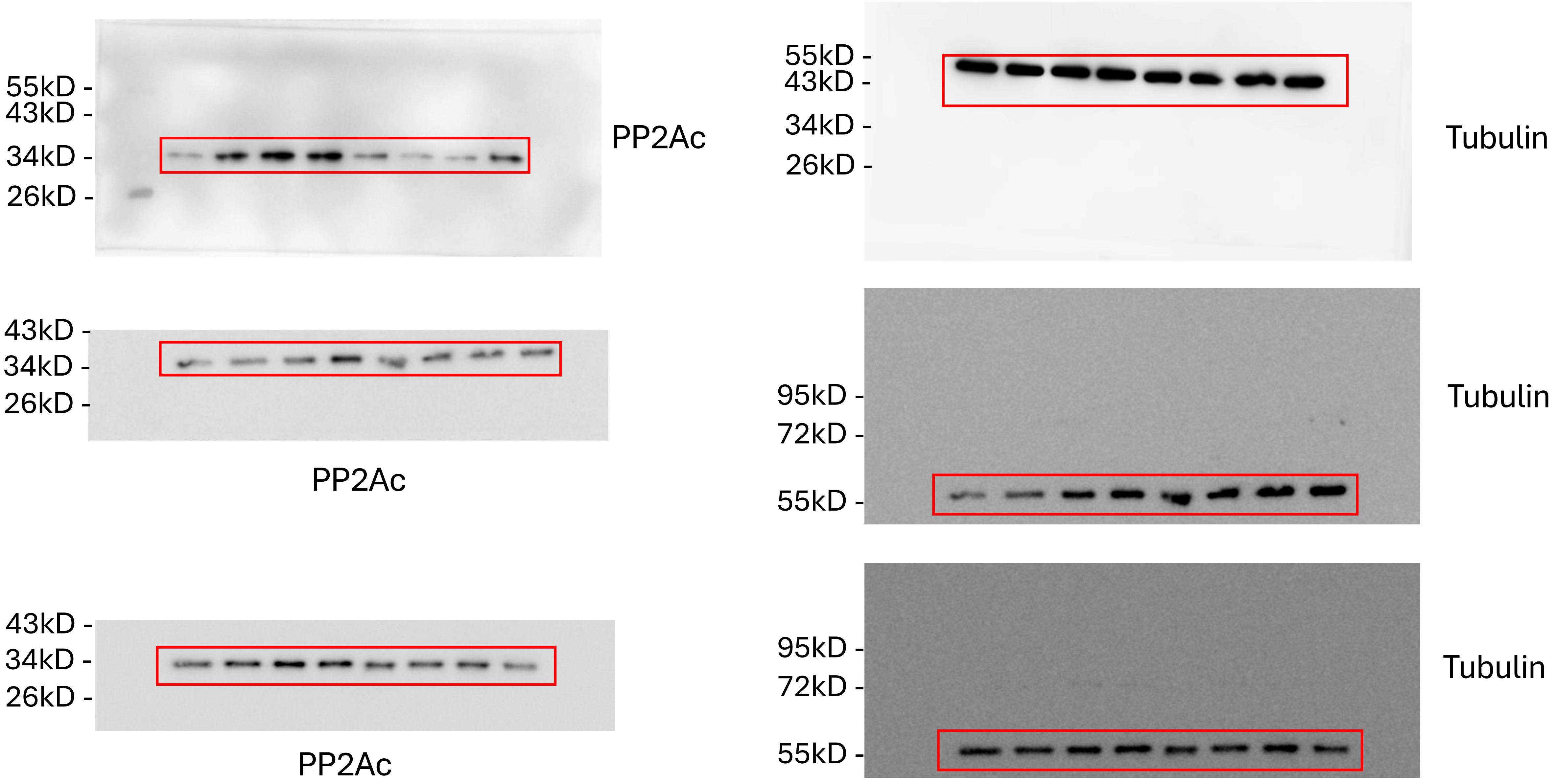

Extended data Figure 5d

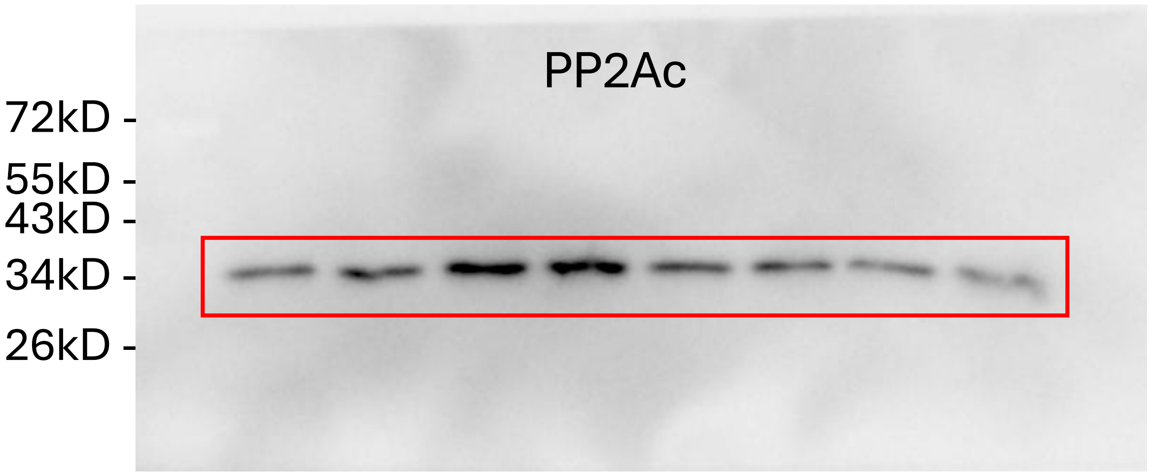

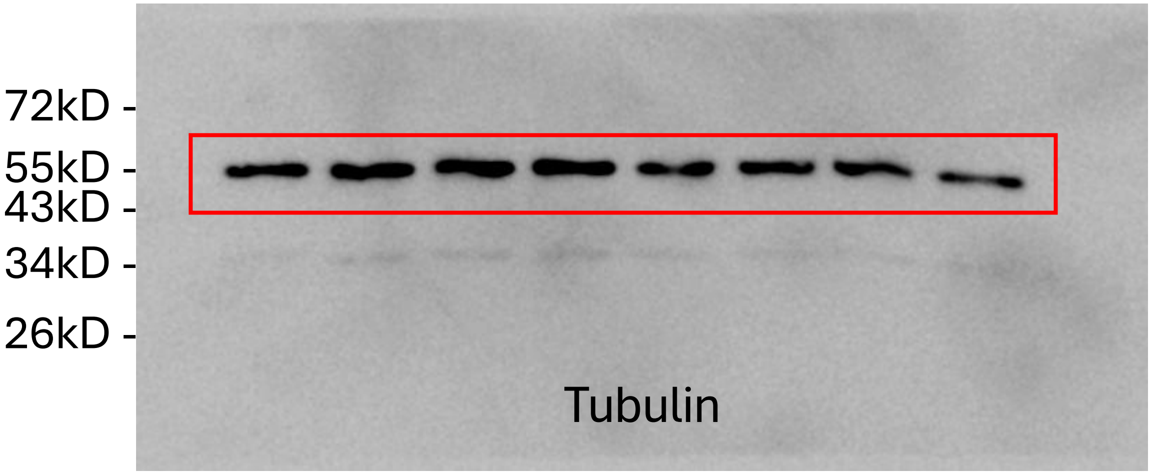

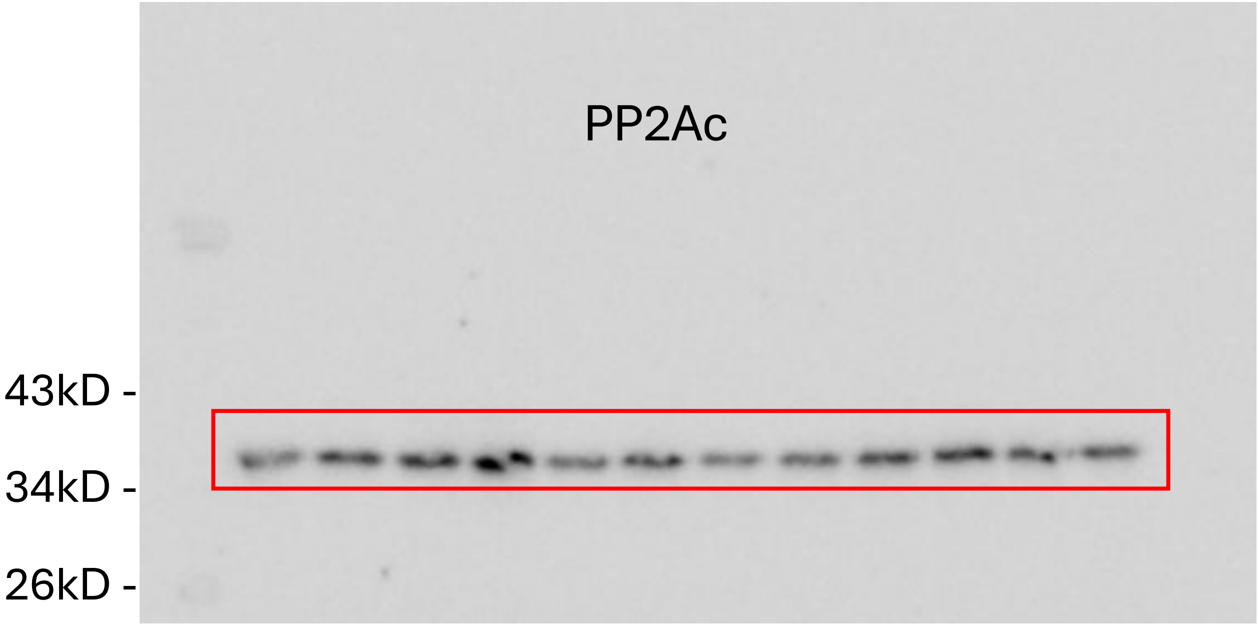

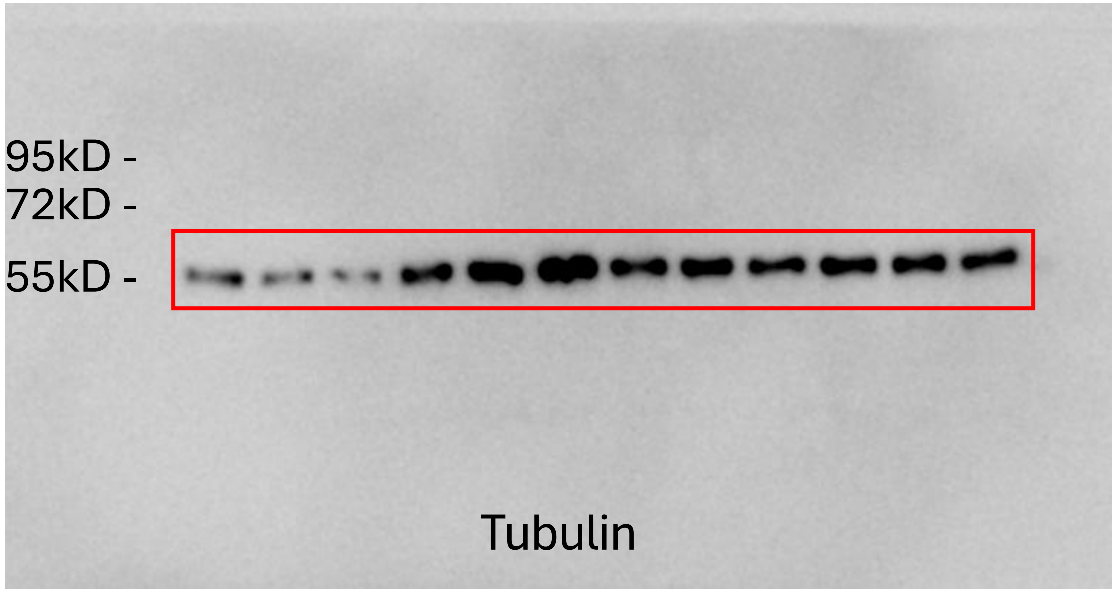

Extended data Figure 6a

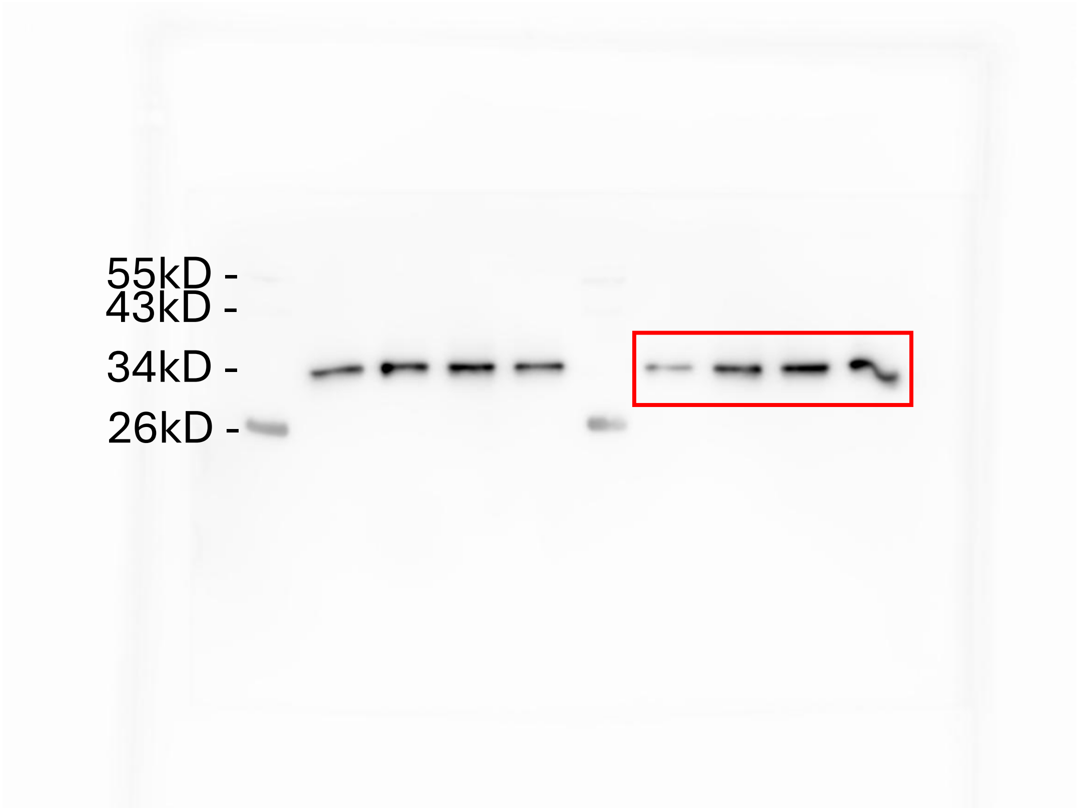

PP2Ac

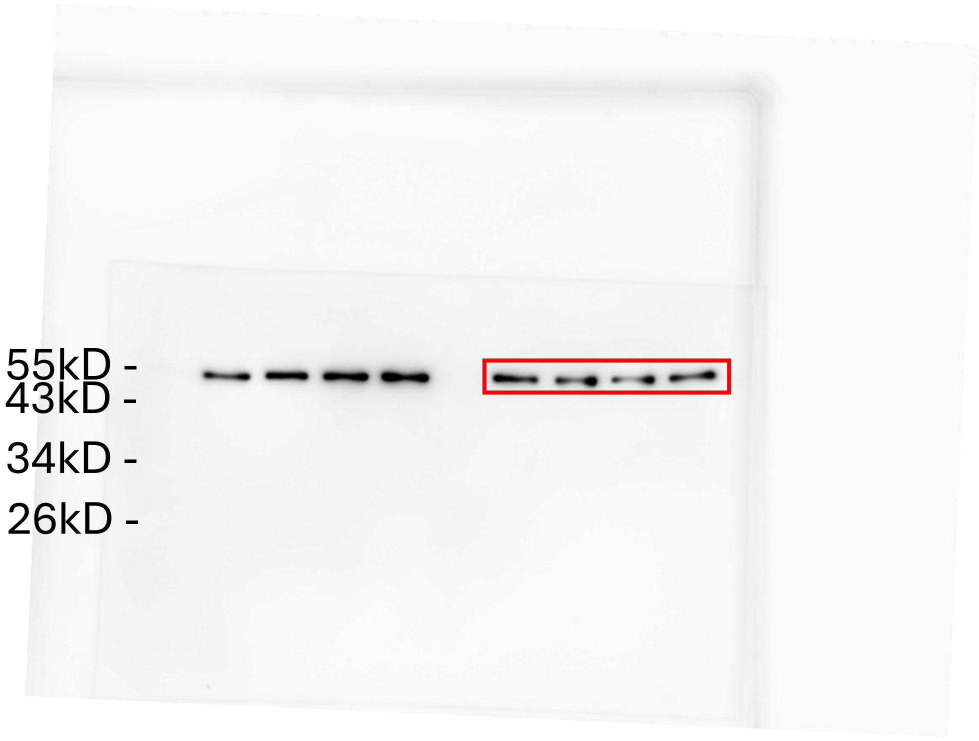

Tubulin

Extended data Figure 6b

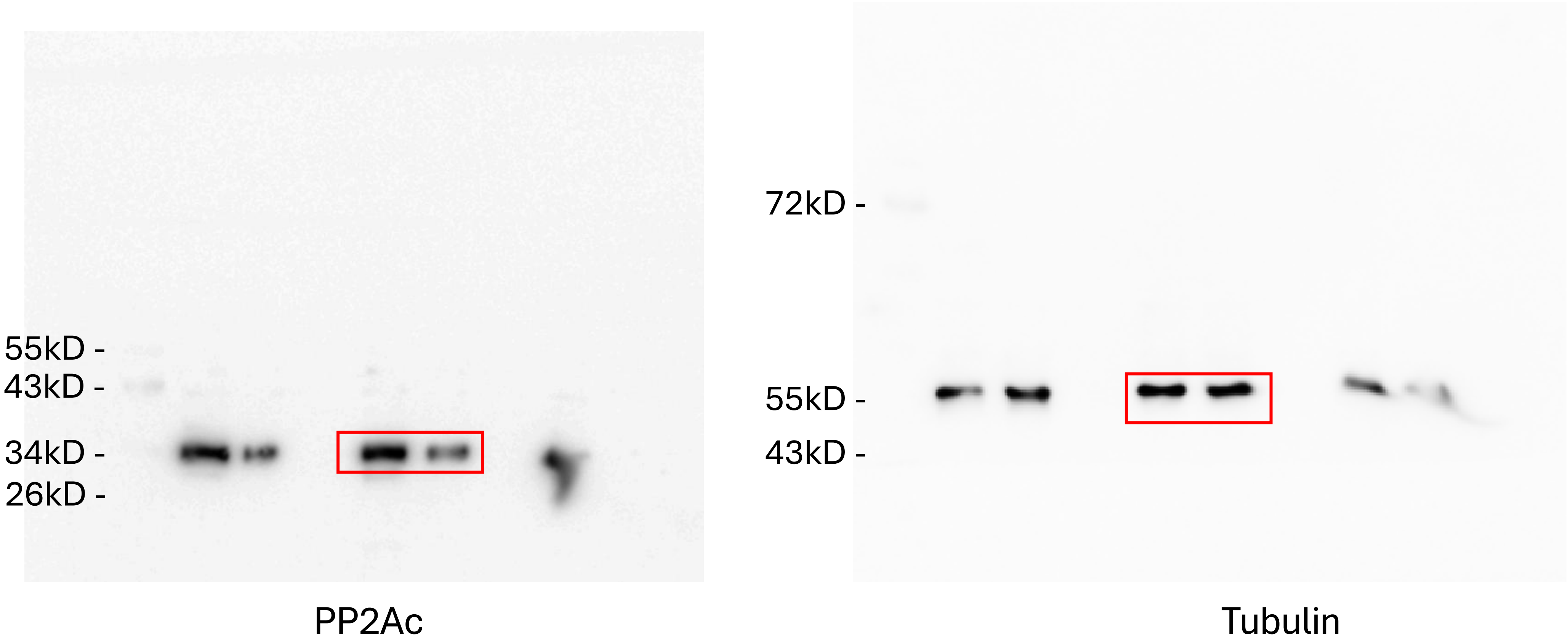

Extended data Figure 6c

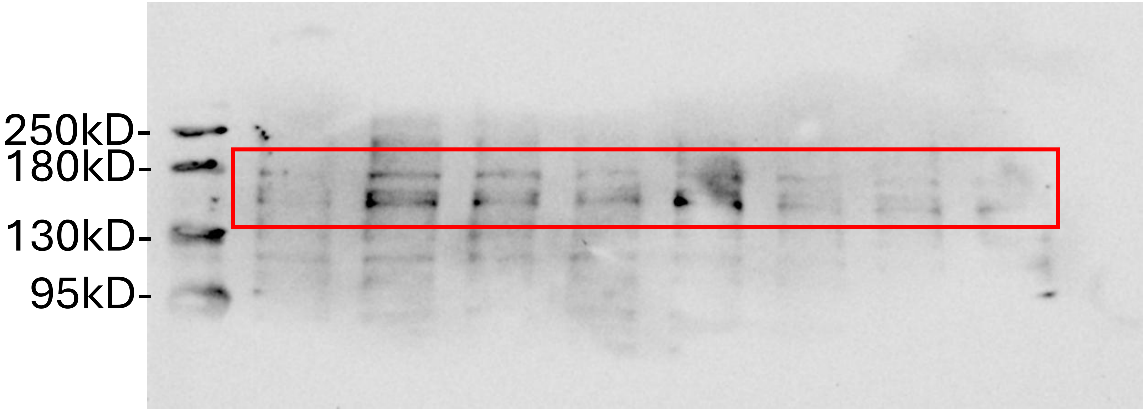

p-EGFR

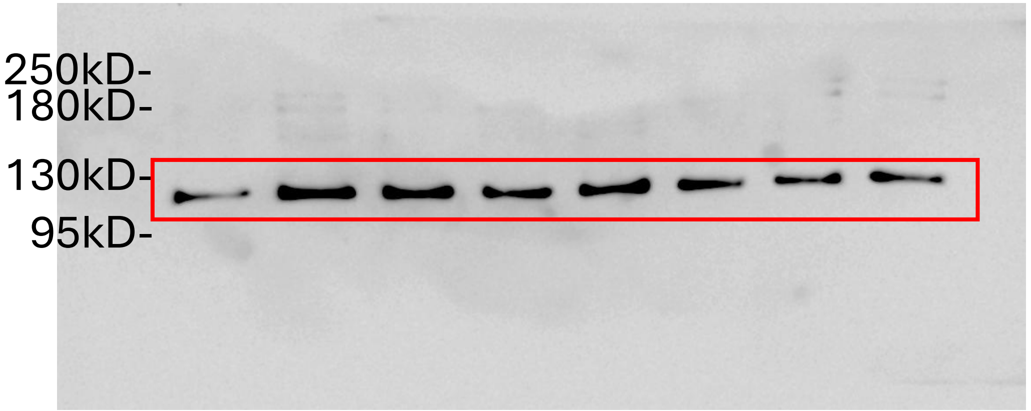

Vinculin

Extended data Figure 6d

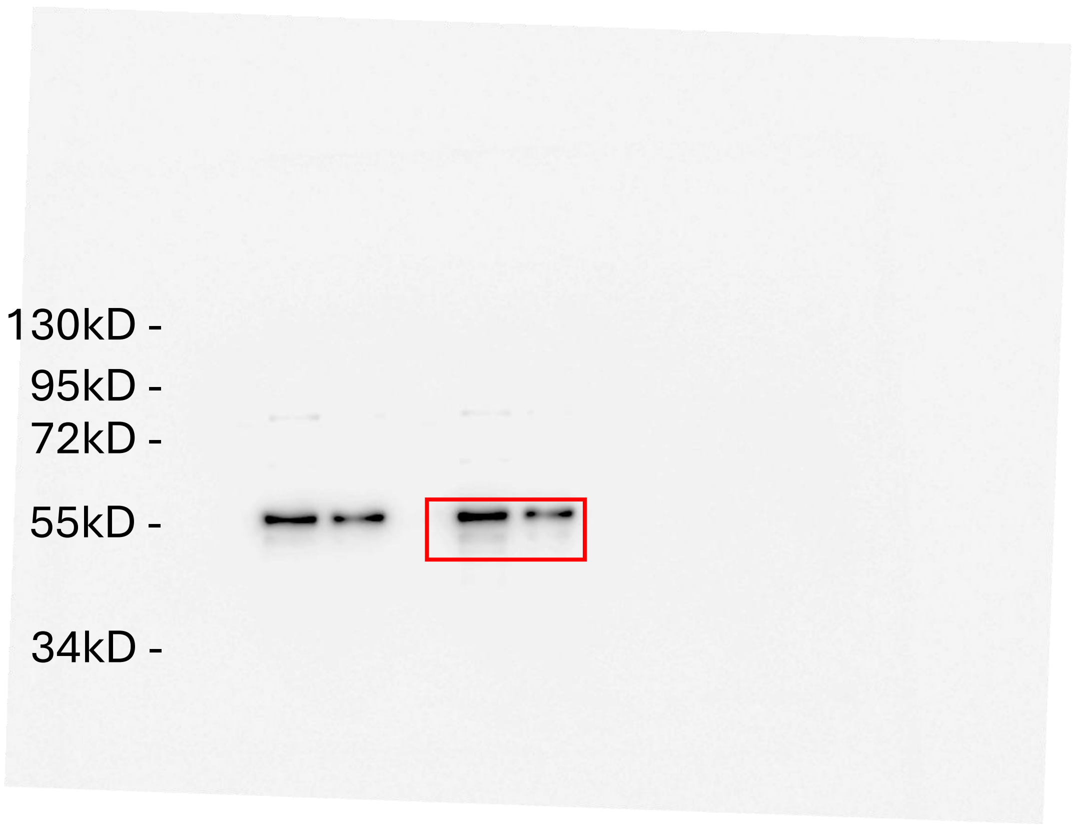

TLR4

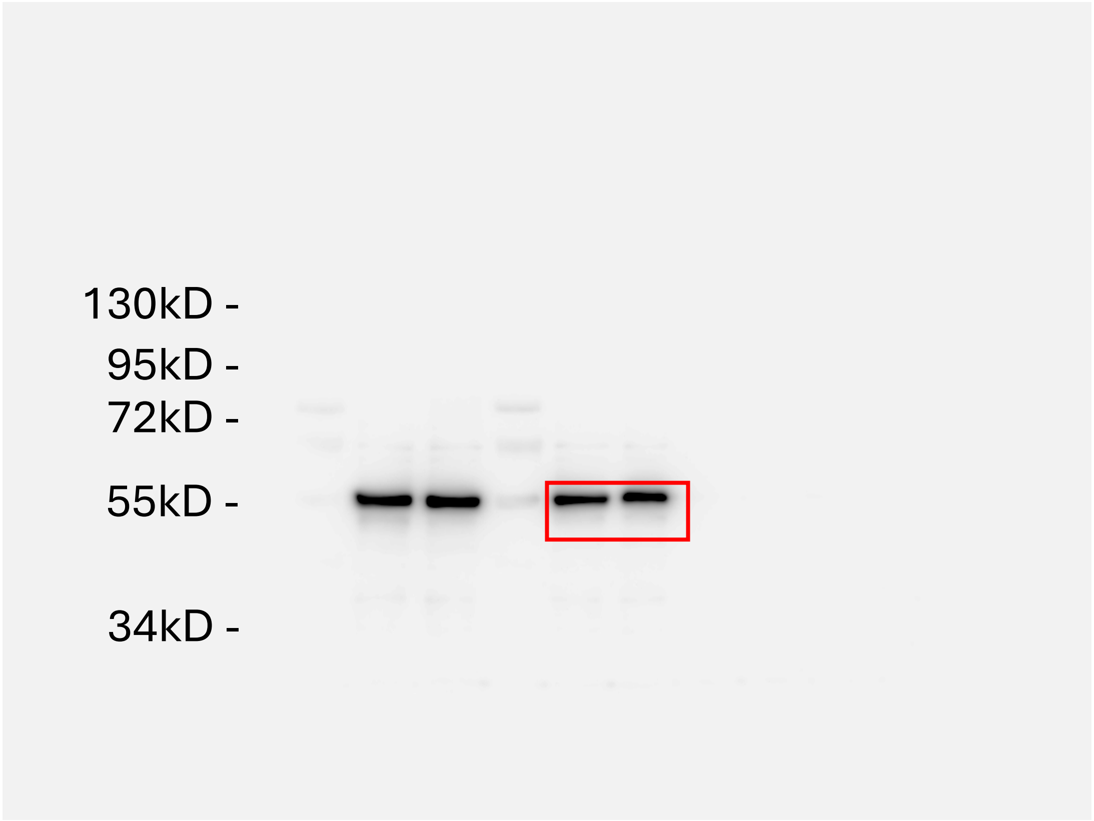

Tubulin

Extended data Figure 6e

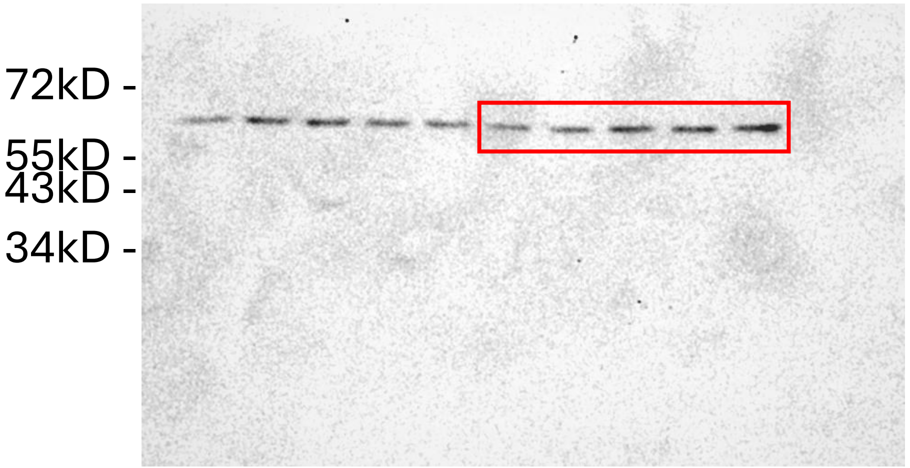

p-AKT

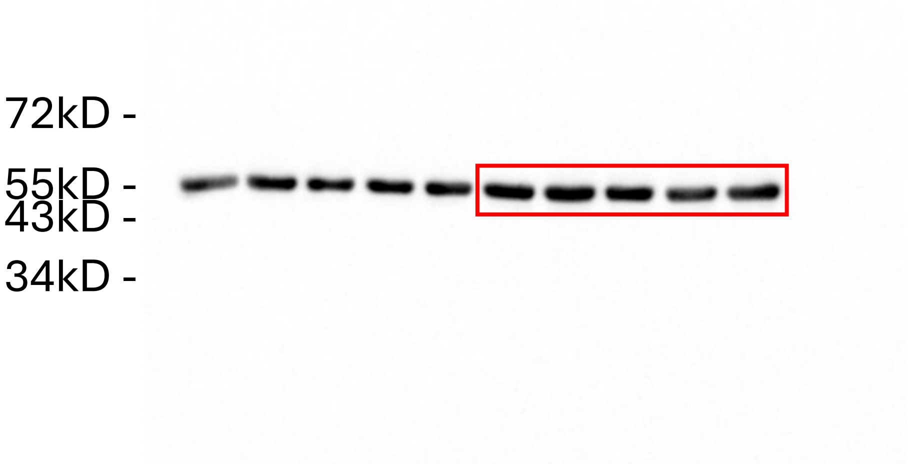

Tubulin

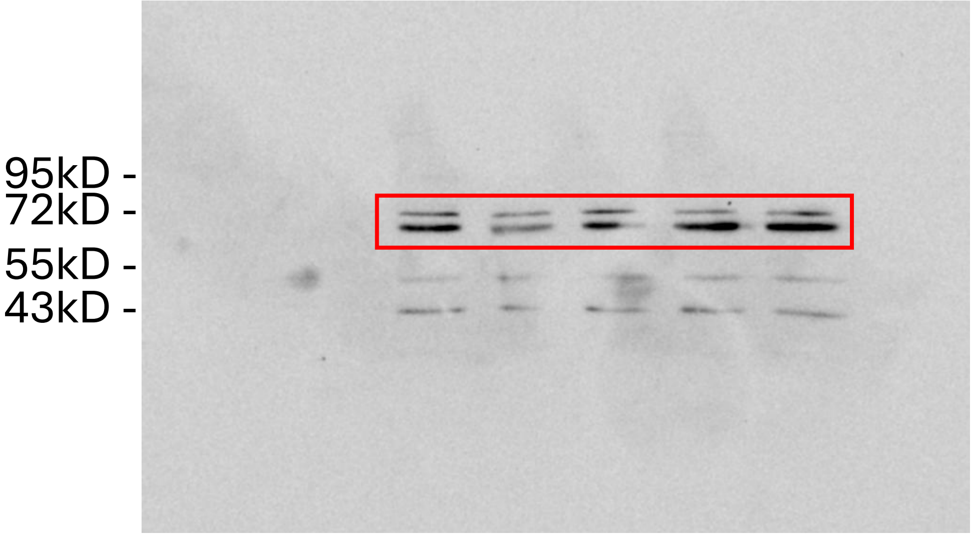

p-PAK4

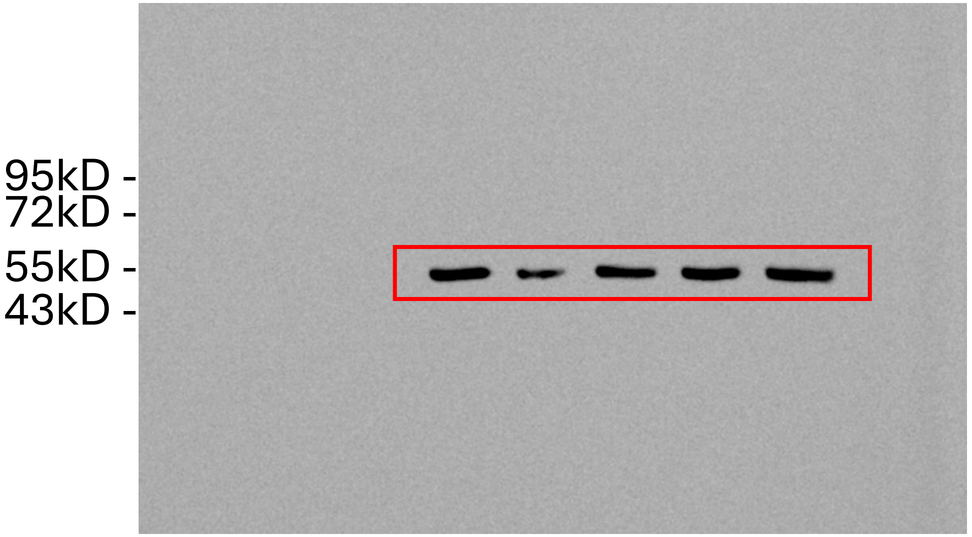

Tubulin

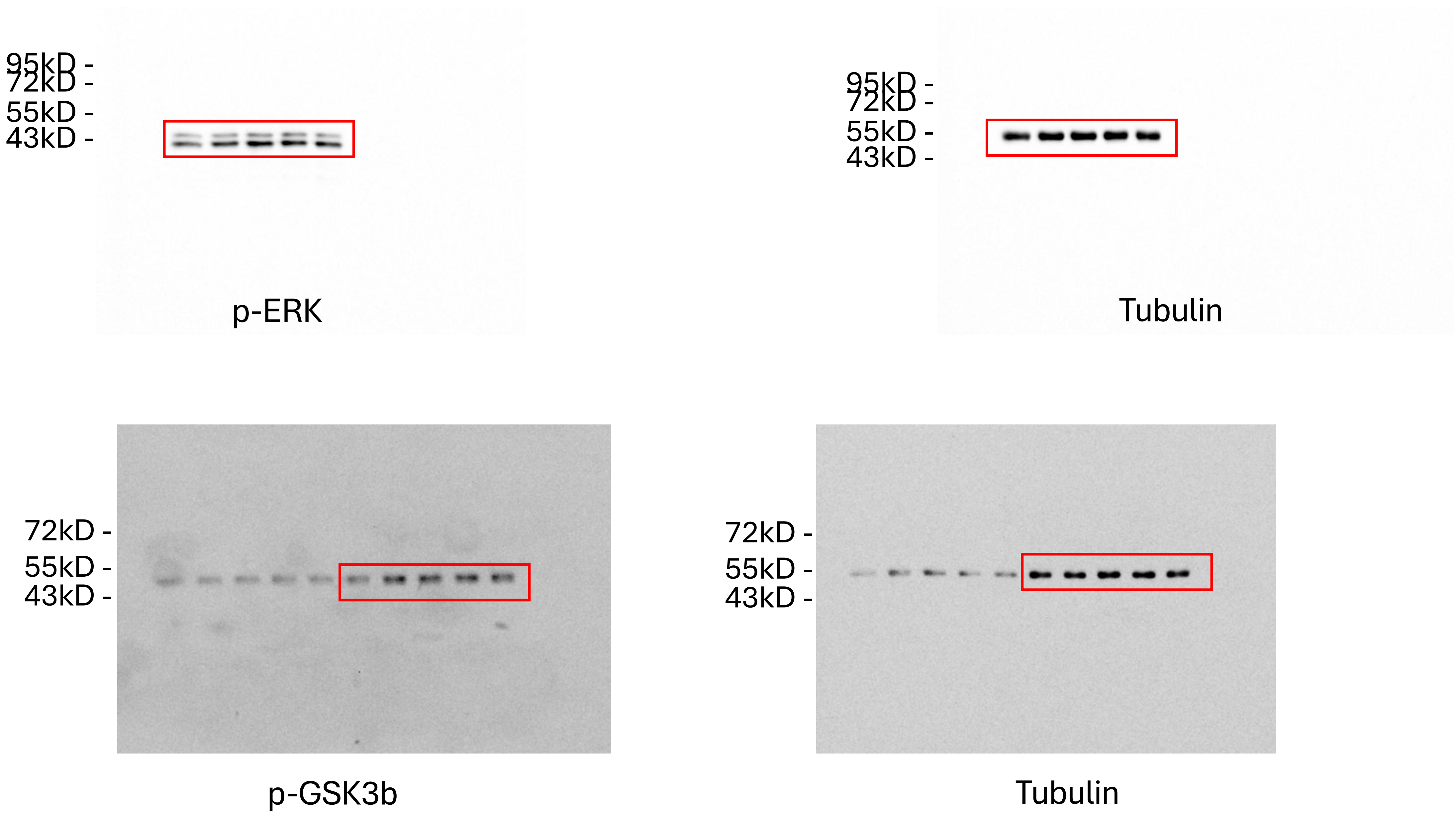
95kD -
72kD -
55kD -
43kD -
p-ERK
95kD -
72kD -
55kD -
43kD -
Tubulin
72kD -
55kD -
43kD -
p-GSK3b
72kD -
55kD -
43kD -
Tubulin

Extended data Figure 7a

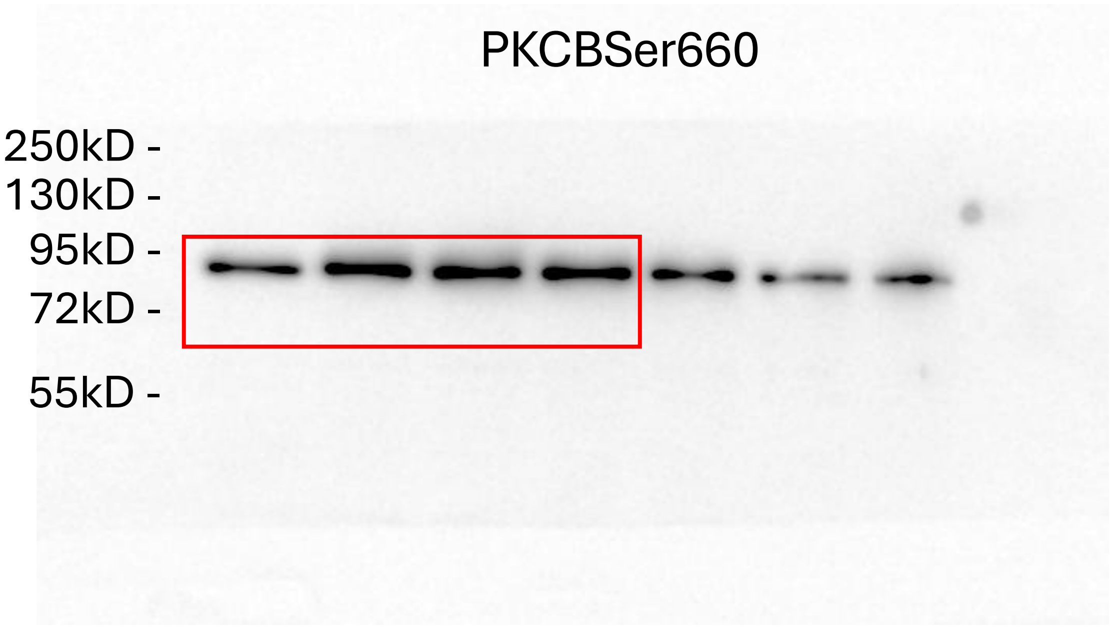

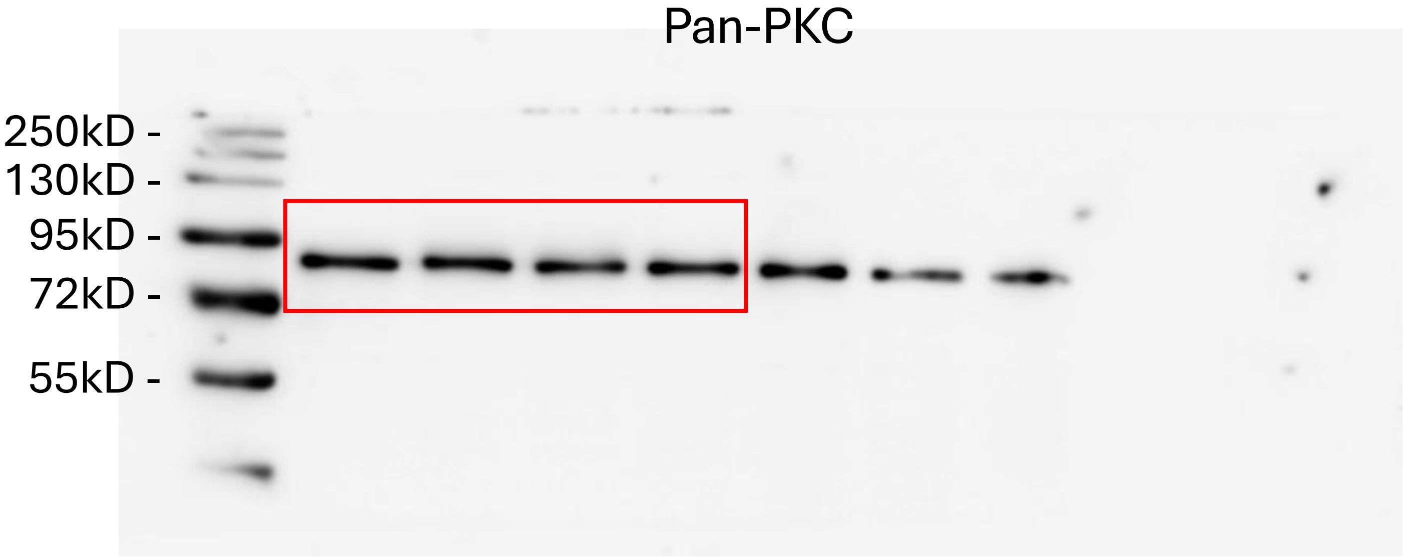

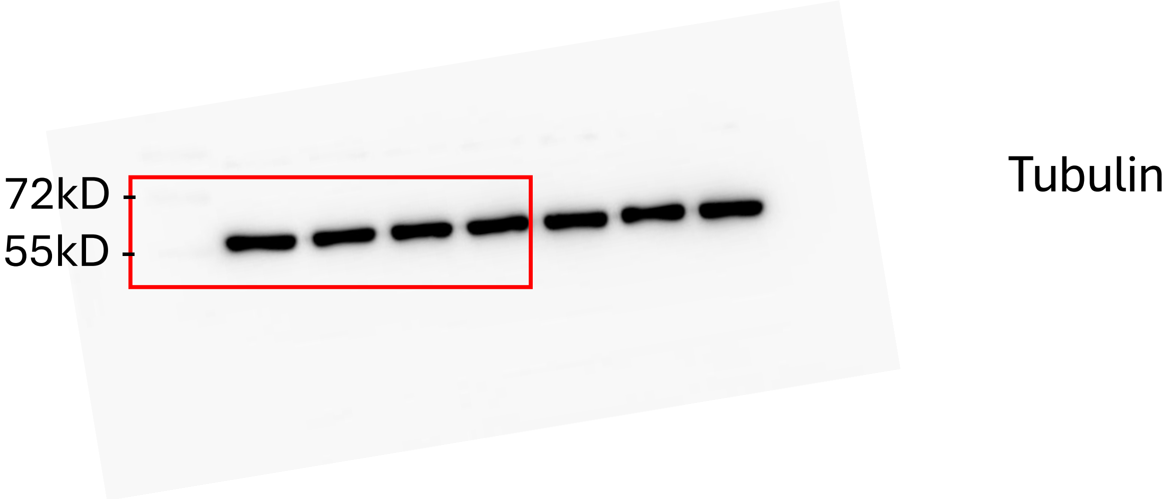

Extended data Figure 7b

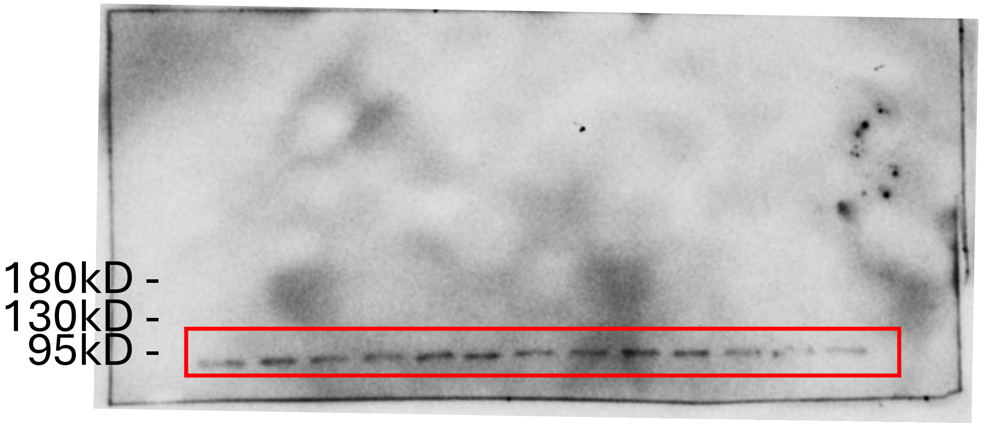

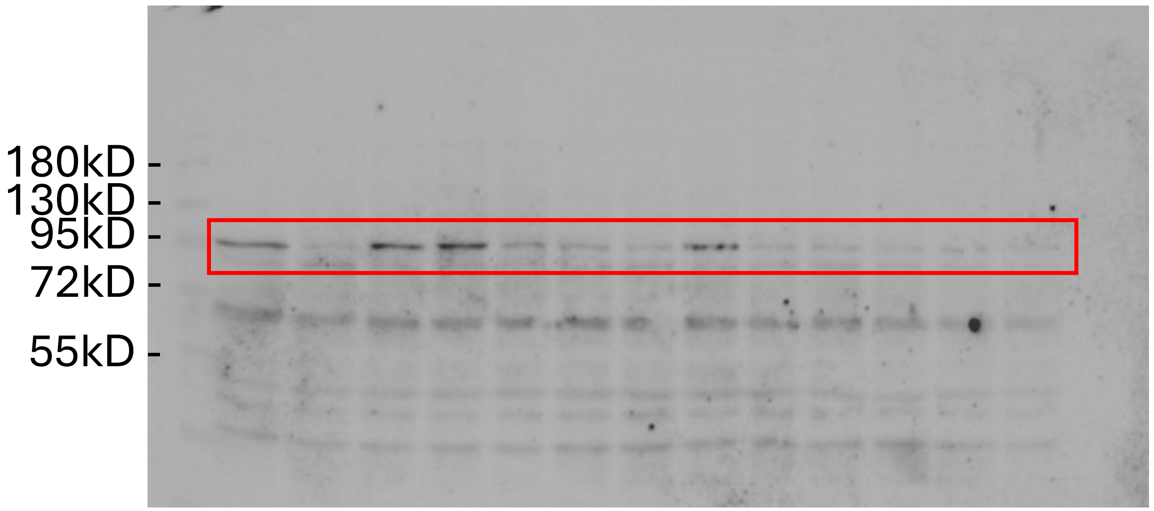

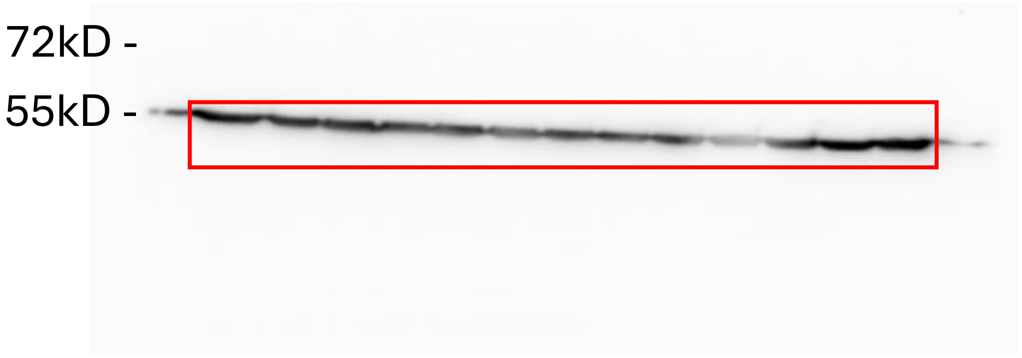

Extended data Figure 7c

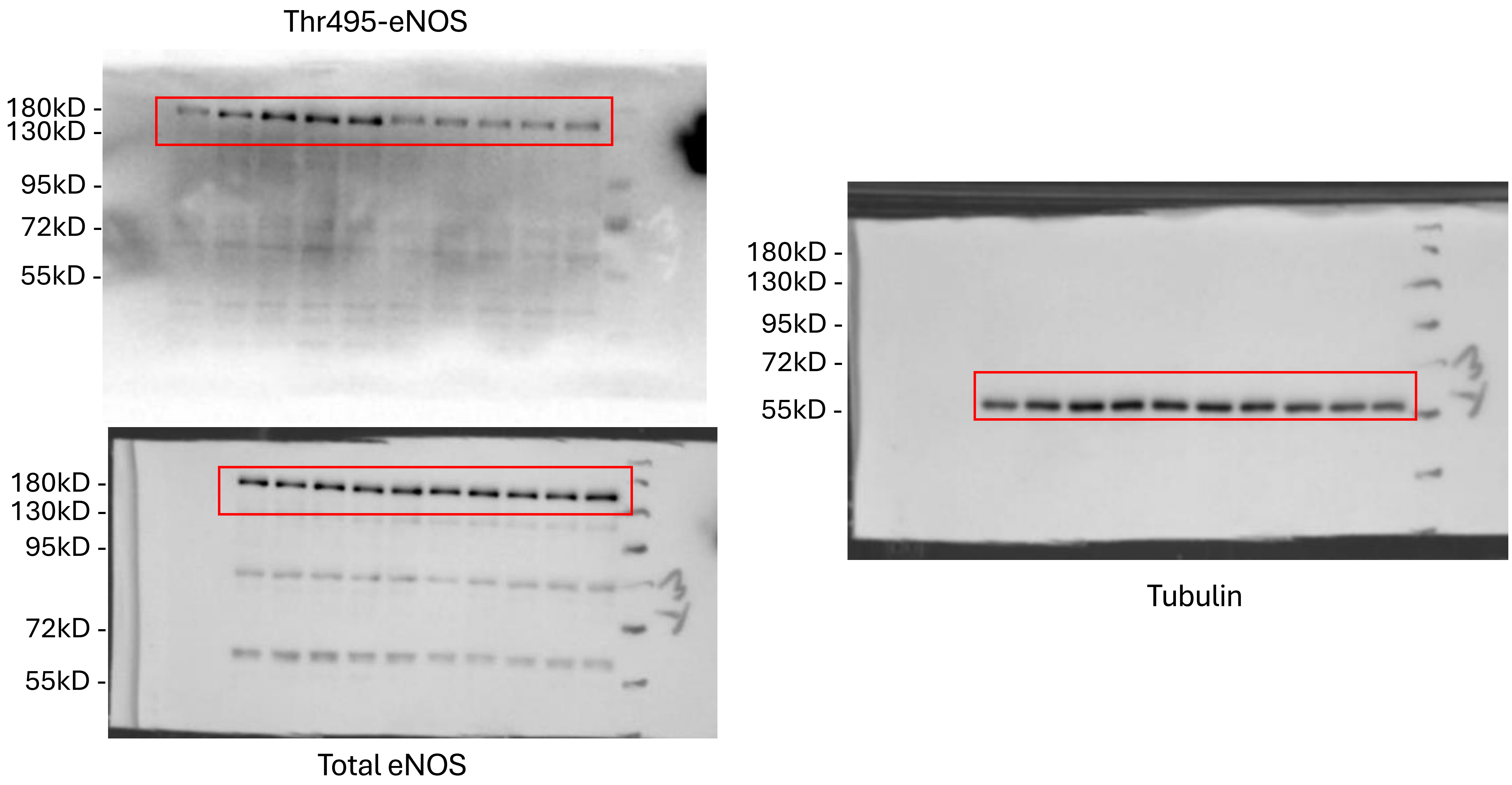

Extended data Figure 7d

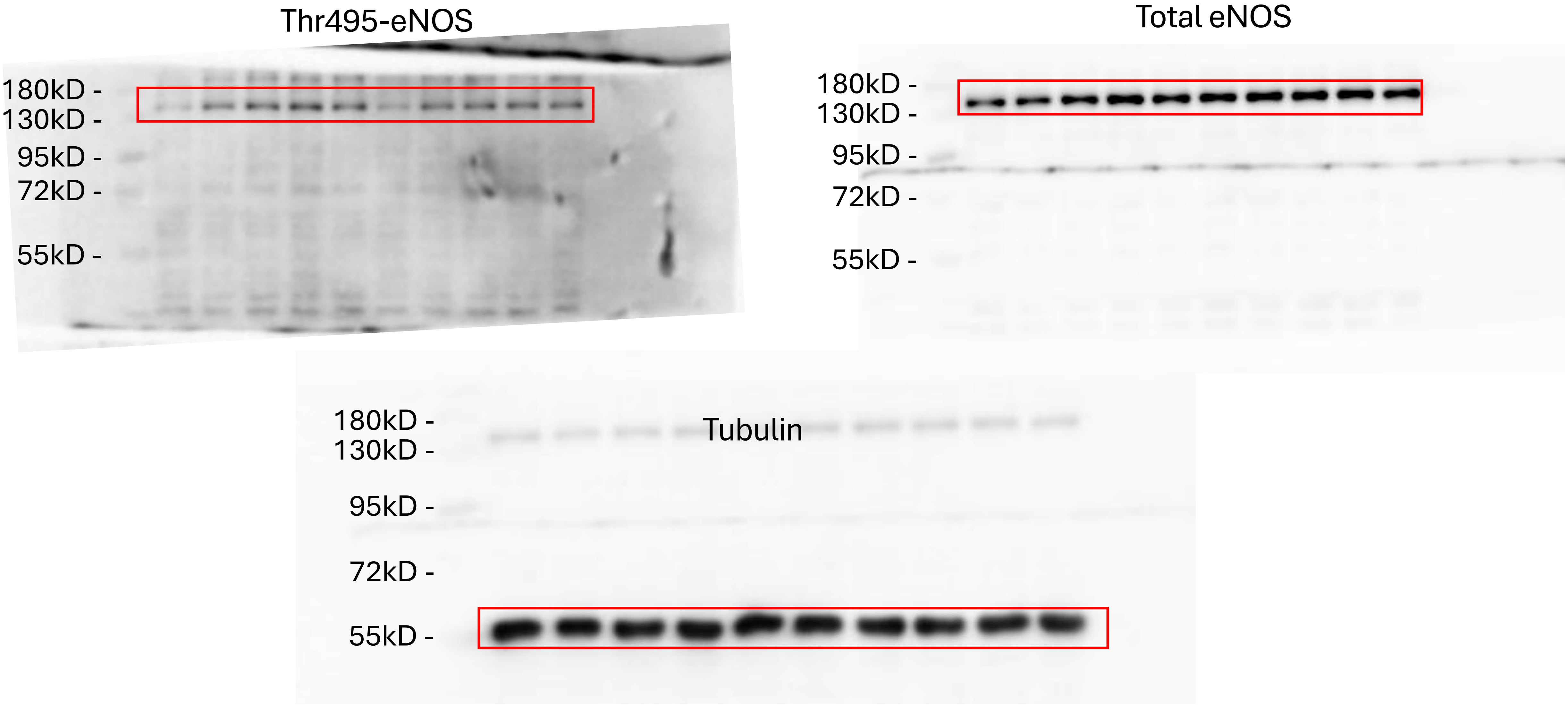

Extended data Figure 7d

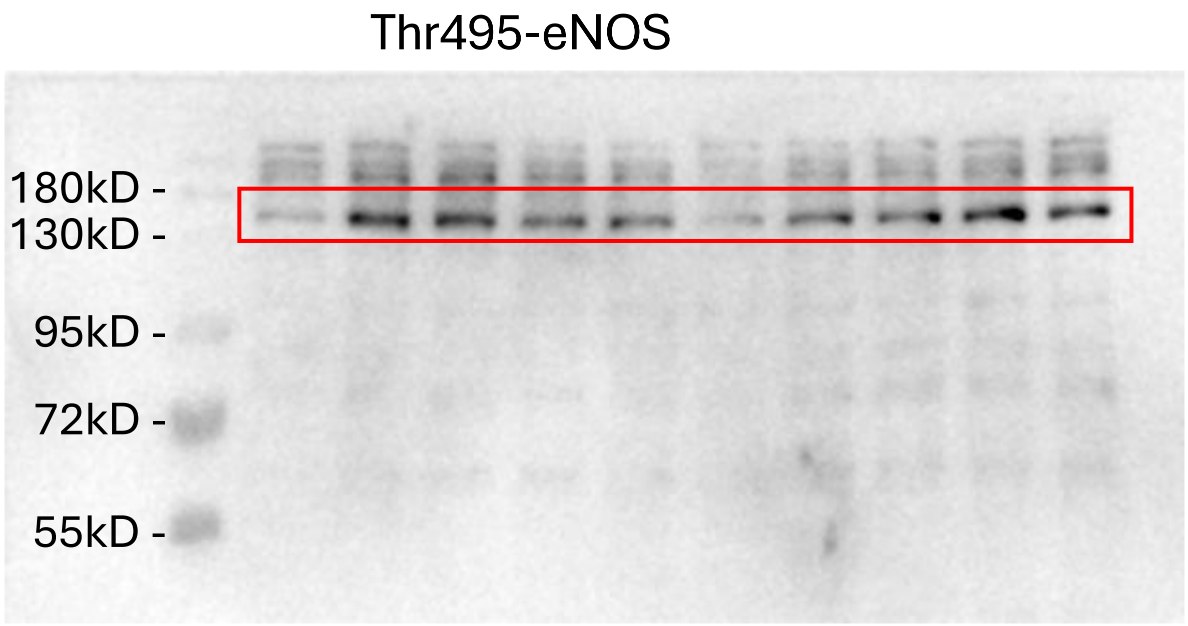

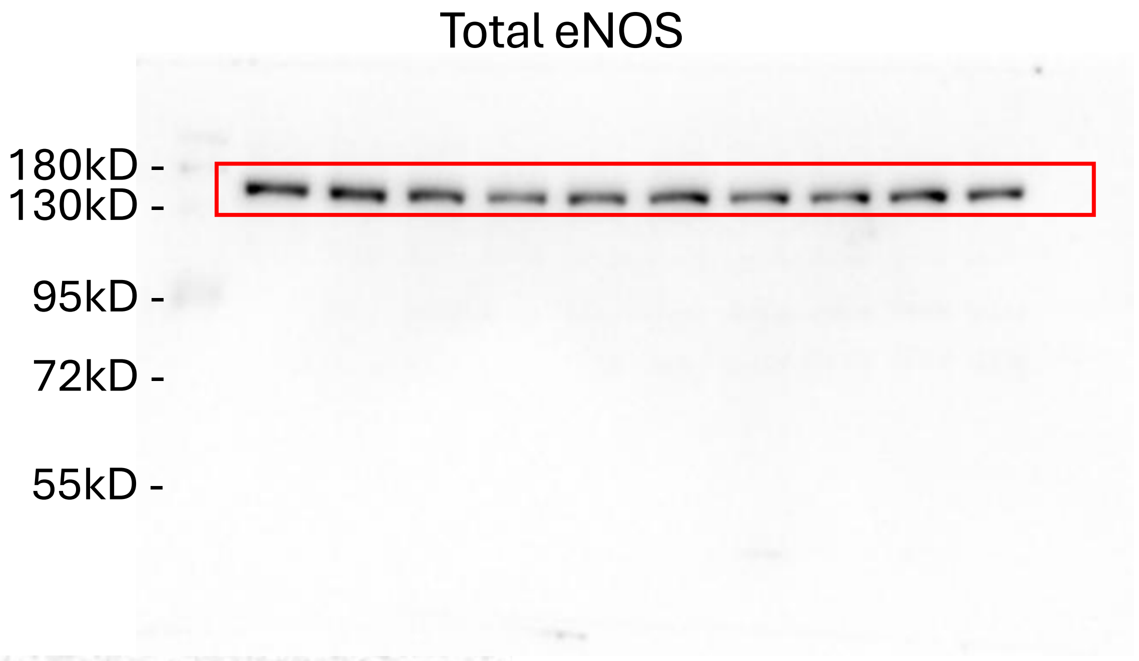

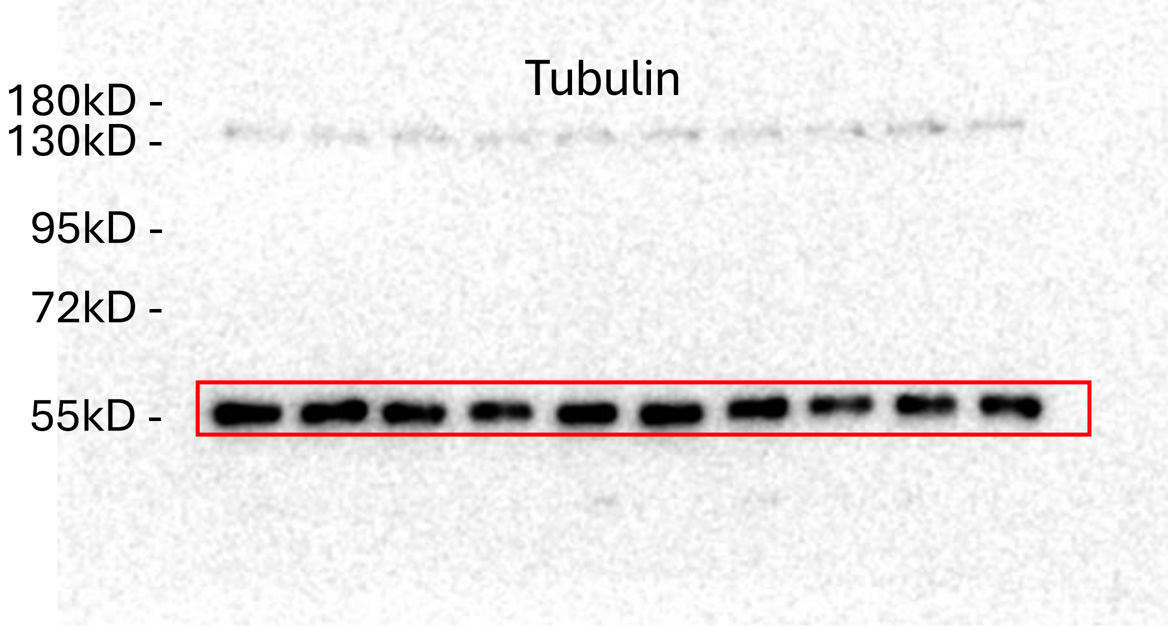

Extended data Figure 7f

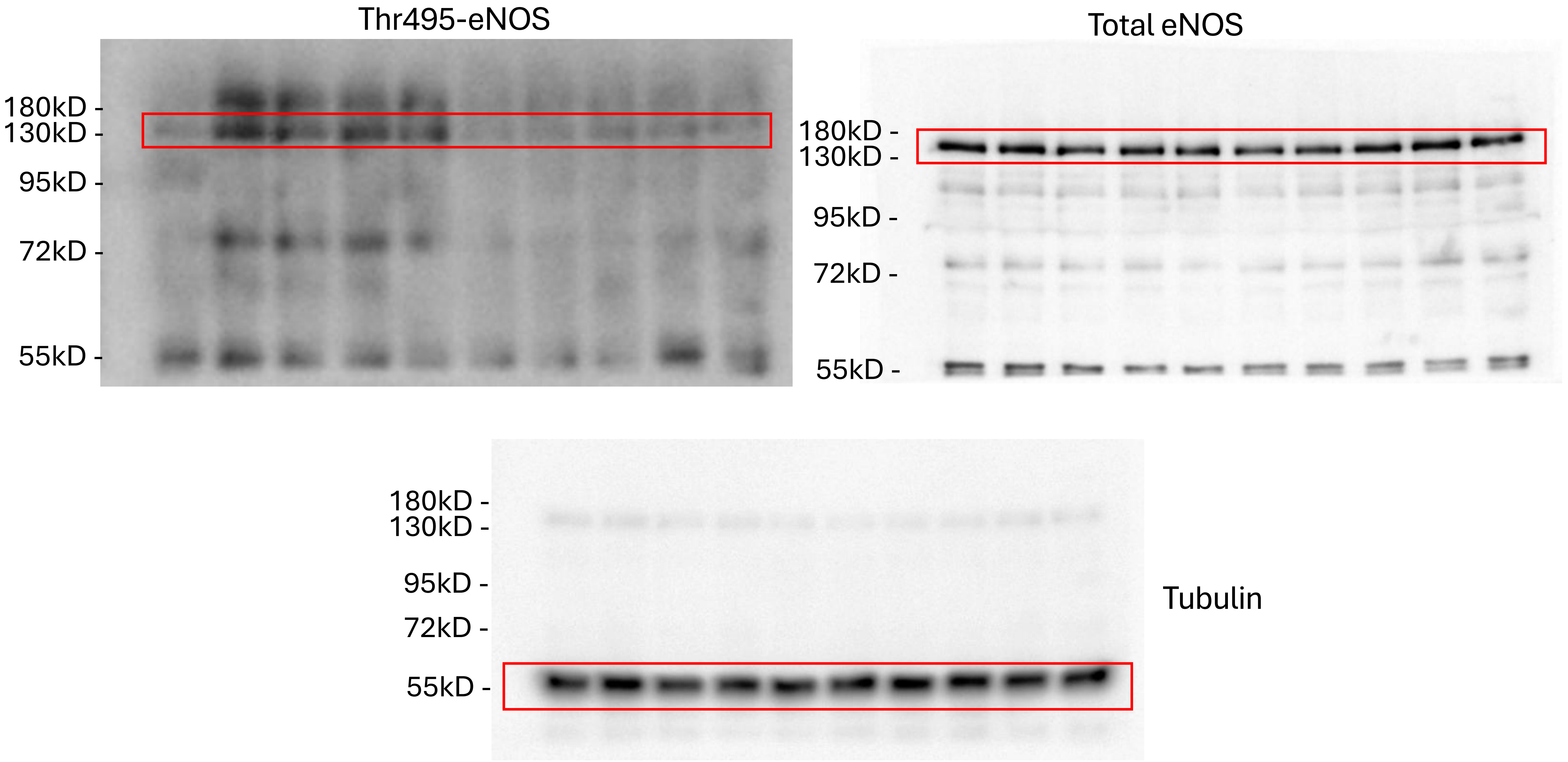

Supplement: Supplementary file 9 — Uncropped western blots. [file 41564_2026_2425_MOESM9_ESM.pdf]
