# Supplementary material for: Klebsiella pneumoniae inhibits vasodilation through capsule and T6SS-dependent pathways
Source: Nat Microbiol. 2026 Jul 21;11(8):2170–86. doi: 10.1038/s41564-026-02425-0 (PMC13423877; doi:10.1038/s41564-026-02425-0)
Supplement: Supplementary file 2 — Peer Review File [file 41564_2026_2425_MOESM2_ESM.pdf]

nature portfolio

Peer Review File

Klebsiella pneumoniae inhibits vasodilation through capsule and T6SS-dependent pathways

Corresponding Author: Professor Jose Bengoechea

Version 0:

Reviewer comments:

Reviewer #1

(Remarks to the Author)
This manuscript addresses a largely understudied aspect of Klebsiella pneumoniae pathogenesis: the bacterium's ability to disrupt vascular function by targeting the endothelial nitric oxide synthase (eNOS), thereby inhibiting agonist-induced vasodilation. While endothelial dysfunction has been extensively examined in the context of viral haemorrhagic infections (e.g., Ebola) and in bacterial sepsis more generally, few studies have dissected K. pneumoniae's direct mechanisms of impairing vascular function.

Overall, I think the study is robust and well-presented and does a good job at analysing the mechanistic aspects of the biology. I feel a few presentation elements might improve reader experience:

Enhanced Timeline Schematics: A diagram illustrating the timeline of infection, addition of agonists, and sample collection in each experiment would help readers better follow the experimental steps.

Statistics and Replicates: Although the paper states the number of biological replicates in multiple sections, consistently emphasising technical replicates (e.g., n=3, each in triplicate) vs. biological replicates (e.g., 3 separate rats per group) would clarify the robustness of some of the data.

The discussion highlights many possible research expansions, but a concise "Future Directions" section could strengthen the discussion.

In addition, it's worth considering a few additional studies to strengthen the potential translational impact:

The ex vivo model is a powerful tool, but the study stops short of verifying this vascular dysfunction in vivo or in other vascular beds (e.g., pulmonary, renal, or cerebral microcirculation):

Testing in an animal infection model (e.g., mice or rats) to measure blood pressure, tissue perfusion, or immune-cell infiltration would confirm physiological relevance. This could be done, perhaps, by comparing wild-type K. pneumoniae versus T6SS/capsule mutants.

It might be insightful to examine if the same T6SS-dependent phenomenon occurs in coronary, renal, or cerebral arteries, especially since (I believe) K. pneumoniae commonly causes disseminated infections.

The study suggests potential avenues for host-targeted therapies, such as PKCβ inhibition or mtROS scavenging, to restore vasodilation:

Evaluating whether in vitro or ex vivo treatments combining bactericidal agents (e.g., colistin or carbapenems) plus a PKCβ inhibitor or MitoTEMPO enhances bacterial clearance or reduces vascular damage might appeal to clinicians.

A fuller characterisation of inhibitor efficacy, toxicity ranges, and potential off-target effects in a more physiologically relevant model could help transition these findings into animal models or early translational studies.

Reviewer #2

(Remarks to the Author)
In this study by Rehman, et al. the authors seek to define mechanisms by which Klebsiella pneumoniae influences vasodilation. Using a creative ex vivo approach utilizing rat tissue, the authors constrict blood vessels with the chemical phenylephrine (PE)

and monitor vessel dilation with acetylcholine (ACh). In this system, they observe that K. pneumoniae inhibits blood vessel re-dilation after the ACh treatment. To further probe this finding, the authors use in vitro infection of human endothelial cells to characterize post-translational modifications within the eNOS pathway that correlate with inhibition of re-dilation. This study addresses vascular biology, an important aspect of K. pneumoniae infection that has not been investigated in detail in previous literature. While the authors should be commended for a mechanistic study about an understudied aspect of K. pneumoniae infection, I am concerned about the relevance of these findings to infection biology in an in vivo context, and I was not fully convinced of the findings surrounding capsule polysaccharide (CPS) limiting phosphorylation of Ser177 in eNOS.
Major Concerns:
1. The authors present an innovative method to monitor blood vessel constriction and dilation in an ex vivo manner. While this should be commended as it avoids the excess use of animals, I am not convinced that this model accurately reflects bacteremia pathology. First, systemic infections include the presence of cytokines, circulating immune cells, and serum which are all important for host-pathogen interactions in bacteremia and work in a coordinated manner. Without these factors present in the ex vivo model, it is difficult to understand whether the observations observed in this study are also present in vivo. Second, during Gram-negative bacteremia, bacteria are rarely found in the bloodstream itself and are quickly sequestered to organs like the liver. Third, the authors should contextualize their findings considering other K. pneumoniae bacteremia pathogenesis work. In a recent study (PMID37463183), the authors used transposon sequencing in ATCC43816 (the same strain used in the present study) to identify K. pneumoniae factors important for survival during in vivo bloodstream infection. In that study, all T6SS genes were dispensable for infection. Thus, although the data in Figure 2 is compelling that the T6SS influences vasodilation ex vivo, due to this pre-existing study, I am not sure that the T6SS specifically impacts K. pneumoniae pathogenicity in the bloodstream. To address this, the author could consider competing their T6SS mutant against WT K. pneumoniae in an intravascular model of bacteremia to demonstrate that the T6SS mutant is less fit during infection.
2. The conclusion from Figure 4 is unclear. The authors suggest that Kp43816 inhibits Ser177 phosphorylation that is induced by eNOS agonists, but this is not apparent by eye. From the raw blot in 4B, it appears that the entire gel lane for the uninfected wells may be darker than the lanes in the Kp43816 lanes, which would make the densitometry calculation in 4B appear higher in the uninfected wells (since the numerator would be a higher value due to a darker lane). These results seem very subtle, and by eye they are undetectable. I am not convinced that this conclusion is supported by the data without a secondary means of confirmation such as knockout cell lines or a reporter assay.
3. Why did the authors choose the timepoints that are used in this study? The results in this study are shown over several hours, but did the authors consider a more prolonged timepoint to see if protein modifications are more dramatically altered later during infection? For example, can the T6SS initiate signaling within minutes? Is it possible that T6SS mutants would be able to influence post translational modifications at longer timepoints, like 24 hours?
Minor Concerns:
1. In Line 154, please update the phrasing to say that “inhibition of validation is observed across multiple diverse strains of K. pneumoniae.” The authors could also consider noting that the different strains of K. pneumoniae produce very different effects in this figure. Thus, some K. pneumoniae strains may inhibit dilation to a higher degree than others, which is an interesting finding.
2. The results of Panel 3D are statistically significant but very subtle. Can the authors consider adding lines for pairwise connection of trials to demonstrate a consistent increase in Ca^{2+} response during each repeat?

Reviewer #3

(Remarks to the Author)
This manuscript describes the effects of infection of endothelial cells with K. pneumoniae on endothelium dependent vasodilation and on signal transduction pathways that appear to mediate inhibition of endothelium-dependent vasodilation using cultured human endothelial cells. Overall this is a well conducted study that clearly outlines the effects of K. pneumoniae infection on endothelium dependent vasodilation and identification of the signaling pathways involved in this inhibition. I only have two significant concerns. The first relates the the authors statement that K. pneumoniae infection has no effect on endothelium apparently based solely on histological examination of vessel longitudinal sections. It is not clear to this reviewer that it is possible to assess, for example, the Barries function of the endothelium using this simple approach. It is suggested that a more rigorous approach be utilized to assessed endothelial structural integrity, or that this statement be softened. Second, while the authors clearly show that K. pneumoniae infection blunts EDH mediated dilation and that Mito-ROS is involved, they have not shown how this blunting is accomplished. Does K. pneumoniae infection reduce dilation mediated by NS-319 or other IKCa/SKCa agonist or IKCa and/or SKCa currents in isolated endothelia cells? Such findings would better "close the loop" for this aspect of the authors findings.

Reviewer #4

(Remarks to the Author)
1. Summary of the Study
In this study, the authors establish a robust ex-vivo vascular model to investigate how Klebsiella pneumoniae infection affects vasodilation. Using rat mesenteric arteries and human endothelial cells, they show that K. pneumoniae impairs acetylcholine-induced vasodilation by targeting nitric oxide (NO) and endothelium-derived hyperpolarization (EDH) pathways. Mechanistically, they identify two parallel bacterial strategies: (1) the trans-kingdom T6SS effector VgrG4 induces mitochondrial ROS (mtROS) in an NLRX1-dependent manner, activating PKCβ and phosphorylating the inhibitory Thr495 site of eNOS, and (2) the capsular polysaccharide (CPS) activates an EGFR-dependent signaling cascade, leading to PP2Ac upregulation and dephosphorylation of the activating Ser1177 site of eNOS. Both modifications blunt eNOS activity and reduce NO availability, thereby inhibiting vasodilation. These mechanistic insights were further validated functionally, as pharmacologic inhibition of mtROS or PKCβ, and using a CPS-deficient mutant, partially restored vasodilation, and their combination fully restored vascular response. The study reveals a previously unappreciated role of T6SS and CPS in manipulating host vascular physiology through post-translational control of eNOS. By addressing a poorly explored niche in host-pathogen interaction, the authors fill an essential gap in our understanding of bloodstream infections. Moreover, they propose a novel role for NLRX1 and mtROS signaling in vascular

dysfunction, opening doors for future investigation into host-directed therapies.
2. Evaluation of Experimental Evidence
The authors present a well-structured, multi-layered experimental framework. From molecular dissection in human endothelial cells to functional validation in ex vivo blood vessels, the study convincingly demonstrates how K. pneumoniae impairs vasodilation. The evidence for both VgrG4- and CPS-dependent modulation of eNOS is comprehensive and methodologically sound. Importantly, pharmacologic and genetic tools converge to support the authors' claims. However, certain mechanistic connections, particularly between the two pathways—are treated independently without defining whether they act redundantly, synergistically, or temporally apart. The study's conclusions are strong overall, but a few clarifications would enhance interpretability and ensure completeness.
3. General Critique and Conditional Recommendation
This compelling and timely study combines elegant mechanistic insight with a novel physiological model. The experiments are exceptionally well designed, and ex vivo blood vessels add translational value. However, several points merit refinement. The manuscript structure occasionally feels fragmented, with abrupt shifts between the T6SS and CPS narratives. While the functional data indicates additive roles, the relationship between the two pathways remains undefined, and a more explicit discussion of this point would help unify the findings. Similarly, the VgrG4 pathway appears sufficient to block eNOS activity; the rationale for invoking the CPS–EGFR–PP2Ac axis could be more clearly justified beyond redundancy. Although the use of mutants and inhibitors is strong, the absence of genetic complementation for ΔvgrG4, ΔtssB, and ΔmanC somewhat limits the conclusiveness of these loss-of-function results. A $CPS^{-}/T6SS^{-}$ double mutant would have further solidified the independence or interaction of the two mechanisms. While technically reasonable, using HULEC-5a cells stimulated with histamine deviates from the ACh–M3–eNOS axis and may not reflect vascular physiology as directly as the ex vivo system. Lastly, while the inhibition of EDH is functionally supported, the underlying molecular mechanism remains speculative.
That said, the novelty and depth of this study are undeniable. The authors propose an innovative mechanism whereby K. pneumoniae licenses host homeostatic enzymes (PKCβ and PP2Ac) to suppress eNOS activation. This strategy represents a paradigm shift from direct effector-target interactions to subtle manipulation of host regulatory circuits. Furthermore, suggesting that K. pneumoniae-induced vascular dysfunction resembles cardiovascular disease adds translational appeal.

4. Reviewer Questions for Clarification
1. Are the T6SS and CPS pathways functionally redundant, complementary, or temporally distinct?
Clarifying their relationship would greatly aid in interpreting the biological significance of the dual mechanisms.
2. Why was a $CPS^{-}/T6SS^{-}$ double mutant not included?
Although pharmacologic rescue and mutant-inhibitor combinations support their additive effects, a genetic double mutant would offer more conclusive validation.
3. Why was HULEC-5a used for histamine-induced Ca^{2+} imaging rather than a model that retains ACh responsiveness?
Could Ca^{2+} signaling have been assessed in the ex vivo model for physiological alignment?
4. How general is this vasodilation inhibition strategy?
Has the same phenotype been tested with other bloodstream pathogens such as E. coli, Pseudomonas, or Vibrio vulnificus?
5. Can the vascular dysfunction observed here directly link to host mortality or impaired immune cell trafficking in vivo?
This would substantiate the translational relevance of the findings.

5. Suggested Additional Experiments (if feasible)
• Genetic complementation of key mutants
Reintroducing vgrG4, tssB, and manC under native promoters would strengthen the genetic conclusions.
• Construction and analysis of a $CPS^{-}/T6SS^{-}$ double mutant
This would directly test whether both pathways are necessary for full eNOS inhibition.
• ACh-induced Ca^{2+} signaling assessment in ex vivo vessels
This would offer a more direct link between intracellular signaling and physiological outcomes.
• Broader pathogen comparison
Including other sepsis-causing bacteria would help determine the uniqueness of K. pneumoniae's strategy.
• Mechanistic dissection of EDH suppression by mtROS
Investigating ion channel function or gap junction integrity would clarify how mtROS blunts hyperpolarization.

Decision Letter:

2nd April 2025

*Please ensure you delete the link to your author homepage in this e-mail if you wish to forward it to your co-authors.

Dear Jose,

Thank you very much for your patience while your manuscript "K. pneumoniae disrupts vasodilation by targeting eNOS post translational modifications via the type VI secretion system and the capsule polysaccharide" was under peer-review at Nature Microbiology. It has now been seen by 4 referees, whose expertise and comments you will find at the end of this email. Although they find your work of some potential interest, they have raised a number of concerns that will need to be addressed before we can consider publication of the work in Nature Microbiology.

In particular, several of the referees raised questions over the relevance of this phenotype for in vivo infection, listing possible experiments that should help address this question. In addition, Referee #4 raised questions over whether the Vgr4 and capsule-induced pathways are independent and/or redundant, and highlighted that experiments with a double mutant strain would help address this point. They also pointed out that complementation of mutant strains was also needed to confirm the involvement of

the virulence factors in the phenotype described. Referees #3 and #4 also mentioned that questions remained over the mechanism between ROS generation and EDH, suggesting some experiments that should address this point. Other important referee points included confirmation of a lack of vascular damage by alternative methods, and orthologous validation of some of the Western blot data. Further details are provided in the referee reports below. We feel that these are critical points which should be addressed in a revised manuscript.

There were additional points raised by the referees, including determining which vascular sites in vivo were targeted by this behaviour, to explore further the therapeutic potential of coupling host-directed therapy and antibiotic treatments in control of Klebsiella infection, and to test whether other bacterial pathogens also exhibit similar phenotypes. While we recognise that all of these points would expand and develop the existing manuscript, it's not essential that they be addressed for us to consider the revised paper, particularly as some may be out of scope for this study.

Should further experimental data allow you to address these criticisms, we would be happy to look at a revised manuscript.

We are committed to providing a fair and constructive peer-review process. Please do not hesitate to contact us if there are specific requests from the reviewers that you believe are technically impossible or unlikely to yield a meaningful outcome.

We strongly support public availability of data. Please place the data used in your paper into a public data repository, if one exists, or alternatively, present the data as Source Data or Supplementary Information. If data can only be shared on request, please explain why in your Data Availability Statement, and also in the correspondence with your editor. For some data types, deposition in a public repository is mandatory - more information on our data deposition policies and available repositories can be found at https://www.nature.com/nature-research/editorial-policies/reporting-standards#availability-of-data.

Please include a data availability statement as a separate section after Methods but before references, under the heading "Data Availability". This section should inform readers about the availability of the data used to support the conclusions of your study. This information includes accession codes to public repositories (data banks for protein, DNA or RNA sequences, microarray, proteomics data etc…), references to source data published alongside the paper, unique identifiers such as URLs to data repository entries, or data set DOIs, and any other statement about data availability. At a minimum, you should include the following statement: "The data that support the findings of this study are available from the corresponding author upon request", mentioning any restrictions on availability. If DOIs are provided, we also strongly encourage including these in the Reference list (authors, title, publisher (repository name), identifier, year). For more guidance on how to write this section please see: http://www.nature.com/authors/policies/data/data-availability-statements-data-citations.pdf

If revising your manuscript:

* Include a "Response to referees" document detailing, point-by-point, how you addressed each referee comment. If no action was taken to address a point, you must provide a compelling argument. This response will be sent back to the referees along with the revised manuscript.

* If you have not done so already we suggest that you begin to revise your manuscript so that it conforms to our Article format instructions at http://www.nature.com/nmicrobiol/info/final-submission. Refer also to any guidelines provided in this letter.

* Include a revised version of any required reporting checklist. It will be available to referees (and, potentially, statisticians) to aid in their evaluation if the manuscript goes back for peer review. A revised checklist is essential for re-review of the paper.

When submitting the revised version of your manuscript, please pay close attention to our href="https://www.nature.com/nature-portfolio/editorial-policies/image-integrity">Digital Image Integrity Guidelines. and to the following points below:

-- that unprocessed scans are clearly labelled and match the gels and western blots presented in figures.
-- that control panels for gels and western blots are appropriately described as loading on sample processing controls
-- all images in the paper are checked for duplication of panels and for splicing of gel lanes.

EXTENDED DATA FIGURES

When re-submitting your manuscript, please ensure that any supplementary figures and tables that are crucial to the manuscript's conclusions are converted into Extended Data figures and tables to increase visibility of these data. Extended Data figures and tables are online-only (present in the online PDF and full-text HTML versions of the paper), peer-reviewed display items that provide essential background to the article but are not included in the main article due to space constraints. A maximum of ten Extended Data display items (figures and tables) is permitted.

Finally, please ensure that you retain unprocessed data and metadata files after publication, ideally archiving data in perpetuity, as these may be requested during the peer review and production process or after publication if any issues arise.

Please use the link below to submit a revised paper:

Link Redacted

Note: This url links to your confidential homepage and associated information about manuscripts you may have submitted or be

reviewing for us. If you wish to forward this e-mail to co-authors, please delete this link to your homepage first.

Nature Microbiology is committed to improving transparency in authorship. As part of our efforts in this direction, we are now requesting that all authors identified as ‘corresponding author’ on published papers create and link their Open Researcher and Contributor Identifier (ORCID) with their account on the Manuscript Tracking System (MTS), prior to acceptance. This applies to primary research papers only. ORCID helps the scientific community achieve unambiguous attribution of all scholarly contributions. You can create and link your ORCID from the home page of the MTS by clicking on ‘Modify my Springer Nature account’. For more information please visit please visit www.springernature.com/orcid.

If you wish to submit a suitably revised manuscript we would hope to receive it within 6 months. If you cannot send it within this time, please let us know. We will be happy to consider your revision, even if a similar study has been accepted for publication at Nature Microbiology or published elsewhere (up to a maximum of 6 months).

In the meantime we hope that you find our referees' comments helpful.

****
Reviewer Expertise:

Referee #1: Vascular biology
Referee #2: Klebsiella pathogenesis
Referee #3: vascular biology
Referee #4: T6SS biology

Reviewer Comments:

Reviewer #1 (Remarks to the Author):

This manuscript addresses a largely understudied aspect of Klebsiella pneumoniae pathogenesis: the bacterium’s ability to disrupt vascular function by targeting the endothelial nitric oxide synthase (eNOS), thereby inhibiting agonist-induced vasodilation. While endothelial dysfunction has been extensively examined in the context of viral haemorrhagic infections (e.g., Ebola) and in bacterial sepsis more generally, few studies have dissected K. pneumoniae’s direct mechanisms of impairing vascular function.

Overall, I think the study is robust and well-presented and does a good job at analysing the mechanistic aspects of the biology. I feel a few presentation elements might improve reader experience:

Enhanced Timeline Schematics: A diagram illustrating the timeline of infection, addition of agonists, and sample collection in each experiment would help readers better follow the experimental steps.

Statistics and Replicates: Although the paper states the number of biological replicates in multiple sections, consistently emphasising technical replicates (e.g., n=3, each in triplicate) vs. biological replicates (e.g., 3 separate rats per group) would clarify the robustness of some of the data.

The discussion highlights many possible research expansions, but a concise “Future Directions” section could strengthen the discussion.

In addition, it’s worth considering a few additional studies to strengthen the potential translational impact:

The ex vivo model is a powerful tool, but the study stops short of verifying this vascular dysfunction in vivo or in other vascular beds (e.g., pulmonary, renal, or cerebral microcirculation):

Testing in an animal infection model (e.g., mice or rats) to measure blood pressure, tissue perfusion, or immune-cell infiltration would confirm physiological relevance. This could be done, perhaps, by comparing wild-type K. pneumoniae versus T6SS/capsule mutants.

It might be insightful to examine if the same T6SS-dependent phenomenon occurs in coronary, renal, or cerebral arteries, especially since (I believe) K. pneumoniae commonly causes disseminated infections.

The study suggests potential avenues for host-targeted therapies, such as PKCβ inhibition or mtROS scavenging, to restore vasodilation:

Evaluating whether in vitro or ex vivo treatments combining bactericidal agents (e.g., colistin or carbapenems) plus a PKCβ inhibitor or MitoTEMPO enhances bacterial clearance or reduces vascular damage might appeal to clinicians.

A fuller characterisation of inhibitor efficacy, toxicity ranges, and potential off-target effects in a more physiologically relevant model could help transition these findings into animal models or early translational studies.

Reviewer #2 (Remarks to the Author):

In this study by Rehman, et al. the authors seek to define mechanisms by which Klebsiella pneumoniae influences vasodilation. Using a creative ex vivo approach utilizing rat tissue, the authors constrict blood vessels with the chemical phenylephrine (PE) and monitor vessel dilation with acetylcholine (ACh). In this system, they observe that K. pneumoniae inhibits blood vessel re-dilation after the ACh treatment. To further probe this finding, the authors use in vitro infection of human endothelial cells to characterize post-translational modifications within the eNOS pathway that correlate with inhibition of re-dilation. This study addresses vascular biology, an important aspect of K. pneumoniae infection that has not been investigated in detail in previous literature. While the authors should be commended for a mechanistic study about an understudied aspect of K. pneumoniae infection, I am concerned about the relevance of these findings to infection biology in an in vivo context, and I was not fully convinced of the findings surrounding capsule polysaccharide (CPS) limiting phosphorylation of Ser177 in eNOS.
Major Concerns:
1. The authors present an innovative method to monitor blood vessel constriction and dilation in an ex vivo manner. While this should be commended as it avoids the excess use of animals, I am not convinced that this model accurately reflects bacteremia pathology. First, systemic infections include the presence of cytokines, circulating immune cells, and serum which are all important for host-pathogen interactions in bacteremia and work in a coordinated manner. Without these factors present in the ex vivo model, it is difficult to understand whether the observations observed in this study are also present in vivo. Second, during Gram-negative bacteremia, bacteria are rarely found in the bloodstream itself and are quickly sequestered to organs like the liver. Third, the authors should contextualize their findings considering other K. pneumoniae bacteremia pathogenesis work. In a recent study (PMID37463183), the authors used transposon sequencing in ATCC43816 (the same strain used in the present study) to identify K. pneumoniae factors important for survival during in vivo bloodstream infection. In that study, all T6SS genes were dispensable for infection. Thus, although the data in Figure 2 is compelling that the T6SS influences vasodilation ex vivo, due to this pre-existing study, I am not sure that the T6SS specifically impacts K. pneumoniae pathogenicity in the bloodstream. To address this, the author could consider competing their T6SS mutant against WT K. pneumoniae in an intravascular model of bacteremia to demonstrate that the T6SS mutant is less fit during infection.
2. The conclusion from Figure 4 is unclear. The authors suggest that Kp43816 inhibits Ser177 phosphorylation that is induced by eNOS agonists, but this is not apparent by eye. From the raw blot in 4B, it appears that the entire gel lane for the uninfected wells may be darker than the lanes in the Kp43816 lanes, which would make the densitometry calculation in 4B appear higher in the uninfected wells (since the numerator would be a higher value due to a darker lane). These results seem very subtle, and by eye they are undetectable. I am not convinced that this conclusion is supported by the data without a secondary means of confirmation such as knockout cell lines or a reporter assay.
3. Why did the authors choose the timepoints that are used in this study? The results in this study are shown over several hours, but did the authors consider a more prolonged timepoint to see if protein modifications are more dramatically altered later during infection? For example, can the T6SS initiate signaling within minutes? Is it possible that T6SS mutants would be able to influence post translational modifications at longer timepoints, like 24 hours?
Minor Concerns:
1. In Line 154, please update the phrasing to say that “inhibition of validation is observed across multiple diverse strains of K. pneumoniae.” The authors could also consider noting that the different strains of K. pneumoniae produce very different effects in this figure. Thus, some K. pneumoniae strains may inhibit dilation to a higher degree than others, which is an interesting finding.
2. The results of Panel 3D are statistically significant but very subtle. Can the authors consider adding lines for pairwise connection of trials to demonstrate a consistent increase in Ca2+ response during each repeat?

Reviewer #3 (Remarks to the Author):

This manuscript describes the effects of infection of endothelial cells with K. pneumoniae on endothelium dependent vasodilation and on signal transduction pathways that appear to mediate inhibition of endothelium-dependent vasodilation using cultured human endothelial cells. Overall this is a well conducted study that clearly outlines the effects of K. pneumoniae infection on endothelium dependent vasodilation and identification of the signaling pathways involved in this inhibition. I only have two significant concerns. The first relates the the authors statement that K. pneumoniae infection has no effect on endothelium apparently based solely on histological examination of vessel longitudinal sections. It is not clear to this reviewer that it is possible to assess, for example, the Barries function of the endothelium using this simple approach. It is suggested that a more rigorous approach be utilized to assessed endothelial structural integrity, or that this statement be softened. Second, while the authors clearly show that K. pneumoniae infection blunts EDH mediated dilation and that Mito-ROS is involved, they have not shown how this blunting is accomplished. Does K. pneumoniae infection reduce dilation mediated by NS-319 or other IKCa/SKCa agonist or IKCa and/or SKCa currents in isolated endothelia cells? Such findings would better "close the loop" for this aspect of the authors findings.

Reviewer #4 (Remarks to the Author):

1. Summary of the Study
In this study, the authors establish a robust ex-vivo vascular model to investigate how Klebsiella pneumoniae infection affects vasodilation. Using rat mesenteric arteries and human endothelial cells, they show that K. pneumoniae impairs acetylcholine-induced vasodilation by targeting nitric oxide (NO) and endothelium-derived hyperpolarization (EDH) pathways. Mechanistically, they identify two parallel bacterial strategies: (1) the trans-kingdom T6SS effector VgrG4 induces mitochondrial ROS (mtROS) in an NLRX1-dependent manner, activating PKCβ and phosphorylating the inhibitory Thr495 site of eNOS, and (2) the capsular polysaccharide (CPS) activates an EGFR-dependent signaling cascade, leading to PP2Ac upregulation and dephosphorylation of the activating Ser1177 site of eNOS. Both modifications blunt eNOS activity and reduce NO availability, thereby inhibiting

vasodilation. These mechanistic insights were further validated functionally, as pharmacologic inhibition of mtROS or PKCβ, and using a CPS-deficient mutant, partially restored vasodilation, and their combination fully restored vascular response. The study reveals a previously unappreciated role of T6SS and CPS in manipulating host vascular physiology through post-translational control of eNOS. By addressing a poorly explored niche in host-pathogen interaction, the authors fill an essential gap in our understanding of bloodstream infections. Moreover, they propose a novel role for NLRX1 and mtROS signaling in vascular dysfunction, opening doors for future investigation into host-directed therapies.
2. Evaluation of Experimental Evidence
The authors present a well-structured, multi-layered experimental framework. From molecular dissection in human endothelial cells to functional validation in ex vivo blood vessels, the study convincingly demonstrates how K. pneumoniae impairs vasodilation. The evidence for both VgrG4- and CPS-dependent modulation of eNOS is comprehensive and methodologically sound. Importantly, pharmacologic and genetic tools converge to support the authors' claims. However, certain mechanistic connections, particularly between the two pathways—are treated independently without defining whether they act redundantly, synergistically, or temporally apart. The study's conclusions are strong overall, but a few clarifications would enhance interpretability and ensure completeness.
3. General Critique and Conditional Recommendation
This compelling and timely study combines elegant mechanistic insight with a novel physiological model. The experiments are exceptionally well designed, and ex vivo blood vessels add translational value. However, several points merit refinement. The manuscript structure occasionally feels fragmented, with abrupt shifts between the T6SS and CPS narratives. While the functional data indicates additive roles, the relationship between the two pathways remains undefined, and a more explicit discussion of this point would help unify the findings. Similarly, the VgrG4 pathway appears sufficient to block eNOS activity; the rationale for invoking the CPS–EGFR–PP2Ac axis could be more clearly justified beyond redundancy. Although the use of mutants and inhibitors is strong, the absence of genetic complementation for ΔvgrG4, ΔtssB, and ΔmanC somewhat limits the conclusiveness of these loss-of-function results. A CPS^-/$T6SS^-$ double mutant would have further solidified the independence or interaction of the two mechanisms. While technically reasonable, using HULEC-5a cells stimulated with histamine deviates from the ACh–M3–eNOS axis and may not reflect vascular physiology as directly as the ex vivo system. Lastly, while the inhibition of EDH is functionally supported, the underlying molecular mechanism remains speculative.
That said, the novelty and depth of this study are undeniable. The authors propose an innovative mechanism whereby K. pneumoniae licenses host homeostatic enzymes (PKCβ and PP2Ac) to suppress eNOS activation. This strategy represents a paradigm shift from direct effector-target interactions to subtle manipulation of host regulatory circuits. Furthermore, suggesting that K. pneumoniae-induced vascular dysfunction resembles cardiovascular disease adds translational appeal.

4. Reviewer Questions for Clarification
1. Are the T6SS and CPS pathways functionally redundant, complementary, or temporally distinct?
Clarifying their relationship would greatly aid in interpreting the biological significance of the dual mechanisms.
2. Why was a CPS^-/$T6SS^-$ double mutant not included?
Although pharmacologic rescue and mutant-inhibitor combinations support their additive effects, a genetic double mutant would offer more conclusive validation.
3. Why was HULEC-5a used for histamine-induced Ca^{2+} imaging rather than a model that retains ACh responsiveness?
Could Ca^{2+} signaling have been assessed in the ex vivo model for physiological alignment?
4. How general is this vasodilation inhibition strategy?
Has the same phenotype been tested with other bloodstream pathogens such as E. coli, Pseudomonas, or Vibrio vulnificus?
5. Can the vascular dysfunction observed here directly link to host mortality or impaired immune cell trafficking in vivo?
This would substantiate the translational relevance of the findings.

5. Suggested Additional Experiments (if feasible)
• Genetic complementation of key mutants
Reintroducing vgrG4, tssB, and manC under native promoters would strengthen the genetic conclusions.
• Construction and analysis of a CPS^-/$T6SS^-$ double mutant
This would directly test whether both pathways are necessary for full eNOS inhibition.
• ACh-induced Ca^{2+} signaling assessment in ex vivo vessels
This would offer a more direct link between intracellular signaling and physiological outcomes.
• Broader pathogen comparison
Including other sepsis-causing bacteria would help determine the uniqueness of K. pneumoniae's strategy.
• Mechanistic dissection of EDH suppression by mtROS
Investigating ion channel function or gap junction integrity would clarify how mtROS blunts hyperpolarization.

****

Version 1:

Reviewer comments:

Reviewer #1

(Remarks to the Author)
The authors have been highly responsive to the reviewer comments and have substantially strengthened the manuscript. In

particular, the addition of in vivo blood pressure measurements, the inclusion of relevant mutant analyses, improved figure schematics, clearer reporting of biological versus technical replicates, and expanded mechanistic experiments addressing EDH signaling significantly enhance the physiological relevance, clarity, and impact of the study.

The revised discussion appropriately contextualises the findings, clearly delineates scope, and outlines future directions without overextending the conclusions. Concerns regarding translational relevance and mechanistic completeness have been addressed to a level that is appropriate for the present study.

Overall, I am satisfied with the revisions and believe the manuscript is now robust, well-presented, and suitable for publication.

Reviewer #2

(Remarks to the Author)
This review is in response to a revised manuscript submitted by Rehman and colleagues. In this revised manuscript, the authors incorporate new in vivo data, ex vivo data, and text modifications to strengthen their original manuscript. While the authors have addressed many critiques raised by the reviewers, some of my major comments remain unsatisfied from the original peer review. Additionally, I have a number of new points of concern which were missed upon the initial review which I believe are important considerations for the ultimate publication of the manuscript. My comments are outlined below into three sections: 1) comments regarding the revision, 2) new concerns, and 3) comments on statistical analysis.
Comments Regarding the Revision:
1. In the Response to Reviewers document, my original concern was that Gram-negative species like Klebsiella pneumoniae are difficult to recover from the blood – in both mouse tail vein injection models and in clinical settings. The authors rebut saying that Klebsiella is clearly found in the blood by clinical culture systems, but this is not justified with a citation. It is true that blood culture methods are a gold standard for clinical diagnosis of bacteremia but this rebuttal comment requires a citation stating how readily abundant K. pneumoniae is in the bloodstream of affected patients so that the present study can be properly contextualized. Some bloodstream infections require multiple cultures of blood to obtain a single CFU of bacteria for diagnosis. If K. pneumoniae is one such species, then vasodilation may not be a clinically translatable phenomenon – and vice versa.
2. In the Response to Reviewers document, the authors discuss findings from a previous study performed by the Bachman lab that identified K. pneumoniae factors contributing to bacteremia. The authors state that "no hits were found in the capsule polysaccharide" but this is not entirely true. Capsule polysaccharide genes were not present in the input of the Bachman study, so they are missing from the identified genes because they are not included in the study. More importantly for the present work, the Bachman lab study found no connection between T6SS and K. pneumoniae bacteremia pathogenesis. Since that supports the author's modified data, this should be discussed within the paper or at the very least cited. It's omission from the discussion is disappointing as this study helps to contextualize the present study within the pathogenesis field.
New Concerns:
1. T6SS is not activated in K. pneumoniae grown under standard lab conditions stated in the Materials and Methods, and can take a while to become activated under environmental pressure (PMID39843522). Given the short time period of the experiments in this present study, the authors should demonstrate that the T6SS is activated at the start of these experiments or during the experimental conditions. Otherwise, the effects due to vgrG4 mutations could be due to genetic functions unrelated to the T6SS. Additionally, the K. pneumoniae T6SS contains two loci. In the absence of the vgrG4 system, the other loci may still be active. If T6SS machinery is required but the system is off, then these genes would have novel unannotated function in vasodilation which would help contextualize these findings. The authors should discuss this in the manuscript and, at the very least, cite other T6SS papers for K. pneumoniae which investigate this virulence factor (PMID39843522).
2. In the paragraph with Text lines 170-175, the authors should call out where in the text their statements are supported. For example, which experiments conclude that K. pneumoniae blunts the NO pathway (Line 174)? And where are the in vivo results in question (Line 175)? These should be stated in the text to improve readability.
3. It is unclear why some dilation data is presented as the representative Outer Diameter Reads (Figure 1C-F) while others are presented as a Dilation curve (Figure 1H). This occurs in the supplemental data as well. The Outer Diameter Reads do not allow for statistical comparison or for showing multiple replicates and are difficult to read. The Dilation curves are much more intuitive.
4. Figure 2A should be updated to resolve differences between the delta-tssB and delta-vgrG3com strain. As is, I cannot tell which lines corresponds to which strain in the present panel.
5. In line 257-259, the authors state that "in the cells infected with either the tssB mutant or YeVgrG4 we did not observe any decrease in the agonist-induced Ser1177 phosphorylation". This does not seem to be the case by eye. In fact, the tssB mutant appears to have no Ser1177 phosphorylation. This confusion is furthered by the lack of densitometry for Figure 4C. Can the authors please restate this sentence to increase the clarity of this section.
6. In the Discussion lines 441-443, the authors state that inhibition of vasodilation impairs the recruitment of immune cells into infected tissue. If the authors believe this to be the case for K. pneumoniae, then how do these findings fit within the field's previous findings that Klebsiella infections are highly inflammatory and often lead to sepsis? It is difficult to connect these two points. This goes to a larger concern that the data is not fully contextualized within the Klebsiella pathogenesis field (see prior comments about no citations for other Klebsiella bloodstream infection studies or T6SS studies).
Comments on Statistical Analysis:
Throughout the manuscript, statistics are not adequately applied. Error bars are not always defined in the figure legends (Figure 1I, Figure 2B – there are no error bars on some of the points, Figure 4 densitometry, Figure 5 densitometry, Figure 6 densitometry, Figure 7B, Figure 7D-F). In many cases, there is also a lack of description on whether dots represent technical or biological/independent trials. The biggest concern on statistics surrounds the comparisons that were made, which is detailed in Point 1 below.
1. In Figure 4, statistics should be applied for the densitometry comparing the control to the Kp strain in question: for example, bradykinin control vs. bradykinin Kp43816. As is, the statistics are just demonstrating that the agonists worked and not that Kp is inducing a meaningful difference. This comment also extend to the densitometry in subsequent figures.
2. Figure 1G, the authors state in the figure caption that pathology is being scored. What are the pathology markers being observed? Is this damage, death, infiltration?

3. In the legend for Figure 3, the differences between panels Bi and Bii should be detailed for the reader. It is unclear how the traces in Bi differ from those represented in 3C.
4. In Figure 3D, the authors state in the caption that a two-way ANOVA is performed, but this would not be the case for such a data set. Should this be a paired t-test?
5. In Figure 3E-F, statistics are lacking. It is unclear if these results would be significant or if they were measured and are not significant. If measured, the authors should either indicate ns above the comparison or state that it was measured but it not displayed in the legend.
6. Figure 4C is missing densitometry but it is included in the figure caption.
7. In figure 5B, the densitometry plots only show two replicates.
8. In the new mouse data in Figure 1 and Figure 7, please indicate whether two independent trials were performed for each experiment. A single infection is not generally considered a robust trial as inoculum and animal cage variables can influence day-to-day factors such as stress and blood pressure.

Reviewer #3

(Remarks to the Author)
The authors have adequately addressed my concerns, although I will comment that the use of historical data to support the idea that the vessels are still able to dilate is less than optimal from a rigor and reproducibility standpoint. I have no further comments.

Reviewer #4

(Remarks to the Author)
The authors have substantially strengthened the manuscript and should be commended for the rigorous experimental work completed during revision. The addition of the CPS/vgrG4 double mutant and its phenotypic characterization clearly defines the additive and concurrent roles of the T6SS/VgrG4 and CPS pathways, resolving a major mechanistic gap in the original submission. The new in vivo blood pressure data greatly enhance the translational relevance of the study, and the expanded analysis of EDH impairment, including the NS309 experiments, provides a more complete mechanistic understanding of mtROS-dependent endothelial dysfunction.

Decision Letter:

7th January 2026

*Please ensure you delete the link to your author homepage in this e-mail if you wish to forward it to your co-authors.

Dear Professor Bengoechea,

First of all, happy New Year! I hope it has been a good start to 2026, and thanks a lot for your patience while we waited for the last few reports from the reviewers for the review for your revised manuscript. The manuscript, "K. pneumoniae disrupts vasodilation by targeting eNOS post translational modifications via the type VI secretion system and the capsule polysaccharide" has now been seen by the original referees, whose comments you will find at the of this email. You will see from their comments below that they find your work has improved through revision, however there are still a few points remaining to address. We are very interested in the possibility of publishing your study in Nature Microbiology, but would like to consider your response to these concerns in the form of a revised manuscript before we make a final decision on publication.

It is important that the concerns raised by the reviewers are addressed with data, or text and figure edits, in the manuscript and rebuttal as appropriate. Addressing concerns regarding the statistics and replicates is essential. There is also a comment regarding the need to demonstrate T6SS activity under the experimental conditions used, which should be addressed either with new data, or with existing data that demonstrates T6SS activity in this set-up. The rest referees' reports are clear and the remaining issues should be straightforward to address.

We are committed to providing a fair and constructive peer-review process. Do not hesitate to contact us if there are specific requests from the reviewers that you believe are technically impossible or unlikely to yield a meaningful outcome.

If you have not done so already please begin to revise your manuscript so that it conforms to our Article format instructions at http://www.nature.com/nmicrobiol/info/final-submission/

The usual length limit for a Nature Microbiology Article is six display items (figures or tables) and 3,000 words. We have some flexibility, and can allow a revised manuscript at 3,500 words, but please consider this a firm upper limit. There is a trade-off of ~250 words per display item, so if you need more space, you could move a Figure or Table to Supplementary Information.

Some reduction could be achieved by focusing any introductory material and moving it to the start of your opening 'bold' paragraph, whose function is to outline the background to your work, describe in a sentence your new observations, and explain your main conclusions. The discussion should also be limited. Methods should be described in a separate section following the discussion, we do not place a word limit on Methods.

Nature Microbiology titles should give a sense of the main new findings of a manuscript, and should not contain punctuation. Please keep in mind that we strongly discourage active verbs in titles, and that they should ideally fit within 90 characters each (including spaces).

We strongly support public availability of data. Please place the data used in your paper into a public data repository, if one

exists, or alternatively, present the data as Source Data or Supplementary Information. If data can only be shared on request, please explain why in your Data Availability Statement, and also in the correspondence with your editor. For some data types, deposition in a public repository is mandatory - more information on our data deposition policies and available repositories can be found at https://www.nature.com/nature-research/editorial-policies/reporting-standards#availability-of-data.

Please include a data availability statement as a separate section after Methods but before references, under the heading "Data Availability". This section should inform readers about the availability of the data used to support the conclusions of your study. This information includes accession codes to public repositories (data banks for protein, DNA or RNA sequences, microarray, proteomics data etc…), references to source data published alongside the paper, unique identifiers such as URLs to data repository entries, or data set DOIs, and any other statement about data availability. At a minimum, you should include the following statement: "The data that support the findings of this study are available from the corresponding author upon request", mentioning any restrictions on availability. If DOIs are provided, we also strongly encourage including these in the Reference list (authors, title, publisher (repository name), identifier, year). For more guidance on how to write this section please see: http://www.nature.com/authors/policies/data/data-availability-statements-data-citations.pdf

To improve the accessibility of your paper to readers from other research areas, please pay particular attention to the wording of the paper's opening bold paragraph, which serves both as an introduction and as a brief, non-technical summary in about 150 words. If, however, you require one or two extra sentences to explain your work clearly, please include them even if the paragraph is over-length as a result. The opening paragraph should not contain references. Because scientists from other sub-disciplines will be interested in your results and their implications, it is important to explain essential but specialised terms concisely. We suggest you show your summary paragraph to colleagues in other fields to uncover any problematic concepts.

If your paper is accepted for publication, we will edit your display items electronically so they conform to our house style and will reproduce clearly in print. If necessary, we will re-size figures to fit single or double column width. If your figures contain several parts, the parts should form a neat rectangle when assembled. Choosing the right electronic format at this stage will speed up the processing of your paper and give the best possible results in print. We would like the figures to be supplied as vector files - EPS, PDF, AI or postscript (PS) file formats (not raster or bitmap files), preferably generated with vector-graphics software (Adobe Illustrator for example). Please try to ensure that all figures are non-flattened and fully editable. All images should be at least 300 dpi resolution (when figures are scaled to approximately the size that they are to be printed at) and in RGB colour format. Please do not submit Jpeg or flattened TIFF files. Please see also 'Guidelines for Electronic Submission of Figures' at the end of this letter for further detail.

Figure legends must provide a brief description of the figure and the symbols used, within 350 words, including definitions of any error bars employed in the figures.

When submitting the revised version of your manuscript, please pay close attention to our href="https://www.nature.com/nature-research/editorial-policies/image-integrity">Digital Image Integrity Guidelines. and to the following points below:

-- that unprocessed scans are clearly labelled and match the gels and western blots presented in figures.
-- that control panels for gels and western blots are appropriately described as loading on sample processing controls
-- all images in the paper are checked for duplication of panels and for splicing of gel lanes.

EXTENDED DATA FIGURES

When re-submitting your manuscript, please ensure that any supplementary figures and tables that are crucial to the manuscript's conclusions are converted into Extended Data figures and tables to increase visibility of these data. Extended Data figures and tables are online-only (present in the online PDF and full-text HTML versions of the paper), peer-reviewed display items that provide essential background to the article but are not included in the main article due to space constraints. A maximum of ten Extended Data display items (figures and tables) is permitted.

Finally, please ensure that you retain unprocessed data and metadata files after publication, ideally archiving data in perpetuity, as these may be requested during the peer review and production process or after publication if any issues arise.

Please include a statement before the acknowledgements naming the author to whom correspondence and requests for materials should be addressed.

Finally, we require authors to include a statement of their individual contributions to the paper -- such as experimental work, project planning, data analysis, etc. -- immediately after the acknowledgements. The statement should be short, and refer to authors by their initials. For details please see the Authorship section of our joint Editorial policies at http://www.nature.com/authors/editorial_policies/authorship.html

When revising your paper:

* include a point-by-point response to any editorial suggestions and to our referees. Please include your response to the editorial suggestions in your cover letter, and please upload your response to the referees as a separate document.

* ensure it complies with our format requirements for Letters as set out in our guide to authors at www.nature.com/nmicrobiol/info/gta/

* state in a cover note the length of the text, methods and legends; the number of references; number and estimated final size of figures and tables

* resubmit electronically if possible using the link below to access your home page:

Link Redacted

*This url links to your confidential homepage and associated information about manuscripts you may have submitted or be reviewing for us. If you wish to forward this e-mail to co-authors, please delete this link to your homepage first.

Please ensure that all correspondence is marked with your Nature Microbiology reference number in the subject line.

Nature Microbiology is committed to improving transparency in authorship. As part of our efforts in this direction, we are now requesting that all authors identified as 'corresponding author' on published papers create and link their Open Researcher and Contributor Identifier (ORCID) with their account on the Manuscript Tracking System (MTS), prior to acceptance. This applies to primary research papers only. ORCID helps the scientific community achieve unambiguous attribution of all scholarly contributions. You can create and link your ORCID from the home page of the MTS by clicking on 'Modify my Springer Nature account'. For more information please visit please visit www.springernature.com/orcid.

We hope to receive your revised paper within three weeks. If you cannot send it within this time, please let us know.

We look forward to hearing from you soon.

Yours sincerely,

Nature Microbiology

****

Reviewers Comments:

Reviewer #1 (Remarks to the Author):

The authors have been highly responsive to the reviewer comments and have substantially strengthened the manuscript. In particular, the addition of in vivo blood pressure measurements, the inclusion of relevant mutant analyses, improved figure schematics, clearer reporting of biological versus technical replicates, and expanded mechanistic experiments addressing EDH signaling significantly enhance the physiological relevance, clarity, and impact of the study.

The revised discussion appropriately contextualises the findings, clearly delineates scope, and outlines future directions without overextending the conclusions. Concerns regarding translational relevance and mechanistic completeness have been addressed to a level that is appropriate for the present study.

Overall, I am satisfied with the revisions and believe the manuscript is now robust, well-presented, and suitable for publication.

Reviewer #2 (Remarks to the Author):

This review is in response to a revised manuscript submitted by Rehman and colleagues. In this revised manuscript, the authors incorporate new in vivo data, ex vivo data, and text modifications to strengthen their original manuscript. While the authors have addressed many critiques raised by the reviewers, some of my major comments remain unsatisfied from the original peer review. Additionally, I have a number of new points of concern which were missed upon the initial review which I believe are important considerations for the ultimate publication of the manuscript. My comments are outlined below into three sections: 1) comments regarding the revision, 2) new concerns, and 3) comments on statistical analysis.
Comments Regarding the Revision:
1. In the Response to Reviewers document, my original concern was that Gram-negative species like Klebsiella pneumoniae are difficult to recover from the blood – in both mouse tail vein injection models and in clinical settings. The authors rebut saying that Klebsiella is clearly found in the blood by clinical culture systems, but this is not justified with a citation. It is true that blood culture methods are a gold standard for clinical diagnosis of bacteremia but this rebuttal comment requires a citation stating how readily abundant K. pneumoniae is in the bloodstream of affected patients so that the present study can be properly contextualized. Some bloodstream infections require multiple cultures of blood to obtain a single CFU of bacteria for diagnosis. If K. pneumoniae is one such species, then vasodilation may not be a clinically translatable phenomenon – and vice versa.
2. In the Response to Reviewers document, the authors discuss findings from a previous study performed by the Bachman lab that identified K. pneumoniae factors contributing to bacteremia. The authors state that "no hits were found in the capsule polysaccharide" but this is not entirely true. Capsule polysaccharide genes were not present in the input of the Bachman study, so they are missing from the identified genes because they are not included in the study. More importantly for the present work, the Bachman lab study found no connection between T6SS and K. pneumoniae bacteremia pathogenesis. Since that supports the author's modified data, this should be discussed within the paper or at the very least cited. It's omission from the discussion is disappointing as this study helps to contextualize the present study within the pathogenesis field.
New Concerns:

1. T6SS is not activated in K. pneumoniae grown under standard lab conditions stated in the Materials and Methods, and can take a while to become activated under environmental pressure (PMID39843522). Given the short time period of the experiments in this present study, the authors should demonstrate that the T6SS is activated at the start of these experiments or during the experimental conditions. Otherwise, the effects due to vgrG4 mutations could be due to genetic functions unrelated to the T6SS. Additionally, the K. pneumoniae T6SS contains two loci. In the absence of the vgrG4 system, the other loci may still be active. If T6SS machinery is required but the system is off, then these genes would have novel unannotated function in vasodilation which would help contextualize these findings. The authors should discuss this in the manuscript and, at the very least, cite other T6SS papers for K. pneumoniae which investigate this virulence factor (PMID39843522).
2. In the paragraph with Text lines 170-175, the authors should call out where in the text their statements are supported. For example, which experiments conclude that K. pneumoniae blunts the NO pathway (Line 174)? And where are the in vivo results in question (Line 175)? These should be stated in the text to improve readability.
3. It is unclear why some dilation data is presented as the representative Outer Diameter Reads (Figure 1C-F) while others are presented as a Dilation curve (Figure 1H). This occurs in the supplemental data as well. The Outer Diameter Reads do not allow for statistical comparison or for showing multiple replicates and are difficult to read. The Dilation curves are much more intuitive.
4. Figure 2A should be updated to resolve differences between the delta-tssB and delta-vgrG3com strain. As is, I cannot tell which lines corresponds to which strain in the present panel.
5. In line 257-259, the authors state that “in the cells infected with either the tssB mutant or YeVgrG4 we did not observe any decrease in the agonist-induced Ser1177 phosphorylation”. This does not seem to be the case by eye. In fact, the tssB mutant appears to have no Ser1177 phosphorylation. This confusion is furthered by the lack of densitometry for Figure 4C. Can the authors please restate this sentence to increase the clarity of this section.
6. In the Discussion lines 441-443, the authors state that inhibition of vasodilation impairs the recruitment of immune cells into infected tissue. If the authors believe this to be the case for K. pneumoniae, then how do these findings fit within the field’s previous findings that Klebsiella infections are highly inflammatory and often lead to sepsis? It is difficult to connect these two points. This goes to a larger concern that the data is not fully contextualized within the Klebsiella pathogenesis field (see prior comments about no citations for other Klebsiella bloodstream infection studies or T6SS studies).
Comments on Statistical Analysis:
Throughout the manuscript, statistics are not adequately applied. Error bars are not always defined in the figure legends (Figure 1I, Figure 2B – there are no error bars on some of the points, Figure 4 densitometry, Figure 5 densitometry, Figure 6 densitometry, Figure 7B, Figure 7D-F). In many cases, there is also a lack of description on whether dots represent technical or biological/independent trials. The biggest concern on statistics surrounds the comparisons that were made, which is detailed in Point 1 below.
1. In Figure 4, statistics should be applied for the densitometry comparing the control to the Kp strain in question: for example, bradykinin control vs. bradykinin Kp43816. As is, the statistics are just demonstrating that the agonists worked and not that Kp is inducing a meaningful difference. This comment also extend to the densitometry in subsequent figures.
2. Figure 1G, the authors state in the figure caption that pathology is being scored. What are the pathology markers being observed? Is this damage, death, infiltration?
3. In the legend for Figure 3, the differences between panels Bi and Bii should be detailed for the reader. It is unclear how the traces in Bi differ from those represented in 3C.
4. In Figure 3D, the authors state in the caption that a two-way ANOVA is performed, but this would not be the case for such a data set. Should this be a paired t-test?
5. In Figure 3E-F, statistics are lacking. It is unclear if these results would be significant or if they were measured and are not significant. If measured, the authors should either indicate ns above the comparison or state that it was measured but it not displayed in the legend.
6. Figure 4C is missing densitometry but it is included in the figure caption.
7. In figure 5B, the densitometry plots only show two replicates.
8. In the new mouse data in Figure 1 and Figure 7, please indicate whether two independent trials were performed for each experiment. A single infection is not generally considered a robust trial as inoculum and animal cage variables can influence day-to-day factors such as stress and blood pressure.

Reviewer #3 (Remarks to the Author):

The authors have adequately addressed my concerns, although I will comment that the use of historical data to support the idea that the vessels are still able to dilate is less than optimal from a rigor and reproducibility standpoint. I have no further comments.

Reviewer #4 (Remarks to the Author):

The authors have substantially strengthened the manuscript and should be commended for the rigorous experimental work completed during revision. The addition of the CPS/vgrG4 double mutant and its phenotypic characterization clearly defines the additive and concurrent roles of the T6SS/VgrG4 and CPS pathways, resolving a major mechanistic gap in the original submission. The new in vivo blood pressure data greatly enhance the translational relevance of the study, and the expanded analysis of EDH impairment, including the NS309 experiments, provides a more complete mechanistic understanding of mtROS-dependent endothelial dysfunction.

****

Version 2:

Reviewer comments:

Reviewer #2

(Remarks to the Author)
The authors have been thorough in their responses to my major concerns.

Reviewer #4

(Remarks to the Author)
The authors have thoroughly addressed the critiques raised by all reviewers and have significantly improved the manuscript. Specifically, they have added extra citations, clarified the text, and revised figures and statistical analyses where needed in response to Reviewer #2's concerns.
Regarding the concern raised by Reviewer #2 on whether the T6SS is active under the laboratory conditions used in this study, prior work from the authors has demonstrated that the VgrG4 effector is actively delivered into host cells under standard laboratory conditions and is capable of modulating host cellular signaling networks (Sá-Pessoa et al., 2023, PMID: 36797302). Based on the authors' established track record, it is reasonable to conclude that the Klebsiella T6SS is functional under laboratory conditions.
While the current study uses a different host system (vascular endothelial cells and ex vivo blood vessel models) and, in part, different experimental settings, the authors present strong genetic and functional evidence supporting the involvement of T6SS and VgrG4 in the observed phenotype. Specifically, the use of deletion mutants, genetic complementation, and heterologous delivery of VgrG4 via a T3SS system collectively show that VgrG4 is both necessary and sufficient to mediate the effects on eNOS phosphorylation and vasodilation. These experiments significantly reduce concerns about T6SS activity under the tested conditions.
To further strengthen the manuscript, the authors might consider providing direct evidence of T6SS activity under the specific experimental conditions used here, such as assessing Hcp secretion in the spent medium if suitable reagents are available. However, such an experiment would mainly serve to reinforce conclusions already well supported by existing genetic and functional evidence, and should therefore be viewed as optional rather than essential.
The authors provide multiple complementary lines of evidence, including genetic mutants and in vivo validation, that support their mechanistic conclusions. While simultaneous controls would represent the most rigorous approach, the use of historical control data in this context does not undermine the validity of the findings.
Overall, the manuscript presents compelling and well-supported mechanistic insights into how Klebsiella pneumoniae modulates vascular physiology.

Decision Letter:

Our ref: NMICROBIOL-25020468B

5th March 2026

Dear Dr. Bengoechea,

Thank you for submitting your revised manuscript "K. pneumoniae disrupts vasodilation by targeting eNOS post translational modifications via the type VI secretion system and the capsule polysaccharide" (NMICROBIOL-25020468B). It has now been seen by the referees and their comments are below. The reviewers find that the paper has improved in revision, and therefore we'll be happy in principle to publish it in Nature Microbiology, pending minor revisions to comply with our editorial and formatting guidelines.

If the current version of your manuscript is in a PDF format, please email us a copy of the file in an editable format (Microsoft Word or LaTex)-- we can not proceed with PDFs at this stage.

We are now performing detailed checks on your paper and will send you a checklist detailing our editorial and formatting requirements in about a week. Please do not upload the final materials and make any revisions until you receive this additional information from us.

Thank you again for your interest in Nature Microbiology Please do not hesitate to contact me if you have any questions.

Reviewer #2 (Remarks to the Author):

The authors have been thorough in their responses to my major concerns.

Reviewer #4 (Remarks to the Author):

The authors have thoroughly addressed the critiques raised by all reviewers and have significantly improved the manuscript. Specifically, they have added extra citations, clarified the text, and revised figures and statistical analyses where needed in response to Reviewer #2's concerns.

Regarding the concern raised by Reviewer #2 on whether the T6SS is active under the laboratory conditions used in this study, prior work from the authors has demonstrated that the VgrG4 effector is actively delivered into host cells under standard laboratory conditions and is capable of modulating host cellular signaling networks (Sá-Pessoa et al., 2023, PMID: 36797302). Based on the authors' established track record, it is reasonable to conclude that the Klebsiella T6SS is functional under laboratory conditions.
While the current study uses a different host system (vascular endothelial cells and ex vivo blood vessel models) and, in part, different experimental settings, the authors present strong genetic and functional evidence supporting the involvement of T6SS and VgrG4 in the observed phenotype. Specifically, the use of deletion mutants, genetic complementation, and heterologous delivery of VgrG4 via a T3SS system collectively show that VgrG4 is both necessary and sufficient to mediate the effects on eNOS phosphorylation and vasodilation. These experiments significantly reduce concerns about T6SS activity under the tested conditions.
To further strengthen the manuscript, the authors might consider providing direct evidence of T6SS activity under the specific experimental conditions used here, such as assessing Hcp secretion in the spent medium if suitable reagents are available. However, such an experiment would mainly serve to reinforce conclusions already well supported by existing genetic and functional evidence, and should therefore be viewed as optional rather than essential.
The authors provide multiple complementary lines of evidence, including genetic mutants and in vivo validation, that support their mechanistic conclusions. While simultaneous controls would represent the most rigorous approach, the use of historical control data in this context does not undermine the validity of the findings.
Overall, the manuscript presents compelling and well-supported mechanistic insights into how Klebsiella pneumoniae modulates vascular physiology.

Version 3:

Decision Letter:

18th June 2026

Dear Professor Bengoechea,

I am pleased to accept your Article "Klebsiella pneumoniae inhibits vasodilation through capsule and T6SS-dependent pathways" for publication in Nature Microbiology. Thank you for having chosen to submit your work to us and many congratulations.

Over the next few weeks, your paper will be copyedited to ensure that it conforms to Nature Microbiology style.

Once your paper is typeset, you will receive an email with a link to choose the appropriate publishing options for your paper and our Author Services team will be in touch regarding any additional information that may be required. Once your paper has been scheduled for online publication, the Nature press office will be in touch to confirm the details.

You may wish to make your media relations office aware of your accepted publication, in case they consider it appropriate to organize some internal or external publicity. Once your paper has been scheduled you will receive an email confirming the publication details. This is normally 3-4 working days in advance of publication. If you need additional notice of the date and time of publication, please let the production team know when you receive the proof of your article to ensure there is sufficient time to coordinate. Further information on our embargo policies can be found here:
https://www.nature.com/authors/policies/embargo.html

After the grant of rights is completed, you will receive a link to your electronic proof via email with a request to make any corrections within 48 hours. If, when you receive your proof, you cannot meet this deadline, please inform us at rjsproduction@springernature.com immediately. You will not receive your proofs until the publishing agreement has been received through our system

Due to the importance of these deadlines, we ask you please us know now whether you will be difficult to contact over the next month. If this is the case, we ask you provide us with the contact information (email, phone and fax) of someone who will be able to check the proofs on your behalf, and who will be available to address any last-minute problems.

Acceptance of your manuscript is conditional on all authors' agreement with our publication policies (see https://www.nature.com/nmicrobiol/editorial-policies). In particular your manuscript must not be published elsewhere.

Authors may need to take specific actions to achieve compliance with funder and institutional open access mandates. If your research is supported by a funder that requires immediate open access (e.g. according to Plan S principles or the NIH public access policy) then you should select the gold OA route, and we will direct you to the compliant route where possible. Because authors warrant under our subscription licensing terms that they haven't committed to licensing any version of their article under a licence inconsistent with the terms of our agreement – including the applicable embargo period – publication under the subscription model isn't

suitable for authors whose funders require no embargo.

If you have any questions about our publishing options, costs, Open Access requirements, or our legal forms, please contact ASJournals@springernature.com

An online order form for reprints of your paper is available at https://www.nature.com/reprints/author-reprints.html. All co-authors, authors' institutions and authors' funding agencies can order reprints using the form appropriate to their geographical region.

You can now use a single sign-on for all your accounts, view the status of all your manuscript submissions and reviews, access usage statistics for your published articles and download a record of your refereeing activity for the Nature journals.

To assist our authors in disseminating their research to the broader community, our SharedIt initiative provides you with a unique shareable link that will allow anyone (with or without a subscription) to read the published article. Recipients of the link with a subscription will also be able to download and print the PDF.

As soon as your article is published, you will receive an automated email with your shareable link.

P.S. Click on the following link if you would like to recommend Nature Microbiology to your librarian http://www.nature.com/subscriptions/recommend.html#forms

** Visit the Springer Nature Editorial and Publishing website at www.springernature.com/editorial-and-publishing-jobs for more information about our career opportunities. If you have any questions please click here.**

Open Access This Peer Review File is licensed under a Creative Commons Attribution 4.0 International License, which permits use, sharing, adaptation, distribution and reproduction in any medium or format, as long as you give appropriate credit to the original author(s) and the source, provide a link to the Creative Commons license, and indicate if changes were made.
In cases where reviewers are anonymous, credit should be given to 'Anonymous Referee' and the source.
The images or other third party material in this Peer Review File are included in the article's Creative Commons license, unless indicated otherwise in a credit line to the material. If material is not included in the article's Creative Commons license and your intended use is not permitted by statutory regulation or exceeds the permitted use, you will need to obtain permission directly from the copyright holder.
To view a copy of this license, visit https://creativecommons.org/licenses/by/4.0/

Reviewer #1 (Remarks to the Author):

This manuscript addresses a largely understudied aspect of Klebsiella pneumoniae pathogenesis: the bacterium's ability to disrupt vascular function by targeting the endothelial nitric oxide synthase (eNOS), thereby inhibiting agonist-induced vasodilation. While endothelial dysfunction has been extensively examined in the context of viral haemorrhagic infections (e.g., Ebola) and in bacterial sepsis more generally, few studies have dissected K. pneumoniae's direct mechanisms of impairing vascular function.

Overall, I think the study is robust and well-presented and does a good job at analysing the mechanistic aspects of the biology.

We appreciate the positive assessment of our work. We would like to emphasise that the topic addressed in this manuscript has been largely overlooked in the context of bacterial blood stream infections including pathogens such as *E. coli* and *S. aureus*. While some of the mechanistic insights are *Klebsiella* specific, we posit that broader principles are likely shared across infection models. We believe that the knowledge platform described in this manuscript will be of general interest to the wider research community and will stimulate additional work in this area of clinical relevance.

All comments were carefully considered, and we have acted as follows.

I feel a few presentation elements might improve reader experience:
Enhanced Timeline Schematics: A diagram illustrating the timeline of infection, addition of agonists, and sample collection in each experiment would help readers better follow the experimental steps.

A new schematic has been added to Figure 1 (new panel B) to present an overview of the experimental design. This complements the detailed description already included in the methods section.

Statistics and Replicates: Although the paper states the number of biological replicates in multiple sections, consistently emphasising technical replicates (e.g., n=3, each in triplicate) vs. biological replicates (e.g., 3 separate rats per group) would clarify the robustness of some of the data.

This information has now been added to each figure legend.

The discussion highlights many possible research expansions, but a concise "Future Directions" section could strengthen the discussion.

We have revised the Discussion section accordingly (line 513).

In addition, it's worth considering a few additional studies to strengthen the potential translational impact:
The ex vivo model is a powerful tool, but the study stops short of verifying this vascular dysfunction in vivo or in other vascular beds (e.g., pulmonary, renal, or cerebral microcirculation):
Testing in an animal infection model (e.g., mice or rats) to measure blood pressure, tissue perfusion, or immune-cell infiltration would confirm physiological relevance. This could be done, perhaps, by comparing wild-type K. pneumoniae versus T6SS/capsule mutants.
As suggested, we now include in vivo work measuring blood pressure upon infection (new Fig 1I). We observed a significant increase in MAP at 24 h post infection, with smaller, non-significant increases at 6 and 12 h post infection. Data is consistent with a significant difference in systolic and diastolic blood pressure at 24 h post infection (Fig S1M and Fig S1N). Importantly, no significant changes in the blood pressure were observed in PBS-mock infected mice, ruling out stress from the experimental set up as a contributing factor.

We did also run the suggested experiment testing the capsule, *vgrG4* and double capsule-*vgrG4* mutant (new Fig 7E). None of these mutants induced an increase in blood pressure. However, we do recognise that in the case of the capsule mutants this may be explained by the markedly reduced bacterial loads observed in organs (some mice even cleared the infection) (Fig 7F). This agrees with the well-established role of the capsule as the single most important virulence factor of *K. pneumoniae*. In contrast, bacterial loads in mice infected with the *vgrG4* mutant were not significantly different to those of mice infected with the wild-type strain, yet no increase in blood pressure was detected (Fig 7E and Fig 7F). This result further substantiates a key role for VgrG4 in modulating vascular physiology, consistent with our ex vivo findings.

In this study, we focused on mesenteric arteries to assess the effects of *Klebsiella* infection on eNOS and EDH signalling. While mesenteric arteries are not identical to pulmonary, renal, or cerebral vessels, they provide a well-established and representative model for studying microvascular endothelial cell function. The core mechanisms underlying vasoregulation, particularly eNOS and EDH pathways, are conserved across vascular beds, and the alterations we observed are therefore likely to reflect general endothelial dysfunction mechanisms relevant to multiple organ systems. Importantly, our in vivo blood pressure measurements suggest a broader effect on total peripheral resistance rather than one confined to the mesenteric circulation. We believe that extending these analyses to other vascular territories, while valuable, goes beyond the scope of the present study and would be best addressed in future work focused on tissue-specific differences.

It might be insightful to examine if the same T6SS-dependent phenomenon occurs in coronary, renal, or cerebral arteries, especially since (I believe) K. pneumoniae commonly causes disseminated infections.

Please refer to our previous answer. Please note that there are very few, if any, reports describing *Klebsiella*-triggered coronary or central nervous system infections, even in the pre-clinical models.

The study suggests potential avenues for host-targeted therapies, such as PKCβ inhibition or mtROS scavenging, to restore vasodilation:
Evaluating whether in vitro or ex vivo treatments combining bactericidal agents (e.g., colistin or carbapenems) plus a PKCβ inhibitor or MitoTEMPO enhances bacterial clearance or reduces vascular damage might appeal to clinicians.
A fuller characterisation of inhibitor efficacy, toxicity ranges, and potential off-target effects in a more physiologically relevant model could help transition these findings into animal models or early translational studies.

In the discussion, we aimed to highlight potential avenues for translational research, such as PKCβ inhibition or mitochondrial ROS scavenging, to catalyse further research in this area. We agree with the Editor that additional studies assessing inhibitor efficacy, toxicity, and off-target effects in more physiologically relevant models would be valuable but are beyond the scope of the present manuscript.

Reviewer #2 (Remarks to the Author):

In this study by Rehman, et al. the authors seek to define mechanisms by which Klebsiella pneumoniae influences vasodilation. Using a creative ex vivo approach utilizing rat tissue, the authors constrict blood vessels with the chemical phenylephrine (PE) and monitor vessel dilation with acetylcholine (ACh). In this system, they observe that K. pneumoniae inhibits blood vessel re-dilation after the ACh treatment. To further probe this finding, the authors use in vitro infection of human endothelial cells to characterize post-translational modifications within the eNOS pathway that correlate with inhibition of re-dilation. This study addresses vascular biology, an important aspect of K. pneumoniae infection that has not been investigated in detail in previous literature. While the authors should be commended for a mechanistic study about an understudied aspect of K. pneumoniae infection, I am concerned about the relevance of these findings to infection biology in an in vivo context, and I was not fully convinced of the findings surrounding capsule polysaccharide (CPS) limiting phosphorylation of Ser177 in eNOS.

We thank the reviewer for their constructive assessment of our manuscript and for recognising the value of the knowledge platform described. We appreciate their comments and have carefully considered all points raised. Our detailed responses and corresponding revisions are provided below.

Major Concerns:

1. The authors present an innovative method to monitor blood vessel constriction and dilation in an ex vivo manner. While this should be commended as it avoids the excess use of animals, I am not convinced that this model accurately reflects bacteremia pathology. First, systemic infections include the presence of cytokines, circulating immune cells, and serum which are all important for host-pathogen interactions in bacteremia and work in a coordinated manner. Without these factors present in the ex vivo model, it is difficult to understand whether the observations observed in this study are also present in vivo.

We agree that the ex vivo model cannot fully recapitulate the complex milieu of systemic infection. However, this limitation is inherent to most cellular and tissue-based infection models. Importantly, there is there is a wealth of evidence across the field showing that mechanistic insights gained from such reductionist approaches often translate well to in vivo settings (for example, in *Salmonella*, *Listeria* or *Mycobacterium* pathogenesis).

Our ex vivo observations provide valuable tissue-level evidence of *Klebsiella*-induced alterations in vascular physiology. Moreover, the mechanistic findings derived from infections of primary endothelial cells were consistent with the physiological outcomes observed in the ex vivo system (see Fig. 7E).

To further address the reviewer's concern, we have now included in vivo experiments (Fig 1I, Fig S1M, Fig S1N, (Fig 7E and Fig 7F) demonstrating that *Klebsiella* infection leads to hypertension, a well-established indicator of vascular function. The observed increase in blood pressure is consistent with our ex vivo findings, where *Klebsiella* infection inhibited eNOS and EDH signalling, thereby impairing vasodilation.

We have also added complementary data on the relevant mutants. Although the capsule mutant is attenuated in vivo, preventing us from conclusively determining its contribution to vascular physiology under these conditions, we observed that mice infected with the *vgrG4* mutant exhibited bacterial loads comparable to those infected with the wild-type strain but did not show an increase in blood pressure. This finding supports the key role of *vgrG4* in modulating vascular physiology, in line with our ex vivo data. These new results further strengthen the mechanistic link between the T6SS effector VgrG4 and impaired endothelial function, while reinforcing the translational relevance of our ex vivo and cellular observations.

Second, during Gram-negative bacteremia, bacteria are rarely found in the bloodstream itself and are quickly sequestered to organs like the liver.

We do not disagree with this comment, which is based mostly on observations from mouse models of infection (specifically C57BL/6 mice challenged via tail vein injection). However, clinical evidence clearly demonstrates the presence of *Klebsiella* in the bloodstream of patients, as confirmed by culture methods, the gold standard diagnostic method for bloodstream infections. In some cases, *Klebsiella* can be cultured from blood samples for more than ten consecutive days. Therefore, our research is highly relevant to *Klebsiella*-associated bloodstream infections in humans.

Third, the authors should contextualize their findings considering other K. pneumoniae bacteremia pathogenesis work. In a recent study (PMID37463183), the authors used transposon sequencing in ATCC43816 (the same strain used in the present study) to identify K. pneumoniae factors important for survival during in vivo bloodstream infection. In that study, all T6SS genes were dispensable for infection. Thus, although the data in Figure 2 is compelling that the T6SS influences vasodilation ex vivo, due to this pre-existing study, I am not sure that the T6SS specifically impacts K. pneumoniae pathogenicity in the bloodstream. To address this, the author could consider competing their T6SS mutant against WT K. pneumoniae in an intravascular model of bacteremia to demonstrate that the T6SS mutant is less fit during infection.

The elegant study from the Bachman lab cannot be strictly considered an assessment of bacteraemia fitness, as the authors used bacterial recovery from the spleen as the readout. As noted in their own manuscript, this measurement reflects a combination of factors, including the sum of bacterial fitness in the blood, bacteria reaching the organ, and bacterial susceptibility to innate defences of the spleen. The study also reported significant differences in mouse mortality (0-70%) and, therefore, only a subset of samples was subsequently analysed. Intriguingly, no hits were found in the capsule polysaccharide despite its well-established role as a major virulence determinant, regardless of infection route. In our study (Fig 7F) we show that the capsule mutant is attenuated in systemic infection, as evidenced by bacterial recovery from the spleen, liver, and lungs following intraperitoneal infection. Therefore, the absence of T6SS hits in the transposon screen should not be interpreted as evidence that the T6SS does not contribute to bacteremia fitness.

Finally, we would like to emphasise that our model was not designed to assess bacterial fitness per se, but rather to define the impact of *Klebsiella* infection on vascular physiology.

2. The conclusion from Figure 4 is unclear. The authors suggest that Kp43816 inhibits

Ser177 phosphorylation that is induced by eNOS agonists, but this is not apparent by eye. From the raw blot in 4B, it appears that the entire gel lane for the uninfected wells may be darker than the lanes in the Kp43816 lanes, which would make the densitometry calculation in 4B appear higher in the uninfected wells (since the numerator would be a higher value due to a darker lane). These results seem very subtle, and by eye they are undetectable. I am not convinced that this conclusion is supported by the data without a secondary means of confirmation such as knockout cell lines or a reporter assay.

We respectfully disagree with this comment. We firmly believe Fig4B and 4C clearly demonstrate the effect of the agonists on Ser1177 phosphorylation (as seen when compared to non-treated cells) and the absence of this response in cells infected with either the wild-type strain or the *t6ss* mutant. As noted in the legend and methods section, the quantification presented is based on data from three independent blots, not solely the one shown in the figure. Nonetheless, we have now included an updated Fig 4B to further clarify this point.

3. Why did the authors choose the timepoints that are used in this study? The results in this study are shown over several hours, but did the authors consider a more prolonged timepoint to see if protein modifications are more dramatically altered later during infection? For example, can the T6SS initiate signaling within minutes? Is it possible that T6SS mutants would be able to influence post translational modifications at longer timepoints, like 24 hours?

Please note that the vascular physiology readouts are obtained in real time, as illustrated by the traces included in the manuscript. The analysis period does not extend beyond 30 minutes. During model optimisation, we performed control experiments to determine an infection duration that produced a reproducible phenotype while maintaining vessel viability. Infection-induced effects on vasodilation could be detected within 15–20 minutes, with consistent and reproducible results across vessels achieved after 60 minutes of infection.

The time points selected for the immunoblot experiments are standard in the field. For reference, similar infection durations have been used in studies of *Klebsiella* (PMID: 35947948) *Legionella* (PMID: 28867389) *Salmonella* (PMID: 19779561and PMID: 33603205), and *E. coli* (PMID: 17339360). These studies, along with our own data, demonstrate that infection can induce sustained effects on post-translational modifications over this timeframe.

Unfortunately, extending the infections to 24 hours is not feasible in our system, as vessel viability declines.

Minor Concerns:
1. In Line 154, please update the phrasing to say that "inhibition of validation is observed across multiple diverse strains of K. pneumoniae." The authors could also consider

noting that the different strains of K. pneumoniae produce very different effects in this figure. Thus, some K. pneumoniae strains may inhibit dilation to a higher degree than others, which is an interesting finding.

We have changed the text as suggested (line 160.

2. The results of Panel 3D are statistically significant but very subtle. Can the authors consider adding lines for pairwise connection of trials to demonstrate a consistent increase in Ca2+ response during each repeat?

We have done as suggested, please refer to new fig 3.

Reviewer #3 (Remarks to the Author):

This manuscript describes the effects of infection of endothelial cells with K. pneumoniae on endothelium dependent vasodilation and on signal transduction pathways that appear to mediate inhibition of endothelium-dependent vasodilation using cultured human endothelial cells. Overall this is a well conducted study that clearly outlines the effects of K. pneumoniae infection on endothelium dependent vasodilation and identification of the signaling pathways involved in this inhibition.

We thank the reviewer for her/his constructive insights into our work. We have acted as follows.

I only have two significant concerns. The first relates the authors statement that K. pneumoniae infection has no effect on endothelium apparently based solely on histological examination of vessel longitudinal sections. It is not clear to this reviewer that it is possible to assess, for example, the Barries function of the endothelium using this simple approach. It is suggested that a more rigorous approach be utilized to assessed endothelial structural integrity, or that this statement be softened.

To add additional evidence supporting our conclusion that *Klebsiella* infection does not damage the endothelium, we have included new functional data. After infection, vessels were treated with ciprofloxacin to kill both extracellular and intracellular bacteria, after which acetylcholine was added to assess vasodilation. As shown in Fig S1I, acetylcholine induced vasodilation in ciprofloxacin-treated vessels, with responses comparable to those observed in non-infected vessels. In contrast in control experiments in which the endothelium is damaged by air bubbles, a well-established approach in the field, acetylcholine failed to induce vasodilation.

We have also added data showing that *Klebsiella* infection does not affect endothelial cell viability (Fig S1K). Altogether, this new evidence, combined with our histopathology analysis, provide strong evidence that *K. pneumoniae* infection does not damage the endothelium. Additionally, the activation of calcium signalling at the single cell level in

the endothelium upon infection (Fig 3) further supports the absence of structural damage.

Second, while the authors clearly show that K. pneumoniae infection blunts EDH mediated dilation and that Mito-ROS is involved, they have not shown how this blunting is accomplished. Does K. pneumoniae infection reduce dilation mediated by NS-319 or other IKCa/SKCa agonist or IKCa and/or SKCa currents in isolated endothelia cells? Such findings would better "close the loop" for this aspect of the authors findings.

We have now performed the suggested experiment using the SK/IK channel activator NS309 (Fig 7B, Fig S7E, Fig S7F, Fig S7G). As expected, NS309 induced vasodilation in non-infected vessels. In contrast, *Klebsiella* infection markedly attenuated NS309–induced vasodilation, and this effect was restored by treatment with MitoTEMPO. These results provide additional mechanistic evidence that *Klebsiella* infection disrupts EDH-mediated vasodilation through a mitoROS-dependent mechanism.

Our findings suggest that mitoROS may impair EDH signalling either by directly affecting Ca^{2+}-activated potassium channels or by altering endothelial–smooth muscle coupling downstream of these channels' activation. This new mechanistic work has allowed us to rule out any *Klebsiella* effect upstream these channels including, for example, endothelial muscarinic receptors, trimeric G-proteins, IP3 pathway, Ca2+ store release, Ca2+ influx pathways. We believe that further mechanistic dissection of this pathway falls beyond the scope of the current study but will be of interest for future work. We foresee that this follow-up work may take more than 3-4 years, and it would involve patch clamp experiments, single channel isolation, and dual electrode electrophysiology to start with.

Reviewer #4 (Remarks to the Author):

1. Summary of the Study
In this study, the authors establish a robust ex-vivo vascular model to investigate how Klebsiella pneumoniae infection affects vasodilation. Using rat mesenteric arteries and human endothelial cells, they show that K. pneumoniae impairs acetylcholine-induced vasodilation by targeting nitric oxide (NO) and endothelium-derived hyperpolarization (EDH) pathways. Mechanistically, they identify two parallel bacterial strategies: (1) the trans-kingdom T6SS effector VgrG4 induces mitochondrial ROS (mtROS) in an NLRX1-dependent manner, activating PKCβ and phosphorylating the inhibitory Thr495 site of eNOS, and (2) the capsular polysaccharide (CPS) activates an EGFR-dependent signaling cascade, leading to PP2Ac upregulation and dephosphorylation of the activating Ser1177 site of eNOS. Both modifications blunt eNOS activity and reduce NO availability, thereby inhibiting vasodilation. These mechanistic insights were further validated functionally, as pharmacologic inhibition of mtROS or PKCβ, and using a CPS-deficient mutant, partially restored vasodilation, and their combination fully restored

vascular response. The study reveals a previously unappreciated role of T6SS and CPS in manipulating host vascular physiology through post-translational control of eNOS. By addressing a poorly explored niche in host-pathogen interaction, the authors fill an essential gap in our understanding of bloodstream infections. Moreover, they propose a novel role for NLRX1 and mtROS signaling in vascular dysfunction, opening doors for future investigation into host-directed therapies.

2. Evaluation of Experimental Evidence

The authors present a well-structured, multi-layered experimental framework. From molecular dissection in human endothelial cells to functional validation in ex vivo blood vessels, the study convincingly demonstrates how K. pneumoniae impairs vasodilation. The evidence for both VgrG4- and CPS-dependent modulation of eNOS is comprehensive and methodologically sound. Importantly, pharmacologic and genetic tools converge to support the authors' claims. However, certain mechanistic connections, particularly between the two pathways—are treated independently without defining whether they act redundantly, synergistically, or temporally apart. The study's conclusions are strong overall, but a few clarifications would enhance interpretability and ensure completeness.

We thank the reviewer for their positive assessment of our work and the valuable suggestions. We have acted as follows.

3. General Critique and Conditional Recommendation

This compelling and timely study combines elegant mechanistic insight with a novel physiological model. The experiments are exceptionally well designed, and ex vivo blood vessels add translational value. However, several points merit refinement. The manuscript structure occasionally feels fragmented, with abrupt shifts between the T6SS and CPS narratives. While the functional data indicates additive roles, the relationship between the two pathways remains undefined, and a more explicit discussion of this point would help unify the findings. Similarly, the VgrG4 pathway appears sufficient to block eNOS activity; the rationale for invoking the CPS–EGFR–PP2Ac axis could be more clearly justified beyond redundancy. Although the use of mutants and inhibitors is strong, the absence of genetic complementation for ΔvgrG4, ΔtssB, and ΔmanC somewhat limits the conclusiveness of these loss-of-function results. A CPS^{-}/$T6SS^{-}$ double mutant would have further solidified the independence or interaction of the two mechanisms. While technically reasonable, using HULEC-5a cells stimulated with histamine deviates from the ACh–M3–eNOS axis and may not reflect vascular physiology as directly as the ex vivo system. Lastly, while the inhibition of EDH is functionally supported, the underlying molecular mechanism remains speculative. That said, the novelty and depth of this study are undeniable. The authors propose an innovative mechanism whereby K. pneumoniae licenses host homeostatic enzymes (PKCβ and PP2Ac) to suppress eNOS activation. This strategy represents a paradigm shift from direct effector-target interactions to subtle manipulation of host regulatory

circuits. Furthermore, suggesting that K. pneumoniae-induced vascular dysfunction resembles cardiovascular disease adds translational appeal.

4. Reviewer Questions for Clarification

1. Are the T6SS and CPS pathways functionally redundant, complementary, or temporally distinct?

Clarifying their relationship would greatly aid in interpreting the biological significance of the dual mechanisms.

2. Why was a CPS⁻/T6SS⁻ double mutant not included?

Although pharmacologic rescue and mutant-inhibitor combinations support their additive effects, a genetic double mutant would offer more conclusive validation.

We have now tested a double mutant lacking both the capsule and VgrG4 (Fig 7D and Fig S7O). This mutant showed no effect on acetylcholine-induced vasodilation, demonstrating that the NO and EDH pathways are not impaired and supporting an additive relationship between the two mechanisms.

Together with the other results presented in the manuscript, these findings reinforce our conclusions. VgrG4 is necessary and sufficient to inhibit vasodilation due to the induction of mtROS targeting the EDH pathway and the inhibition of the NO pathway by inducing the phosphorylation of the inhibitory Thr495 site of eNOS. In parallel, the capsule contributes to the inhibition of eNOS activity by inducing dephosphorylation at the activating Ser1177 site. Evidence from both the ex vivo model and the primary cell lines indicates that these two processes occur concurrently, supporting a coordinated mechanism of vascular dysfunction.

3. Why was HULEC-5a used for histamine-induced Ca^{2+} imaging rather than a model that retains ACh responsiveness?

Could Ca^{2+} signaling have been assessed in the ex vivo model for physiological alignment?

We would like to clarify that Hule-5a cells challenged with histamine were not used for the imaging, but to assess calcium signalling in cells infected with *Klebsiella*. We did report in the previous version of the manuscript the experiments suggested by the reviewer in which the calcium signalling was assessed in the ex vivo model (Fig 3).

4. How general is this vasodilation inhibition strategy?

Has the same phenotype been tested with other bloodstream pathogens such as E. coli, Pseudomonas, or Vibrio vulnificus?

We agree with the Editor that testing additional pathogens, while exciting, is beyond the scope of the present manuscript. This work is ongoing, and our preliminary data indicate that invasive *Salmonella* also impairs vasodilation in the ex vivo model via a

T3SS-dependent mechanism, whereas *Pseudomonas* seems to exert a cytotoxic effect on the endothelium. All in all, these early observations reinforce the value of the ex vivo model for investigating how different pathogens impact vascular physiology.

5. Can the vascular dysfunction observed here directly link to host mortality or impaired immune cell trafficking in vivo?
This would substantiate the translational relevance of the findings.

In this revised version, we now include in vivo data demonstrating the effect of *Klebsiella* on blood pressure, a well-established read-out of vascular physiology, as well as the specific contribution of VgrG4 to this response.

5. Suggested Additional Experiments (if feasible)

• Genetic complementation of key mutants

Reintroducing vgrG4, tssB, and manC under native promoters would strengthen the genetic conclusions.

We have run the experiments with complemented strains reported in Fig 2, Fig S2E, Fig S4F, and Fig S6B.

• Construction and analysis of a $CPS^{-}/T6SS^{-}$ double mutant

This would directly test whether both pathways are necessary for full eNOS inhibition.

Note our previous answer.

• ACh-induced Ca^{2+} signaling assessment in ex vivo vessels

This would offer a more direct link between intracellular signaling and physiological outcomes.

Note our previous answer.

• Broader pathogen comparison

Including other sepsis-causing bacteria would help determine the uniqueness of K. pneumoniae's strategy.

Note our previous answer.

• Mechanistic dissection of EDH suppression by mtROS

Investigating ion channel function or gap junction integrity would clarify how mtROS blunts hyperpolarization.

In the revised version, we include experiments demonstrating the effect of *Klebsiella*-induced mitROS on vasodilation triggered by the SK/IK channel activator NS309. These results provide further mechanistic evidence that *Klebsiella* infection disrupts EDH-mediated vasodilation through a mitROS-dependent mechanism and lay the groundwork for future studies on how mitROS influences Ca^{2+}-activated potassium channels and endothelial–smooth muscle coupling, which we believe lie beyond the scope of the present study. Please also refer to our answer to reviewer 3 on this same question.

Reviewer #1 (Remarks to the Author):

The authors have been highly responsive to the reviewer comments and have substantially strengthened the manuscript. In particular, the addition of in vivo blood pressure measurements, the inclusion of relevant mutant analyses, improved figure schematics, clearer reporting of biological versus technical replicates, and expanded mechanistic experiments addressing EDH signaling significantly enhance the physiological relevance, clarity, and impact of the study.

The revised discussion appropriately contextualises the findings, clearly delineates scope, and outlines future directions without overextending the conclusions. Concerns regarding translational relevance and mechanistic completeness have been addressed to a level that is appropriate for the present study.

Overall, I am satisfied with the revisions and believe the manuscript is now robust, well-presented, and suitable for publication.
We thank the reviewer for her/his assessment of the revised manuscript and his/her positive comments. No edits/changes required.

Reviewer #2 (Remarks to the Author):

This review is in response to a revised manuscript submitted by Rehman and colleagues. In this revised manuscript, the authors incorporate new in vivo data, ex vivo data, and text modifications to strengthen their original manuscript. While the authors have addressed many critiques raised by the reviewers, some of my major comments remain unsatisfied from the original peer review. Additionally, I have a number of new points of concern which were missed upon the initial review which I believe are important considerations for the ultimate publication of the manuscript. My comments are outlined below into three sections: 1) comments regarding the revision, 2) new concerns, and 3) comments on statistical analysis.

We appreciate the reviewer's thorough assessment of the revised manuscript. Please note below a detailed answer to the points raised that do not require any additional experimentation.

Comments Regarding the Revision:
1. In the Response to Reviewers document, my original concern was that Gram-negative species like Klebsiella pneumoniae are difficult to recover from the blood – in both mouse tail vein injection models and in clinical settings. The authors rebut saying that Klebsiella is clearly found in the blood by clinical culture systems, but this is not justified with a citation. It is true that blood culture methods are a gold standard for clinical diagnosis of bacteremia but this rebuttal comment requires a citation

stating how readily abundant K. pneumoniae is in the bloodstream of affected patients so that the present study can be properly contextualized. Some bloodstream infections require multiple cultures of blood to obtain a single CFU of bacteria for diagnosis. If K. pneumoniae is one such species, then vasodilation may not be a clinically translatable phenomenon – and vice versa.

There is a wealth of published evidence demonstrating that *K. pneumoniae* can be readily detected in the bloodstream of affected patients using standard clinical methods. We highlight several studies from the last five years that focus on improving detection and the timing of blood culture positivity (PMID: 15004113; PMID: 34964298; PMID: 17942663; PMID: 28274205; PMID: 19392886; PMID: 32394266; PMID: 38106471; PMID: 39856543; PMID: 36805070; PMID: 39747735).

Time-to-positivity (TTP) in blood cultures is a clinically established prognostic marker for *Klebsiella* bacteraemia, as it is for other pathogens. Clinical studies report median TTP values around 12 h, with values below 7 h in patients with high bacterial loads (PMID: 38548677; 40808663; 19392886). Furthermore, combining blood culture with real-time PCR can reduce TTP to as little as 2 h (PMID: 15004113). These studies used a single inoculation of blood cultures, reflecting standard clinical practice.

While most clinical studies do not directly quantify bacterial load in CFU/ml, it is generally recognized that bacterial concentrations below 10 CFU/ml are difficult to detect, whereas concentrations exceeding 10^3 CFU/ml correlate with a mean TTP of approximately 12 h (PMID: 39073670). These ranges are comparable to the inoculi used in our ex vivo blood vessel model, supporting the clinical relevance of our experimental observations.

Importantly, this demonstrates that the vasodilation observed in our ex vivo model occurs under bacterial loads that are clinically relevant. The bacterial concentrations in human *Klebsiella* bloodstream infections are sufficient for reliable detection in standard blood cultures, indicating that the physiological phenomena we report are not artifacts of unusually high inoculi. Thus, our experimental observations provide meaningful insights that are translatable to the clinical context.

Taken together, this evidence provides a robust clinical framework for our study, confirming that *K. pneumoniae* is readily detectable in human bloodstream infections and that the bacterial loads used in our experiments are physiologically and clinically relevant.

2. In the Response to Reviewers document, the authors discuss findings from a previous study performed by the Bachman lab that identified K. pneumoniae factors contributing to bacteremia. The authors state that "no hits were found in the capsule polysaccharide" but this is not entirely true. Capsule polysaccharide genes were not present in the input of the Bachman study, so they are missing from the identified genes because they are not included in the study. More importantly for the present work, the Bachman lab study found no connection between T6SS and K.

pneumoniae bacteremia pathogenesis. Since that supports the author's modified data, this should be discussed within the paper or at the very least cited. It's omission from the discussion is disappointing as this study helps to contextualize the present study within the pathogenesis field.

Regarding the concern about capsule polysaccharide genes, we note that the input transposon library used in PMID: 37463183 does contain capsule genes, as the same library was previously used by the Bachman lab to identify *Klebsiella* lung fitness factors, in which capsule hits were detected (PMID: 26060277). In the PMID: 37463183 manuscript, we have not found any indication that the input library does not contain any capsule genes, or that these mutant were removed from the input library before infection. Importantly, in PMID: 26060277 T6SS-related hits were identified within the reported log2 fold changes and no capsule hits (see S1 Table, Tn-seq results), despite our reported in vivo data (Fig 7F).

We would like to emphasize that our study was designed to investigate the impact of *Klebsiella* infection on vascular physiology, rather than to assess bacterial fitness per se. In this context, T6SS is functionally relevant: our data demonstrate that T6SS contributes to measurable effects on vascular tone and endothelial function, showing a direct physiological consequence of T6SS activity. This provides a complementary perspective to prior studies focused on bacterial fitness and pathogenicity.

To provide appropriate context for readers, we have now cited PMID: 26060277 in the discussion (line 473-476) and clarified that T6SS, while not previously linked to bacteraemia pathogenesis, exerts physiologically significant effects in our vascular model. These observations highlight how T6SS contributes to host-pathogen interactions beyond bacterial fitness, supporting the biological relevance of our findings.

New Concerns:
1. T6SS is not activated in K. pneumoniae grown under standard lab conditions stated in the Materials and Methods, and can take a while to become activated under environmental pressure (PMID39843522). Given the short time period of the experiments in this present study, the authors should demonstrate that the T6SS is activated at the start of these experiments or during the experimental conditions. Otherwise, the effects due to vgrG4 mutations could be due to genetic functions unrelated to the T6SS. Additionally, the K. pneumoniae T6SS contains two loci. In the absence of the vgrG4 system, the other loci may still be active. If T6SS machinery is required but the system is off, then these genes would have novel unannotated function in vasodilation which would help contextualize these findings. The authors should discuss this in the manuscript and, at the very least, cite other T6SS papers for K. pneumoniae which investigate this virulence factor (PMID39843522).

We thank the reviewer for his/her insights into *Klebsiella* T6SS. We respectfully disagree with this comment, and we provide the following clarifications:

1. Demonstrating loss of phenotype in null mutants is widely regarded as the gold standard in the field for establishing the contribution of a secretion system to a given phenotype. This approach has been used for over 15 years across multiple systems, including *Salmonella*, *Shigella*, *Legionella*, *Coxiella*, and *Brucella*, among others. When a null mutant lacks a phenotype relative to the wild-type strain, this directly demonstrates both the involvement and activity of the secretion system. In our case, we have tested two null mutants in two different *Kebsiella* genetic backgrounds with the same phenotype (please refer to Figs 2A, Fig S2b, and FigS2C).
2. We have previously shown that the T6SS in this strain, and in NTUH-K2044, is functional under laboratory conditions and antagonizes bacteria (PMID: 32191774, Fig S8A)
3. Using this strain, we have demonstrated VgrG4-dependent effect on eukaryotic cells targeting the mitochondria (PMID: 36797302, please refer to Fig 2E). The incubation time for these experiments is similar to those used in the present manuscript. We have published the T6SS-dependent injection of VgrG4 into eukaryotic cells (PMID: 36797302, please refer to Fig 2A).
4. Complementation of the *vgrG*4 mutant, together with *Yersinia*-based experiments, demonstrates that VgrG4 is necessary and sufficient for the observed phenotype. These combined results rule out contributions from other effectors

The reviewer demands we should cite and discuss PMID: 39843522. In this manuscript we have followed the standard scholarly practice of citing the first report of T6SS function in ATCC43816 (PMID: 32191774 in 2020). The 2025 study PMID: 39843522 describes the contribution of Kp43816 T6SS to gut colonization and we have cited PMID: 39843522 in the context of gut colonization (line 479). Importantly, the first description of *Klebsiella* T6SS contribution to gut colonization was published in 2023 (PMID: 36598189).

Revier implies that in *Klebsiella* there are two T6SS systems by referring to the analysis done in PMID: 39843522. While *Klebsiella* contains multiple T6SS loci, our prior work (PMID: 32191774) demonstrates that the functional T6SS is a single system with components distributed across two or three loci. Detailed mechanistic insights into T6SS assembly, while of interest, are beyond the scope of this manuscript and do not affect the conclusions. Work in progress in the laboratory demonstrates that in *Klebsiella* there is one single T6SS but with different assemblies.

Taken together, there is substantial published and experimental evidence demonstrating that Kp43816 T6SS is active under our experimental conditions, that VgrG4 mediates the observed vascular effects, and that other loci do not contribute

to this phenotype. These findings provide a robust mechanistic basis for our conclusions, and alternative loci or off-target effects are effectively excluded.

2.*In the paragraph with Text lines 170-175, the authors should call out where in the text their statements are supported. For example, which experiments conclude that K. pneumoniae blunts the NO pathway (Line 174)? And where are the in vivo results in question (Line 175)? These should be stated in the text to improve readability.*

We appreciate the opportunity to clarify the intent of this paragraph. The section in question is a concluding paragraph of this section of the Results, as explicitly indicated by the opening phrase "*Combined, these data demonstrate*…". As is standard scholarly practice, such summary statements are intended to synthesize findings already presented and referenced earlier in the Results section, rather than to reiterate figure citations.

The conclusions that *K. pneumoniae* blunts the nitric oxide pathway and that these findings are supported by in vivo data are directly derived from experiments described and referenced in the preceding paragraphs. These data are fully documented in the relevant figures and text immediately prior to this summary paragraph.

We have carefully reviewed the text to ensure that each experimental result supporting these conclusions is clearly presented and appropriately referenced in its primary description. We have found no need to edit the text. We retain the concluding paragraph as a synthesis of those findings, consistent with standard presentation of Results sections in the field. We have followed similar approach in other sections of the Results across the manuscript (for example line 237-239; line 274-278; line 319-321, line 363-365).

3.It is unclear why some dilation data is presented as the representative Outer Diameter Reads (Figure 1C-F) while others are presented as a Dilation curve (Figure 1H). This occurs in the supplemental data as well. The Outer Diameter Reads do not allow for statistical comparison or for showing multiple replicates and are difficult to read. The Dilation curves are much more intuitive.

The outer diameter traces show real-time measurements of vessel diameter and are included to illustrate the dynamic vascular responses observed in our experimental setup. These traces provide direct visualization of response kinetics, including onset, stability, and reversibility of dilation, which are important parameters in vascular physiology. Figure 1B provides a schematic overview of the experimental setup, which is best appreciated in conjunction with the representative traces shown in Figures 1C–F. Importantly, these traces are not presented as standalone or qualitative data but rather constitute the primary measurements from which the dilation curves are derived, as described in the Methods section. Across the manuscript, we intentionally present both data formats to provide a complete depiction of the experiments: representative traces to convey physiological

dynamics, and aggregated dilation curves to allow quantitative and statistical analysis. This approach is applied consistently throughout the manuscript. For example, in Figure 2 we present dilation curves, while Figure S2 shows the corresponding representative traces derived from six vessels across three independent animals, as indicated in the figure legends and Methods.

We believe that this combined presentation enhances transparency and interpretability, while ensuring that all statistical analyses are based on appropriately aggregated datasets.

5.Figure 2A should be updated to resolve differences between the delta-tssB and delta-vgrG3com strain. As is, I cannot tell which lines corresponds to which strain in the present panel.

We have revised the colouring of the lines as suggested by the reviewer.

5.IIn line 257-259, the authors state that "in the cells infected with either the tssB mutant or YeVgrG4 we did not observe any decrease in the agonist-induced Ser1177 phosphorylation". This does not seem to be the case by eye. In fact, the tssB mutant appears to have no Ser1177 phosphorylation. This confusion is furthered by the lack of densitometry for Figure 4C. Can the authors please restate this sentence to increase the clarity of this section.

We agree that the text is confusing. The point to be made is that the *tssB* mutant still inhibited the agonist-induced phosphorylation of Ser^{1177} like the wild-type strain (Fig 4B and Fig 4C); and VgrG4 does not decrease any agonist-induced phosphorylation (Fiig 4D). We have revised the text for clarity (line 260-264). Now we do include the densitometry associated with Fig 4C.

In the Discussion lines 441-443, the authors state that inhibition of vasodilation impairs the recruitment of immune cells into infected tissue. If the authors believe this to be the case for K. pneumoniae, then how do these findings fit within the field's previous findings that Klebsiella infections are highly inflammatory and often lead to sepsis? It is difficult to connect these two points. This goes to a larger concern that the data is not fully contextualized within the Klebsiella pathogenesis field (see prior comments about no citations for other Klebsiella bloodstream infection studies or T6SS studies).

We agree that, from a clinical perspective, *K. pneumoniae* infections are well recognized as highly inflammatory and frequently associated with sepsis. However, this clinical presentation reflects the late stages of infection, characterized by high bacterial burdens and systemic immune activation, rather than the early phase of infection, during which the pathogen must first evade innate immune defences.

In this early context, there is substantial mechanistic evidence demonstrating that a defining feature of *Klebsiella* infection biology is the suppression or modulation of host inflammatory responses, a property shared by both hypervirulent and classical

strains (please refer to a recent review, PMID: 39884872; and in vivo studies, PMID: 36337046; 38578798; 35947948; 35413274). Our results do not contradict this conceptual framework; rather, they provide a mechanistic explanation for how early vascular dysfunction may contribute to dysregulated inflammation during infection.

Specifically, our data indicate that inhibition of vasodilation impairs efficient immune cell recruitment at early stages of infection. This does not imply an absence of inflammation; instead, it suggests that vascular dysfunction may delay or spatially misdirect immune cell trafficking, leading to ineffective local containment of the pathogen. Such impaired early responses are well recognized to predispose to excessive systemic inflammation and sepsis, rather than effective localized immunity.

Therefore, reduced vasodilation and impaired leukocyte recruitment may paradoxically contribute to the highly inflammatory phenotype observed in severe *Klebsiella* infections by promoting bacterial persistence, tissue damage, and subsequent systemic immune activation. Thus, our findings provide mechanistic insight into the inflammatory and septic outcomes widely reported for *K. pneumoniae* bloodstream infections.

We have revised the Discussion to clarify that our work addresses early vascular–immune interactions (line 447-449), a dimension that has been comparatively underexplored in the field. We believe this perspective complements existing pathogenesis studies and extends current understanding of how *Klebsiella* infections progress from local infection to systemic disease.

Comments on Statistical Analysis:
Throughout the manuscript, statistics are not adequately applied. Error bars are not always defined in the figure legends (Figure 1I, Figure 2B – there are no error bars on some of the points, Figure 4 densitometry, Figure 5 densitometry, Figure 6 densitometry, Figure 7B, Figure 7D-F). In many cases, there is also a lack of description on whether dots represent technical or biological/independent trials. The biggest concern on statistics surrounds the comparisons that were made, which is detailed in Point 1 below.

Fig 1I does include error bars in the panel, and in Fig 2B the apparent "lack" of errors bars in some points is because there are within the symbol size or too small to be noted. The point of lack of clarity between technical and biological trials was made before by reviewer #1 and in the submitted revised version we did address this issue. Again, we have carefully reviewed the legends and the Methods to ensure it is clear what we are reporting in each panel.

For the densitometry comparisons, we did choose to compare against the non-infected samples which in turn represents the base line read-out. Any lack of significance demonstrates that the infection does not have any effect. Following the reviewer request now we do also include the comparisons between infected

samples. Please note that the conclusions have not changed. We have updated the figure legends accordingly.

1.In Figure 4, statistics should be applied for the densitometry comparing the control to the Kp strain in question: for example, bradykinin control vs. bradykinin Kp43816. As is, the statistics are just demonstrating that the agonists worked and not that Kp is inducing a meaningful difference. This comment also extend to the densitometry in subsequent figures.

We have met this request. The figure legend of Fig 4 has been updated accordingly.

2. Figure 1G, the authors state in the figure caption that pathology is being scored. What are the pathology markers being observed? Is this damage, death, infiltration?

This information is reported in Table S3 which was already included in the first submission of the manuscript.

3. In the legend for Figure 3, the differences between panels Bi and Bii should be detailed for the reader. It is unclear how the traces in Bi differ from those represented in 3C.

Bi is an averaged image of the Ca^{2+} signal at baseline (before ACh addition) of an *en face* endothelial preparation loaded with Cal-520. Examples of single Ca^{2+} signal traces from the cell annotated in the top panel are shown below in response to ACh at concentrations of 3nM, 100nM and 3µM. All of the data shown in Figure 3 are matched, so the experiments were first run without infection, the bacteria was then added to the same preparation to cause infection, and responses to the 3 different Ca^{2+} concentrations were then elicited again. Therefore, we were able to show the responses in precisely the same cell in both the uninfected (green) and infected (red) states.

Bii shows heat map visualisations of the $\Delta F/F_0$ Ca^{2+} responses to each concentration of ACh under non-infected (green) and Kp43816-infected (red) conditions. Again, these are all shown for the same artery, which is the one shown in Figure Bi.

The legend in Figure 3 has been updated accordingly, and the labelling on the figure has been improved for clarity.

4. In Figure 3D, the authors state in the caption that a two-way ANOVA is performed, but this would not be the case for such a data set. Should this be a paired t-test?

We apologise for the confusion here. It was actually paired Student t-tests between non-infected and Kp43186-infected conditions that were performed, and the statistical significance is indicated by asterisks, $p < 0.05$ (*), <0.01 (**), <0.001 (***) or $p>0.05$ (ns) .

Figures 3 D-F have been updated accordingly.

5. In Figure 3E-F, statistics are lacking. It is unclear if these results would be significant or if they were measured and are not significant. If measured, the authors should either indicate ns above the comparison or state that it was measured but it not displayed in the legend.

Statistical significance on these figures is now indicated by asterisks, p < 0.05 (*), or <0.01 (**). <0.001 (***), or ns p > 0.05. Figures 3 E-F have been updated accordingly.

6. Figure 4C is missing densitometry but it is included in the figure caption.

Now this is included.
7. In figure 5B, the densitometry plots only show two replicates.

It does reflect the number of independent experiments done as noted in the legend.
8. In the new mouse data in Figure 1 and Figure 7, please indicate whether two independent trials were performed for each experiment. A single infection is not generally considered a robust trial as inoculum and animal cage variables can influence day-to-day factors such as stress and blood pressure.

We confirm that two independent experiments were done. In each of the experiments non-infected and infected mice were assessed. Now we do include this information in the corresponding figure legend.

Reviewer #3 (Remarks to the Author):

The authors have adequately addressed my concerns, although I will comment that the use of historical data to support the idea that the vessels are still able to dilate is less than optimal from a rigor and reproducibility standpoint. I have no further comments.

We appreciate the reviewer's evaluation of the revised manuscript, and the favourable feedback provided. No further revisions are necessary.

Reviewer #4 (Remarks to the Author):

The authors have substantially strengthened the manuscript and should be commended for the rigorous experimental work completed during revision. The addition of the CPS/vgrG4 double mutant and its phenotypic characterization clearly defines the additive and concurrent roles of the T6SS/VgrG4 and CPS pathways, resolving a major mechanistic gap in the original submission. The new in vivo blood pressure data greatly enhance the translational relevance of the study, and the expanded analysis of EDH impairment, including the NS309 experiments, provides a more complete mechanistic understanding of mtROS-dependent endothelial dysfunction.

We acknowledge and appreciate the reviewer's positive assessment of the revised manuscript. No additional modifications were required.
